# Spontaneous Long‐Range Order and Alignment in Phase‐Separated Dynamic Covalent Networks Using Discrete Siloxanes

**DOI:** 10.1002/anie.5399898

**Published:** 2026-07-31

**Authors:** Stefan J. D. Maessen, Tymen M. L. van der Leede, Anne B. Spoelstra, Bart W. L. van den Bersselaar, Bas F. M. de Waal, Johan P. A. Heuts, Anja R. A. Palmans

**Affiliations:** ^1^ Department of Chemical Engineering & Chemistry and Institute for Complex Molecular Systems Eindhoven University of Technology Eindhoven the Netherlands; ^2^ Department of Chemical Engineering & Chemistry and Center for Multiscale Electron Microscopy Eindhoven University of Technology Eindhoven the Netherlands

**Keywords:** alignment, actuator, dynamic covalent networks, ordered structures, phase separation

## Abstract

For the first time, dynamic covalent networks are prepared that show spontaneous alignment and long‐range hexagonal columnar ordering. This is a consequence of the phase‐separation between the siloxane linkers and dynamic covalent acylsemicarbazide bonds that induce hydrogen bonding. The use of discrete siloxane linkers is crucial, as networks based on disperse siloxanes do not give ordered or aligned network structures. Optimization of the siloxane length is key in achieving the most pronounced effect. Interestingly, the optimized ordered networks are obtained through simple solvent evaporation, allowing to introduce near‐perfect homeotropic alignment as imaged with transmission electron microscopy and corroborated with grazing incidence small angle x‐ray scattering. The ordered network has improved (thermo)mechanical properties compared to the disordered networks. Additionally, order in the system slows down the network dynamics and improves creep resistance. We prepare actuators in a straightforward fashion by combining ordered and disordered networks, removing the need for any alignment step and simplifying the overall process significantly.

## Graphical abstract

Spontaneously aligned, long‐range hexagonal columnar ordering is obtained in phase‐separated dynamic covalent networks by combining discrete siloxanes and dynamic acylsemicarbazide bonds. The ordered network shows improved (thermo)mechanical properties and creep resistance compared to the disordered network. The combination of these networks affords actuators without the need of an alignment step, simplifying the overall process.

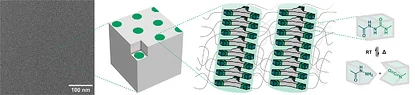

## 1 Introduction

Advances in functional polymer materials rely on precise molecular‐level control combined with favorable thermomechanical properties and straightforward processability [1, 2, 3, 4, 5]. As a consequence, phase‐separated block copolymers and liquid crystal networks have attracted significant attention as their long‐range ordering provides well‐defined mesoscale morphologies and the ability to align the ordered phases translates this order to anisotropic bulk properties and a broad range of functions [1, 2, 6, 7]. Such materials can respond to external stimuli such as electric fields, mechanical stress, light, and heat [8]. However, uniform alignment of the ordered structures typically requires optimization and the application of external fields or forces [7, 9]. Eliminating the need for an external alignment step through spontaneous alignment would offer a significant processing advantage. In addition to this, block copolymers often show limited mechanical robustness, whereas liquid crystal networks suffer from poor reprocessability due to the covalent crosslinks present within the network [3, 10]. Recent strategies to enhance mechanical performance and at the same time impart reprocessability in materials focus on incorporating dynamic covalent bonds (DCBs), which undergo bond exchange reactions in response to external stimuli [11, 12, 13, 14, 15, 16, 17]. The resulting dynamic covalent networks (DCNs) show tunable mechanical properties and reprocessability, but lack long‐range order and alignment [18, 19, 20, 21, 22].

In this study, we unite beneficial mechanical properties with order and spontaneous alignment in DCNs by combining the acylsemicarbazide (ASC) motif with discrete polydimethylsiloxane (pDMS) linkers (Scheme 1). ASCs are widely used DCBs that dissociate into hydrazides and isocyanates at elevated temperatures [23, 24]. Their hydrogen‐bonding properties allow for structural and morphological control in materials, especially when combined with hydrophobic pDMS, leading to phase‐separated DCNs [23, 25, 26]. To enhance long‐range order in these phase‐separated ASC‐pDMS networks, we here introduce siloxane linkers of uniform size, where every polymer chain has exactly the same number of repeat units. The application of such *discrete* polymers has previously drastically improved the long‐range ordering in block copolymer materials, yet is largely ineffective in polymer networks due to the crosslinked structure preventing optimization of ordered structures [27, 28, 29, 30]. In DCNs, however, reshuffling of bonds is possible, allowing the system to readjust and attain long‐range order. Previous research on the impact of discrete linkers in DCNs already showed the interesting effect on the relaxation behavior and diffusion coefficient in membranes, but no ordered structures were found [31, 32]. Here, we show the first long‐range ordered, spontaneously aligned DCN based on ASC‐pDMS and highlight its unique (thermo)mechanical properties. We further highlight the processing advantages enabled by spontaneous alignment through the straightforward fabrication of actuators from these networks.

**SCHEME 1:**
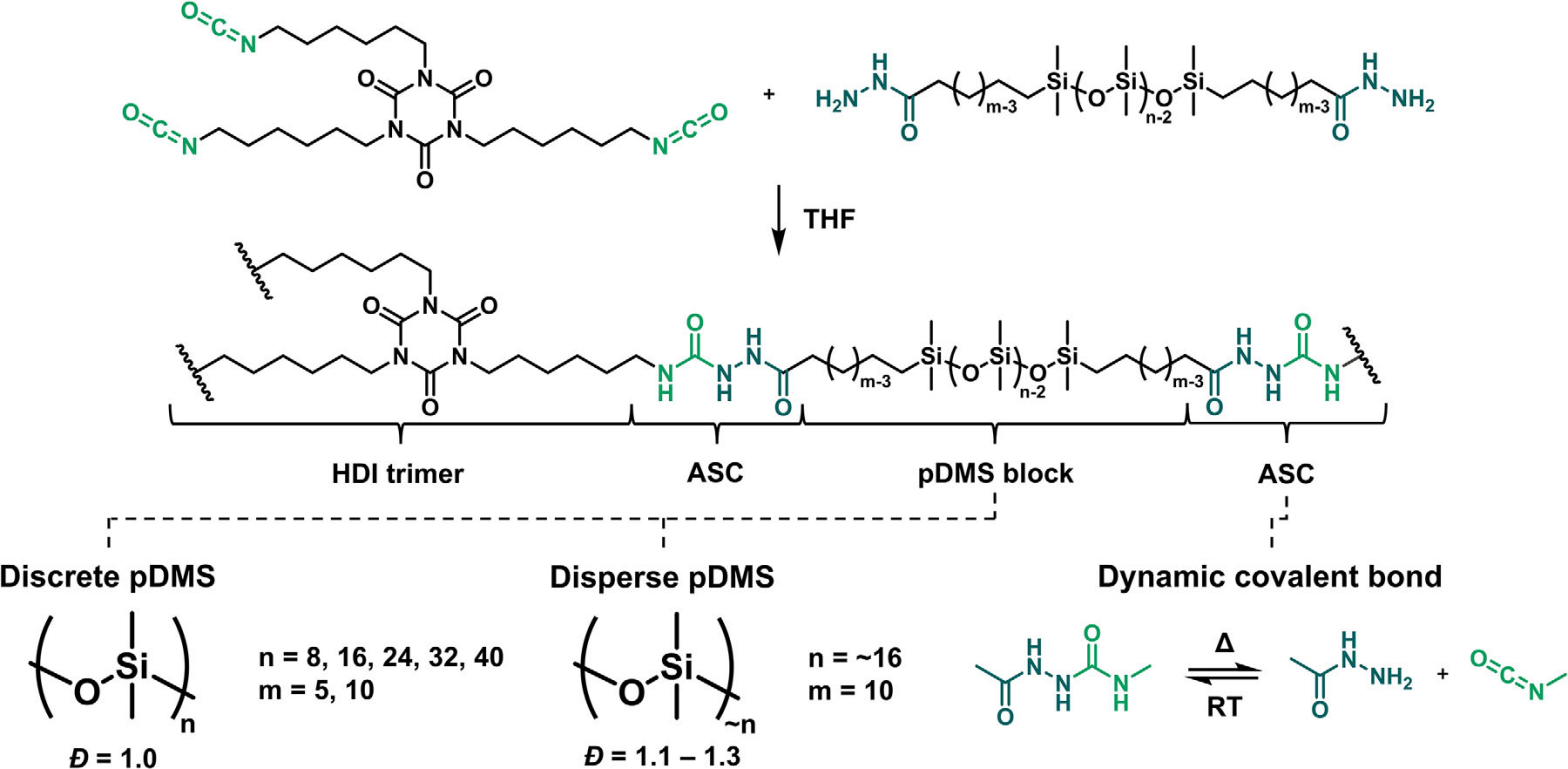
Synthetic pathway toward ASC‐pDMS networks using disperse and discrete pDMS dihydrazide prepolymers with various pDMS and alkyl spacer lengths.

## 2 Results and Discussion

ASC‐pDMS networks were prepared following our previous work [33]. First, hydrazide end‐capped pDMS prepolymers (discrete and disperse) were prepared in two steps by hydrosilylation of the corresponding pDMS dihydrides with alkene esters, followed by reacting the ester endgroups with hydrazine. The discrete pDMS dihydrides were obtained via an iterative strategy and comprised 8 (Si_8_), 16 (Si_16_), 24 (Si_24_), 32 (Si_32_), and 40 (Si_40_) siloxane repeat units [34]. Two different esters, methyl 10‐undecenoate (C_11_) and methyl 5‐hexenoate (C_6_), were used to investigate the effect of alkyl spacer length between the pDMS and ASC blocks. Disperse pDMS dihydrazide with an average of 16 siloxane repeat units (Si_∼16_) was also prepared to assess the effect of dispersity on the material properties. All polymer network films were made by mixing pDMS dihydrazide with (2,4,6‐trioxotriazine‐1,3,5(2H,4H,6H)‐triyl)tris(hexamethylene) isocyanate (HDI‐trimer) in THF, followed by rapid casting of the mixture in petri dishes at 50°C during which the ASC bonds form and the solvent evaporates (Scheme 1). The resulting films were cured at 160°C for 1 h. This strategy yielded flexible, transparent network films for all prepolymers, with mechanical properties similar to those reported in our previous work [33]. The networks will henceforth be named as their respective siloxane and spacer length (Si_n_‐C_x_), where n is the number of silicons, and x is the number of carbons in the spacer, including the carbonyl group.

The chemical mismatch between pDMS and ASC blocks induces microphase separation [33], which was investigated using medium‐ and wide‐angle x‐ray scattering (MAXS and WAXS). For the discrete networks Si_n_‐C_11_, the WAXS region (> 7 nm^−1^) shows two peaks at *q*  =  8.7 nm^−1^ and 14.4 nm^−1^, corresponding to distances between the pDMS backbone and aliphatic carbons, respectively (Figure 1A) [27]. The ratio between the two peaks changes upon increasing the number of siloxane repeat units, as expected. In addition, all networks show a clear principal scattering peak *q* in the MAXS region (< 7 nm^−1^), indicating phase separation. The domain spacing *d* (*d*  =  2π/*q*) of the phase‐separated domains increases with increasing siloxane length, from 4.8 nm for Si_8_‐C_11_ to 6.3 nm for Si_40_‐C_11_ (Table 1). Despite a fivefold increase in the number of siloxane repeat units, the domain spacing increases only by around 30%, most likely due to a less stretched conformation of the longer pDMS chains.

**FIGURE 1:**
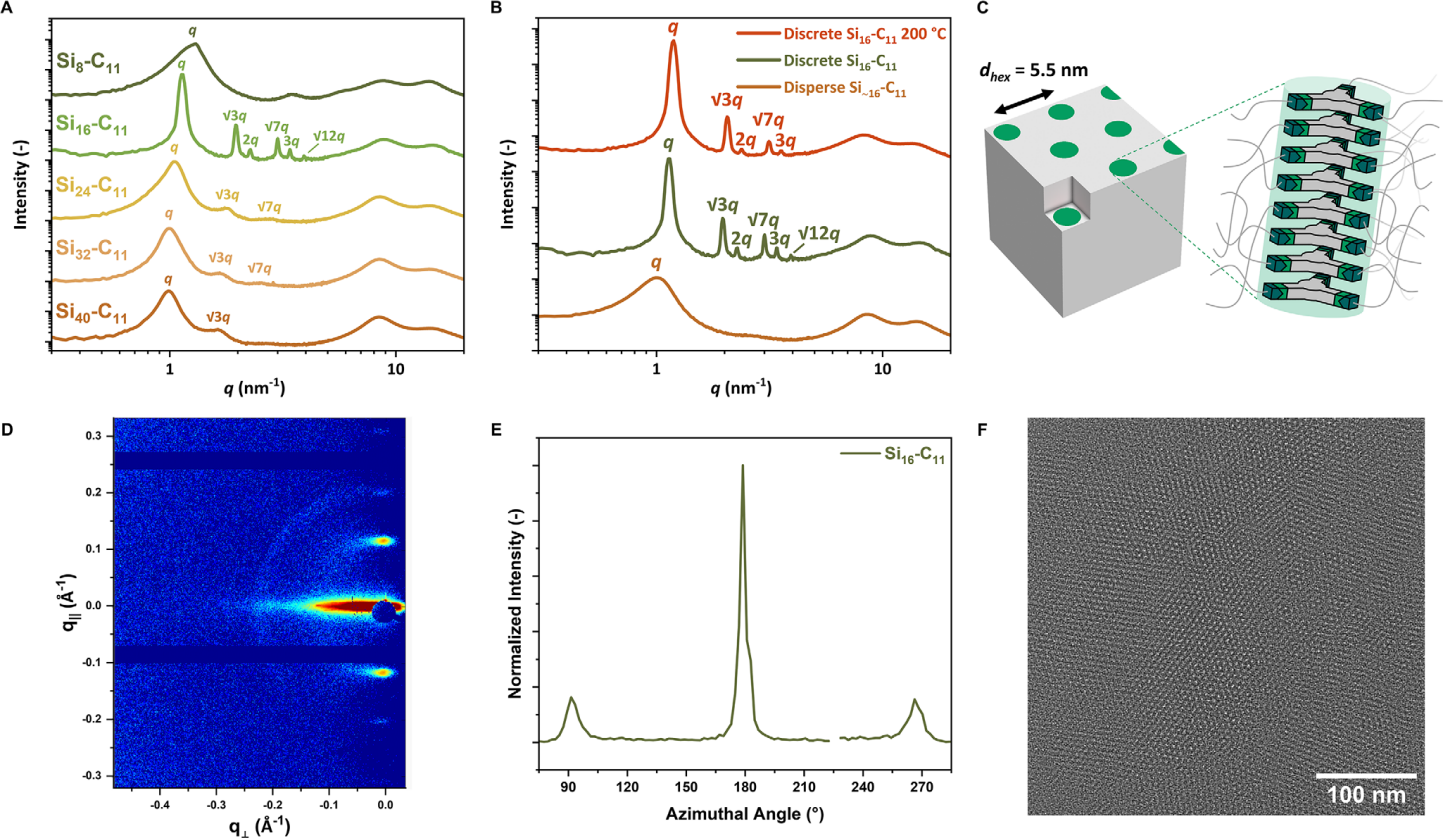
(A) MAXS and WAXS 1D transmission scattering profiles of cured Si_n_‐C_11_ networks at room temperature. (B) MAXS and WAXS 1D transmission scattering profiles of disperse Si_∼16_‐C_11_ network at room temperature and discrete Si_16_‐C_11_ network at room temperature and 200°C. (C) Schematic representation of HDI‐trimer stacks in ordered ASC networks. (D) 2D GIMAXS pattern of Si_16_‐C_11_. (E) Plot of the scattering intensity from the GIMAXS experiment of Si_16_‐C_11_ versus the azimuthal angle. (F) TEM image of Si_16_‐C_11_ sample prepared through microtomy.

**TABLE 1:** Values for the primary scattering peak q*, morphology, and domain spacings *d* of the networks in Figure 1A,B.

Network	q* (nm)	Morphology	*d* (nm)
Si_8_‐C_11_	1.31	Disordered	4.79
Si_16_‐C_11_	1.15	Col_h_	5.46
Si_24_‐C_11_	1.06	Col_h_	5.92
Si_32_‐C_11_	0.99	Col_h_	6.34
Si_40_‐C_11_	0.99	Col_h_	6.34
Si_∼16_‐C_11_	0.99	Disordered	6.34

Except for the Si_8_‐C_11_ network, all networks show reflection peaks with spacings corresponding to a hexagonal columnar packing (Figure 1A, Table 1). Remarkably, there are six sharp Bragg reflection peaks in Si_16_‐C_11_, pointing to a highly ordered structure, which are stable up to 200°C with only a minor decrease in peak intensity (Figure 1B). MAXS also shows that this high degree of order is not yet present before curing the films, suggesting that the dynamic nature of the bonds aids the process, as the ASC groups dissociate and can optimize the morphology (Figure S1). The networks with a shorter aliphatic C_6_ linker, Si_8_‐C_6_ and Si_16_‐C_6_, showed similar behavior to their C_11_ analogs. The intensity of the Bragg reflections is slightly lower for Si_16_‐C_6_ than for Si_16_‐C_11_, hinting that the additional length and flexibility of the alkyl spacer helps in ordering the system (Figure S2). Considering that the networks presented here are chemically crosslinked, the presence of such highly defined peaks is unprecedented. In fact, long‐range order in polymer networks is often prevented by the crosslinked nature of the network structure [28, 30]. Here, the observed order is most likely enabled by the dynamic covalent nature of the ASC bonds, allowing optimization and packing into the highly ordered structures. A visualization of the highly ordered 3D network with hexagonal columnar order in Si_16_‐C_11_ is presented in Figure 1C. We envision that the hexagonally packed columns consist of the trifunctional HDI‐trimer flanked by ASC groups, in a matrix of pDMS. The molecular design of the crosslinker and position of the ASC groups are likely leading the phase‐separated structure in this network. Other morphologies may be accessible by using other crosslinkers and/or discrete linkers.

The impact of a discrete versus a disperse siloxane linker in the network morphology was assessed by comparing the MAXS curves of the Si_16_‐C_11_ and the Si_∼16_‐C_11_ networks (Figure 1B). As previously reported [30], disperse Si_∼16_‐C_11_ networks show a phase‐separated structure, but lack long‐range order as is evident by the absence of Bragg reflection peaks. The difference in phase‐separated structure between the two types of network is remarkable, as the average crosslink densities of the discrete and disperse networks are the same, only the molar mass dispersity of the linkers connecting the crosslinked points differs. The IR spectra of Si_16_‐C_11_ and Si_∼16_‐C_11_ networks, in contrast, are almost identical, indicating no significant differences in the hydrogen bonding patterns of the ASC motifs in both networks (Figure S3). Furthermore, the N─H stretching vibration (∼3300 cm^−1^) in both networks have a bimodal peak before curing, while only a single, broad peak is present after curing, suggesting a decrease in hydrogen‐bonding order (Figure S4, S5) [33]. The N─H stretch peak broadening is counterintuitive for a network that shows an increase in long‐range order. Likely, the formation of ordered structures during curing is not governed by the hydrogen bonding capabilities of the ASCs, but rather a consequence of the uniform length of the perfectly discrete pDMS chains, allowing better packing than when there is variance in pDMS lengths in the disperse network [27].

To assess if there is alignment of the ordered structures within the network films, grazing incidence MAXS/WAXS (GIMAXS/WAXS) was performed. Strikingly, Si_16_‐C_11_ shows clear scattering rods perpendicular to the surface of the network film, on both the top and bottom of the film, indicating a spontaneous homeotropic alignment of the hexagonally packed columns throughout the network (Figures 1D, S6–S8). The normalized intensity of the primary and secondary Bragg reflections as a function of the azimuth angles also shows narrow peaks, with a full‐width at half maximum (FWHM) of the primary peak of 3.3° (Figure 1E). The FWHM is associated with the degree of alignment, where a value of 3.3° corresponds to near‐perfect alignment [35, 36]. The homeotropic alignment in combination with the high degree of order was further corroborated by visualizing the nanostructures through transmission electron microscopy (TEM) on samples prepared through microtomy (Figures 1F, S9, and S10). The TEM images show near perfectly ordered domains over large distances (>1 µm). The hexagonally spaced white dots (with spacings between dots of 5.5 nm, identical to the domain spacing from MAXS) correspond to the HDI trimer and ASC groups, and appear in a matrix of darker colored pDMS. While the exact origin of the spontaneous alignment is currently unclear, it is likely directed by solvent evaporation, where an ordered front is formed at the air/gel interface and propagates through the entire film [37, 38, 39]. These hexagonally ordered domains are absent in the disperse Si_∼16_‐C_11_ network (Figures S11 and S12).

Apart from a high degree of order and spontaneous alignment at room temperature, the discrete Si_16_‐C_11_ networks are also stable at high temperatures (Figure 1B). This high temperature stability allows to study the effect of order on the (thermo)mechanical and dynamic properties of the network. Dynamic mechanical thermal analysis (DMTA) of the Si_16_‐C_11_ and Si_∼16_‐C_11_ networks revealed similar thermal transitions for both, showing a *T*_g_ around −110°C, weakening of hydrogen bonds between 30°C and 80°C, and ASC dissociation above 170°C (Figure 2A) [33]. The main difference is observed in the position of the rubbery plateau above 80°C, where discrete Si_16_‐C_11_ shows a 3.5‐fold higher modulus at 120°C (11.6 MPa) compared to disperse Si_∼16_‐C_11_ (3.3 MPa). Despite both materials being phase separated, the ordered structures clearly bring additional mechanical robustness to the network. This is further evident from the tensile properties of the networks: discrete Si_16_‐C_11_ has a higher Young's modulus (13.2 ± 0.2 MPa) and lower strain‐at‐break (119 ± 13%) than disperse Si_∼16_‐C_11_ (10.9 ± 1.0 MPa and 167 ± 27%, respectively) (Figure 2B).

**FIGURE 2:**
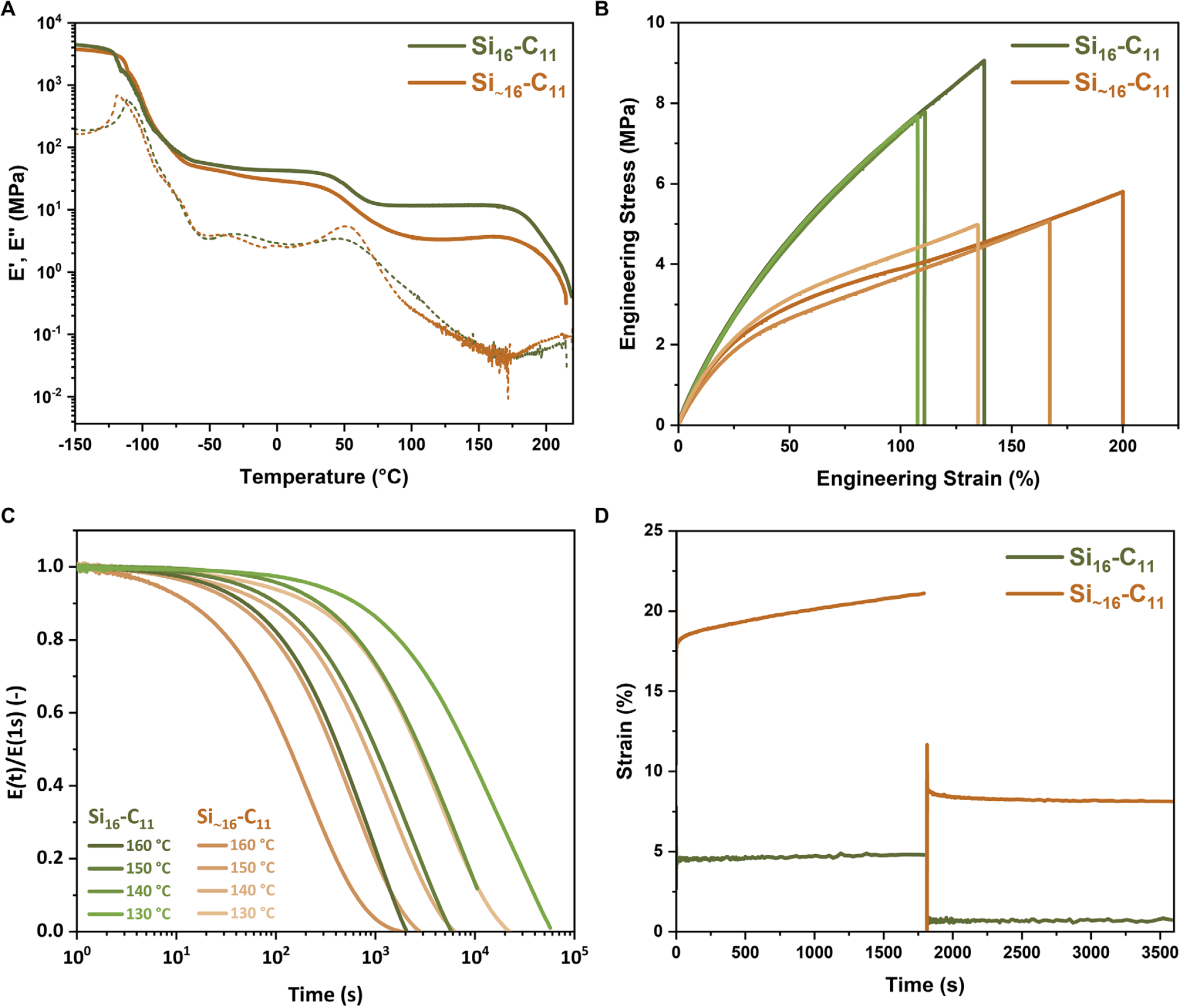
(A) DMTA curves of the cured ordered and disordered networks representing the storage (solid) and loss (dashes) moduli as a function of temperature. (B) Tensile test triplicates of cured ordered and disordered networks. (C) Stress relaxation curves in tension of the ordered (solid) and disordered (dashed) networks at various temperatures and 5% strain. (D) Creep‐recovery curves of ordered and disordered networks at 120°C with a stress of 0.5 MPa.

The influence of highly ordered phases on the dynamic covalent behavior of the ASC‐based networks was investigated by rheology using stress‐relaxation measurements between 130°C and 160°C. Both discrete and disperse networks relax stress due to dissociation of the ASC bonds causing reshuffling of network [23, 33]. The disordered Si_∼16_‐C_11_ relaxes stress faster than the ordered S_16_‐C_11_ (Figure 2C). To obtain the characteristic relaxation time *τ* and the stretching parameter *β*, the curves were fitted using a stretched exponential function, where *E*(*t*) is the relaxation modulus at time *t*:

(1) $$\frac{E\left(t\right)}{E\left(1s\right)}=\exp\left({\left(\frac{1}{\tau }\right)}^{\beta }-{\left(\frac{t}{\tau }\right)}^{\beta }\right)$$

The obtained *τ* and *β* were then used to calculate the *β*‐averaged characteristic relaxation time 〈𝜏〉 [40]. The characteristic relaxation times of Si_∼16_‐C_11_ are about three times faster than those of Si_16_‐C_11_ over the measured temperature range (Table 2, Figures S13 and S14). The activation energies of flow were calculated from the slope of an Arrhenius plot and were 141 and 150 kJ/mol (calculated from τ, 142 and 157 kJ/mol calculated from 〈𝜏〉) for Si_∼16_‐C_11_ and Si_16_‐C_11_, respectively (Figures S15 and S16). These values are within experimental error and comparable to previously reported values, both in model small molecule studies and polymer networks, showing that the chemistry of the two materials is also the same [23, 33, 41].

**TABLE 2:** Values for the relaxation times *τ*, stretching parameter *β*, and *β*‐corrected average relaxation time 〈𝜏〉 obtained by fitting the data in Figure 3A.

	Disperse Si_∼16_‐C_11_			Discrete Si_16_‐C_11_		
Temperature (°C)	τ (s)	β (‐)	〈𝜏〉 (s)	τ (s)	β (‐)	〈𝜏〉 (s)
160	217	0.78	250	627	0.91	656
150	569	0.85	619	1546	0.84	1695
140	1276	0.81	1433	4486	0.80	5083
130	4224	0.78	4876	13721	0.74	16521

The differences in stress relaxation indicate a more hindered migration of dissociated ASCs in an ordered system compared to a disordered one. After dissociation, the hydrazide and isocyanate formed need to find new reaction partners for the network to undergo topological rearrangement. The ordered, aligned structure most likely makes it significantly more difficult for the hydrazide or isocyanate to diffuse out of the hydrogen‐bonded stack, leading to fewer successful exchange reactions, and therefore resulting in slower stress relaxation. Consequently, ordered structures add stability to DCNs. We exploit this property by looking at the high temperature creep behavior of the networks. Creep‐recovery measurements at 120°C and a stress of 0.5 MPa of both networks clearly show the effect of this increased stability, as Si_16_‐C_11_ creeps significantly less than Si_∼16_‐C_11_ (Figure 2D, <1800 s). Furthermore, during the recovery, Si_16_‐C_11_ shows nearly no permanent deformation (< 1%), whereas Si_∼16_‐C_11_ has a residual strain of around 8% (Figure 2D, >1800 s). The reduced creep in the ordered networks is again caused by more difficult topological rearrangement of the ASCs in an ordered environment compared to a disordered one. Embedding DCBs into highly ordered structures is therefore a unique way to enhance creep resistance to DCNs, which remains one of the main challenges of DCNs [42].

Highly ordered, aligned structures have interesting thermal properties that make them useful for advanced materials such as actuators for soft robotics [43]. These properties originate from anisotropic thermal expansion of the aligned materials [44]. Since actuating materials often require additional alignment steps during processing or device fabrication, a material such as Si_16_‐C_11_ that spontaneously aligns is of high interest as it eliminates the need for an additional processing step. We therefore investigate the potential of discrete Si_16_‐C_11_ networks to produce proof‐of‐concept thermo‐responsive actuators without an external alignment step.

As expected for homeotropically aligned materials, Si_16_‐C_11_ exhibits a small degree of bending upon heating. The thermo‐responsive effect can be significantly amplified by applying the discrete Si_16_‐C_11_ network to a nonaligned substrate that shows isotropic thermal expansion. For this purpose, we make use of the disperse Si_∼16_‐C_11_ network (dyed orange for visual clarity), as the dynamic covalent exchange between the ASCs in the ordered and disordered networks enables covalently bonding between the two films. Flower‐shaped specimens were cut from both materials and laminated by lightly pressing them together at 160°C for 30 min (Figure 3). Upon cooling and removing the applied load, contraction of the bilayer flower structure was observed. This contraction occurs in the direction of the ordered network film, and resembles the actuation behavior reported in LCNs [44]. When placed on a hotplate at 130°C, the flower opens up again (Video S1). This actuation is fully reversibly, and can be repeated over tens of cycles without noticeable fatigue. This proof‐of‐concept demonstration highlights the simplicity of fabricating thermo‐responsive actuators using Si_16_‐C_11_‐based DCNs. Furthermore, favorable mechanical properties and thermal stability make these materials promising candidates for demanding applications, including soft robotics [43, 44].

**FIGURE 3:**
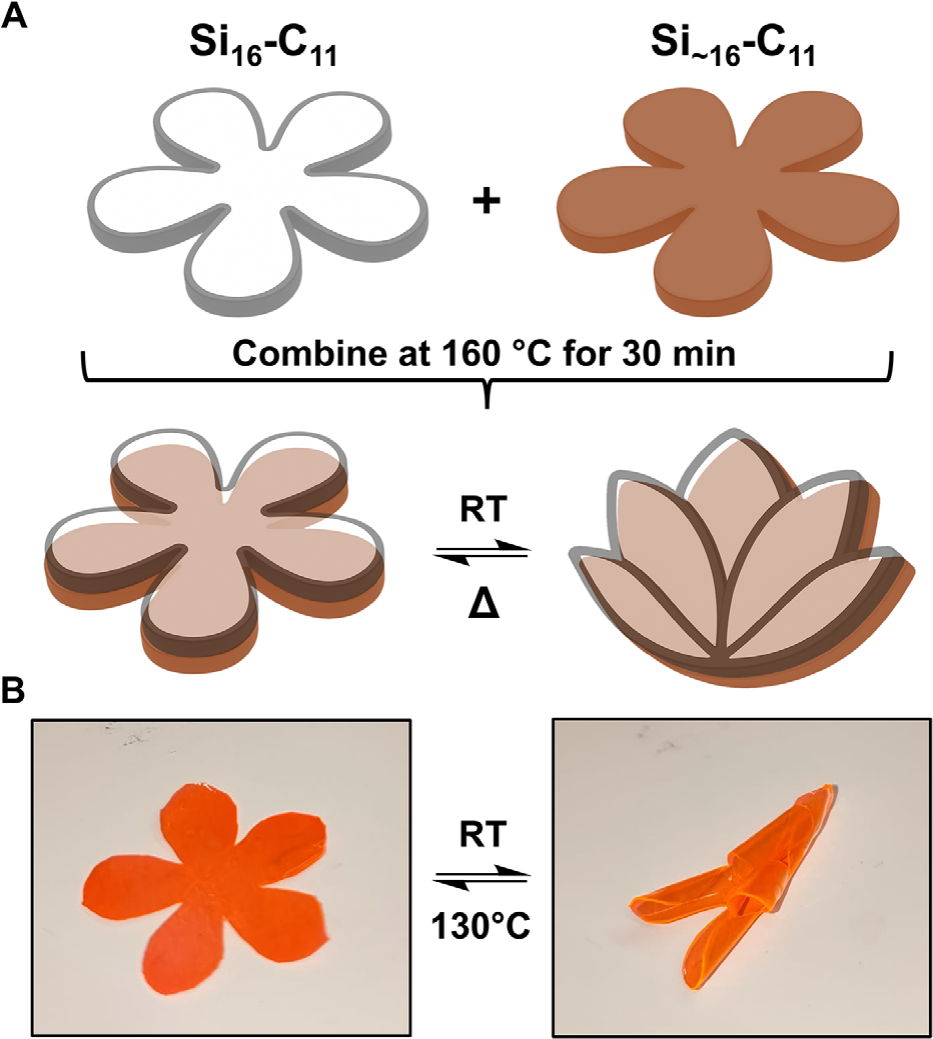
Schematic representation (A) and photographs (B) of an actuator flower that is made by combining ordered and disordered networks. The flower curls up at room temperature and opens at elevated temperatures.

## 3 Conclusion

DCNs with long‐range ordering and spontaneous alignment are easily prepared by combining discrete siloxanes and ASC motifs. By varying both the siloxane and alkyl spacer length, an optimum combination was found in discrete Si_16_‐C_11_ networks, which showed highly ordered hexagonal columnar domains. The lack of molar mass dispersity of the siloxane linker was the crucial factor in obtaining highly ordered structures, as a network with similar spacer length but disperse siloxane showed only disordered phase separation. This highlights the importance of controlling the dispersity of the prepolymers to obtain ordered structures in these networks, which could offer a more generalized strategy to obtain ordered DCNs. The nanostructure observed in the discrete Si_16_‐C_11_ networks is extraordinary considering that the long‐range ordering and homeotropic alignment occur spontaneously during the solvent evaporation and curing of the network film, which is in contrast to other ordered materials that often need an alignment step or layer. We further explored the unique properties of ordered structures in DCNs, which are characterized by higher moduli, slower stress relaxation, and improved creep resistance. Finally, we showed the straightforwardness of using these ordered networks to prepare actuators, without needing any alignment step.

## Author Contributions

**Stefan J. D. Maessen**: writing – original draft, writing – review & editing, formal analysis, data curation, conceptualization, investigation, validation, methodology, visualization. **Tymen M. L. van der Leede**: investigation, data curation. **Anne B. Spoelstra**: data curation, investigation, methodology. **Bart W. L. van den Bersselaar**: investigation, data curation. **Bas F. M. de Waal**: investigation, methodology. **Johan P. A. Heuts**: conceptualization, writing – review & editing, supervision. **Anja R. A. Palmans**: conceptualization, funding acquisition, supervision, writing – review & editing, project administration.

## Conflicts of Interest

The authors declare no conflicts of interest.

## Supporting information




**Supporting File 1**: anie73478‐sup‐0001‐SupMat.pdf.

**Supporting File 2**: anie73478‐sup‐0002‐VideoS1.mp4.

## Data Availability

The data that support the findings of this study are available from the corresponding author upon reasonable request.
