# Supplementary material for: Spontaneous Long‐Range Order and Alignment in Phase‐Separated Dynamic Covalent Networks Using Discrete Siloxanes
Source: Angew Chem Int Ed Engl. 2026 Jul 31;65(40):e5399898. doi: 10.1002/anie.5399898 (PMC13618305; doi:10.1002/anie.5399898)
Supplement: Supplementary file 1 [file ANIE-65-e5399898-s002.pdf]

Supporting Information

Spontaneous Long-Range Order and Alignment in Phase-Separated Dynamic Covalent Networks using Discrete Siloxanes

Stefan J.D. Maessen,[a] Tymen M.L. van der Leede,[a] Anne B. Spoelstra,[b] Bart W.L. van den Bersselaar,[a] Bas F.M. de Waal,[a] Johan P.A. Heuts,[a] Anja R.A. Palmans*[a]

a. Department of Chemical Engineering & Chemistry and Institute for Complex Molecular Systems, Eindhoven University of Technology, P.O. Box 513, 5600 MB Eindhoven, The Netherlands.

b. Department of Chemical Engineering & Chemistry and Center for Multiscale Electron Microscopy, Eindhoven University of Technology, P.O. Box 513, 5600 MB Eindhoven, The Netherlands.

*corresponding author:

a.palmans@tue.nl

Contents

1. Experimental section 3

1.1 Materials and Methods 3

Materials 3

General Methods 3

NMR spectroscopy 3

Matrix-assisted laser desorption/ionization time-of-flight 3

Fourier transform infrared spectroscopy 3

Size Exclusion Chromatography (SEC) 3

Gel fraction and swelling ratio 3

Thermogravimetric analysis (TGA) 4

Differential scanning calorimetry (DSC) 4

Medium- and wide-angle x-ray scattering (MAXS and WAXS) 4

Variable temperature infrared spectroscopy (VTIR) 5

Transmission Electron Microscopy (TEM) 5

Dynamic mechanical thermal analysis (DMTA) 5

Tensile tests 5

Stress relaxation measurements 5

Creep-recovery measurements 6

1.2 Synthetic procedures 6

Casting procedure 9

2. Supporting figures and tables 11

3. Characterization data 23

3.1 SEC 23

3.2 Gel fraction and swelling ratio 23

3.3 TGA and DSC 23

3.4 NMR and MALDI-ToF-MS 25

References 48

1. Experimental section

1.1 Materials and Methods

Materials

Unless stated otherwise, all reagents and chemicals were obtained from commercial sources at the highest purity available and used without further purification. Polydimethylsiloxane, hydride terminated, viscosity 7-10 cSt was obtained from ABCR. Discrete siloxanes were synthesized by Bas de Waal according to literature procedures.[1] All solvents were obtained from Biosolve except for acetonitrile which was obtained from Actu-All Chemicals. Dry solvents were obtained from an MBraun solvent purification system (MB SPS-800). Dry unstabilized THF from the solvent purification system was stored under argon and further dried with activated 4 Å molecular sieves, and filtered through a Whatman 2 μm PTFE syringe filter before use. Deuterated chloroform ($CDCl_3$) was purchased from Deutero GmbH and kept dry with 4 Å molecular sieves.

General Methods

NMR spectroscopy

^{1}H, ^{13}C NMR spectra were recorded on a 400 MHz Bruker Avance III Spectrometer (^{1}H using 400 MHz, ^{13}C using 100 MHz). Peak multiplicities are abbreviated as s: singlet, d: doublet, t: triplet, q: quartet, p: pentet, m: multiplet, dd: doublet of doublets, td: triplet of doublets, tt: triplet of triplets. Proton and carbon chemical shifts are reported in parts per million (ppm) downfield from tetramethylsilane (TMS) using the deuterated solvent resonance frequency as internal standard (7.26 ppm for $CDCl_3$).

Matrix-assisted laser desorption/ionization time-of-flight

Matrix-assisted laser desorption/ionization time-of-flight followed by mass spectrometry (MALDI-ToF-MS) mass analysis measurements were performed on a Bruker Autoflex Speed using α-cyano-4-hydroxycinnamic acid (CHCA) and *trans*-2-[3-(4-tert-butylphenyl)-2-methyl-2-propenylidene]malononitrile (DCTB) as matrices. Samples for MALDI-ToF-MS were dissolved with a concentration of 1 mg mL^{-1} in THF.

Fourier transform infrared spectroscopy

Fourier transform infrared spectroscopy (FTIR) spectra were recorded on a Shimadzu IRTracer-100 spectrometer using the MIRacle 10 single reflection ATR accessory.

Size Exclusion Chromatography (SEC)

SEC was performed on Varian/Polymer Laboratories PL-SEC 50 equipment equipped with refractive index (RI) and UV (254 nm) detectors. The system was equipped with a combination of Agilent PLgel 5-μm mixed-C column and Agilent PLgel 5-μm mixed-D column. The mobile phase was THF with BHT stabilizer and run at a flow rate of 1 mL min^{-1} at 40 °C. Polystyrene was used as calibration standard.

Gel fraction and swelling ratio

Gel fractions and swelling ratios of the network were determined in THF and demineralized water. A piece of the film (± 20 mg) was cut and weighed ($m_{initial}$) in a vial. The extraction solvent (± 8 mL) was added and the sample was left for 3 days. The solvent was removed with a needle and the sample was weighed ($m_{swollen}$). Next, the sample was dried in the oven at 50

°C in a vacuum for 4 hours. The dried sample was weighed (m_{dried}). The gel fractions and swelling ratios were calculated according to eqn(S1-S2).

$$\text{Gel fraction (\%)} = \frac{m_{\text{dried}}}{m_{\text{initial}}} \times 100\ \% \qquad (S1)$$

$$\text{Swelling ratio} = \frac{m_{\text{swollen}}}{m_{\text{initial}}} \qquad (S2)$$

Thermogravimetric analysis (TGA)

The thermal stabilities of the pDMS-ASC materials were determined using a TGA 550 (TA instruments) under N_2 flow. Temperature ramps were performed by ramping from 20 °C to 40 °C at 20 °C min^{-1}, keeping the sample isothermal at 40 °C for 5 minutes, followed by a ramp to 800 °C at 10 °C min^{-1}. Temperature calibration was performed using the Curie points of high purity ferromagnetic standards.

Differential scanning calorimetry (DSC)

DSC was performed in a Q2000 DSC (TA instruments) calibrated with an indium standard. Measurements were performed using standard aluminum pans between -40 °C and 160 °C. Heating and cooling runs were performed at 10 °C min^{-1}. The first cooling and second heating runs were reported.

Medium- and wide-angle x-ray scattering (MAXS and WAXS)

Bulk small-angle X-ray scattering (SAXS) was performed on a Ganesha instrument from SAXSLab. The flight tube and sample holder are all under vacuum in a single housing, with a GeniX-Cu ultra-low divergence X-ray generator. The source produces X-rays with a wavelength (λ) of 0.154 nm and flux of 1 × 108 ph s^{-1}. Scattered X-rays were captured on a 2-dimensional Pilatus 300K detector with 487 × 619 pixel resolution. The sample-to-detector distance was 0.084 m (WAXS mode) or 0.48 m (MAXS mode). The instrument was calibrated with diffraction patterns from silver behenate.

The domain spacing *d* (in nm) was calculated from the principle scattering peak *q* using eqn(S3). In case of a hexagonal columnar phase, this domain spacing corresponds to d_{hex}.

$$d = \frac{2\pi}{q} \qquad (S3)$$

The hexagonal lattice parameter a was calculated from the domain spacing from the principle scattering peak d_{hex} following eqn(S4).

$$a = \frac{2d_{he}}{\sqrt{3}} \qquad (S4)$$

An estimate of the Scherrer-type correlation length ξ was made from the full width at half maximum (FWHM) of the principle scattering peak Δq using eqn(S5). Note that this estimate does not account for instrumental broadening or anisotropy effects among others, meaning this estimate falls lower than the actual correlation length.

$$\xi \approx \frac{2\pi}{\Delta q} \qquad (S5)$$

Grazing Incidence Medium/Wide Angle X-ray Scattering (GIMAXS/WAXS) was performed at the critical angle (0.2°) on free-standing films.

Variable temperature infrared spectroscopy (VTIR)

VTIR spectra were obtained at the Bruker Tensor 27. A sample was heated to 200 °C and cooled back to 25 °C, and this was repeated once, both with a heating rate of 3 °C/min. At 200 °C, the sample was first equilibrated for 5 min before cooling. IR spectra were collected every 5 °C.

Transmission Electron Microscopy (TEM)

Cryo ultramicrotomy was performed using a Leica UC7/FC7 ultramicrotome with a Diatome© 35° diamond knife. During microtoming the sample was held at -130°C and the diamond knife at -45 °C. The knife has a trough with a 50/50 H_2O/DMSO mixture for floating off the ultrathin sections.

The top side of the ~100 μm thick film was positioned parallel to the knife edge and the sections were taken approximately 20 μm into the film. The 50 nm ultrathin sections were cut parallel to the surface of the film and transferred to a continuous carbon coated 200 mesh Cu grid.

TEM imaging was performed on a TITAN (Thermo Fisher, previously FEI), equipped with a field emission gun (FEG) and images were captured using the 4k × 4k CETA II 16M CMOS camera with 4096×4096 pixels. The microscope was operated at 300 kV acceleration voltage in bright-field TEM mode.

Dynamic mechanical thermal analysis (DMTA)

DMTA was performed on rectangular shaped samples using the film tension setup in a Discovery DMA 850 (TA instruments). Samples were cooled to -150 °C and left to equilibrate for 2 hours. Then, a temperature ramp was performed at 3 °C min^{-1} from -150 °C to 200 °C. A preload force of 0.1 N, strain of 1 % and a force track of 125% was used. The storage and loss modulus were recorded as a function of temperature.

Tensile tests

Tensile tests were performed on rectangular shaped samples using the film tension setup in a Discovery DMA 850 (TA instruments). A sample with an initial length of 5.0 mm ± 0.2 mm and a width of 5.3 mm was clamped in the setup. A preload force of 0.1N was applied. A rate of 0.1 mm/min (~2%/min) was used to put the sample in tension. The stress-strain curves were obtained at room temperature.

Stress relaxation measurements

DMTA was performed on rectangular shaped samples using the film tension setup in a Discovery DMA 850 (TA instruments). The sample was equilibrated for 3 min at the temperature before the start of the experiment. The stress relaxations were performed with a strain of 5.0 % on the same sample. The normalization of the data was done by fitting a straight line through the noise and normalizing to the value of the fit at 1 s.

The β-averaged relaxation time $\langle\tau\rangle$ has been calculated from eqn(S6), where τ and β are fitted according to eqn(1), and Γ is the gamma function.[2]

$$\langle\tau\rangle = \frac{\tau \cdot \Gamma\left(\frac{1}{\beta}\right)}{\beta} \qquad (S6)$$

Creep-recovery measurements

DMTA was performed on rectangular shaped samples using the film tension setup in a Discovery DMA 850 (TA instruments). Samples were equilibrated at the measurement temperature for 3 minutes. Creep measurements were performed at 120 °C. 0.5 MPa of tensile stress was applied to the sample for 1800 seconds, with a recovery period of 1800 seconds.

1.2 Synthetic procedures

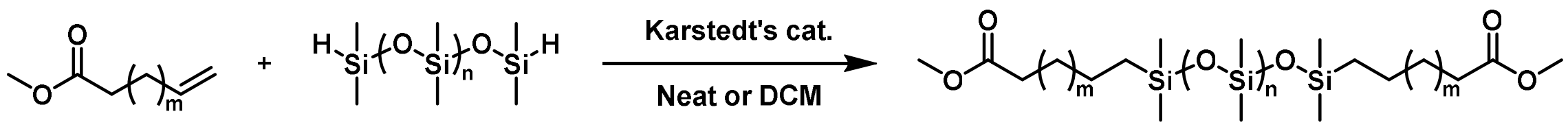

Synthesis of methyl ester-terminated discrete pDMS: *General procedure:* Discrete polydimethylsiloxane, hydride terminated (1 eq) was mixed with methyl undec-10-enoate or methyl hex-5-enoate (~2.4 eq) and DCM in a dried 50 mL round-bottom flask under argon atmosphere. The mixture was cooled with an ice bath, and Karstedt's catalyst (2 wt% in xylene) was added (2 drops), after which the mixture was stirred for several hours to days until the Si-H peak (δ = 4.70 ppm in $CDCl_3$) on ^{1}H NMR fully disappeared. The excess methyl ester was removed in *vacuo* (~0.3 mbar) at 100 °C. The polymer oil was dissolved in DCM and filtered over a plug of activated charcoal in a glass filter to remove the platinum catalyst. The charcoal plug was washed 2x with DCM and the solvent was removed in *vacuo* to yield the pure product.

Si_8-C_6 dimethyl ester: Discrete pDMS H-Si_8-H (2.07 g, 3.57 mmol, 1 eq), methyl 5-hexenoate (1.61 g, 8.1 mmol, 2.2 eq), no DCM. Product obtained as a colorless oil (2.89 g, 97%). ^{1}H NMR (400 MHz, $CDCl_3$) δ = 3.66 (s, 6H), 2.30 (t, J = 7.6 Hz, 4H), 1.62 (p, J = 7.8, 7.4 Hz, 4H), 1.42 – 1.23 (m, 8H), 0.62 – 0.40 (m, 4H), 0.25 – -0.15 (m, 48H). ^{13}C NMR (100 MHz, $CDCl_3$) δ = 174.46, 51.57, 34.21, 33.01, 24.84, 23.04, 18.22, 1.32, 1.22, 0.31. MALDI-ToF-MS: m/z calc. for $C_{30}H_{74}O_{11}Si_8$: 834.34 Da; found 857.42 Da $[M+Na]^+$.

Si_8-C_{11} dimethyl ester: Discrete pDMS H-Si_8-H (2.14 g, 3.7 mmol, 1 eq), methyl 10-undecenoate (1.61 g, 8.1 mmol, 2.2 eq), no DCM. Product obtained as a colorless oil (3.52 g, 98%). ^{1}H NMR (400 MHz, $CDCl_3$) δ = 3.66 (s, 6H), 2.30 (t, J = 7.6 Hz, 4H), 1.61 (p, J = 7.2 Hz, 4H), 1.38 – 1.16 (m, 28H), 0.60 – 0.44 (m, 4H), 0.11 – 0.02 (m, 48H). ^{13}C NMR (100 MHz, $CDCl_3$) δ = 174.49, 51.57, 34.28, 33.60, 29.69, 29.65, 29.53, 29.42, 29.33, 25.13, 23.38, 18.42, 1.33, 1.22, 0.34. MALDI-ToF-MS: m/z calc. for $C_{40}H_{94}O_{11}Si_8$: 974.50 Da; found 997.39 Da $[M+Na]^+$.

Si_{16}-C_6 dimethyl ester: Discrete pDMS H-Si_{16}-H (3.05 g, 2.6 mmol, 1 eq), methyl 5-hexenoate (860 mg, 6.7 mmol, 2.6 eq), no DCM. Product obtained as a colorless oil (3.59 g, 97%). ^{1}H NMR (400 MHz, $CDCl_3$) δ = 3.66 (s, 6H), 2.30 (t, J = 7.6 Hz, 4H), 1.62 (p, J = 7.4 Hz, 4H), 1.41 – 1.23 (m, 8H), 0.60 – 0.44 (m, 4H), 0.30 – -0.16 (m, 96H). ^{13}C NMR (100 MHz, $CDCl_3$) δ = 174.46, 51.57, 34.20, 33.00, 24.83, 23.04, 18.21, 1.31, 1.21, 1.20, 0.30. MALDI-ToF-MS: m/z calc. for $C_{46}H_{122}O_{19}Si_{16}$: 1426.49 Da; found 1449.69 Da $[M+Na]^+$.

Si_{16}-C_{11} dimethyl ester: Discrete pDMS H-Si_{16}-H (3.10 g, 2.7 mmol, 1 eq), methyl 10-undecenoate (1.33 g, 6.7 mmol, 2.5 eq), no DCM. Product obtained as a colorless oil (3.79 g, 91%). ^{1}H NMR (400 MHz, $CDCl_3$) δ = 3.66 (s, 6H), 2.30 (t, J = 7.6 Hz, 4H), 1.61 (q, J = 7.3 Hz, 4H), 1.37 – 1.18 (m, 28H), 0.60 – 0.44 (m, 4H), 0.14 – 0.01 (m, 96H). ^{13}C NMR (100 MHz, $CDCl_3$) δ = 174.50, 51.57, 34.28, 33.60, 29.69, 29.65, 29.52, 29.42, 29.32, 25.12, 23.37, 18.42,

1.32, 1.20, 0.33. MALDI-ToF-MS: m/z calc. for $C_{56}H_{142}O_{19}Si_{16}$: 1566.65 Da; found 1589.60 Da $[M+Na]^+$.

Si_{24}-C_{11} dimethyl ester: Discrete pDMS H-Si_{24}-H (441 mg, 0.25 mmol, 1 eq), methyl 10-undecenoate (125 mg, 0.63 mmol, 2.5 eq), 1 mL DCM. Product obtained as a colorless oil (510 mg, 96%). ^{1}H NMR (400 MHz, $CDCl_3$) δ = 3.66 (s, 6H), 2.29 (t, J = 7.6 Hz, 4H), 1.67 – 1.58 (m, 4H), 1.39 – 1.16 (m, 28H), 0.60 – 0.44 (m, 4H), 0.14 – 0.01 (m, 144H). ^{13}C NMR (100 MHz, $CDCl_3$) δ = 174.51, 51.58, 34.28, 33.60, 29.69, 29.65, 29.52, 29.42, 29.32, 25.12, 23.37, 18.42, 1.32, 1.19, 0.34. MALDI-ToF-MS: m/z calc. for $C_{72}H_{190}O_{27}Si_{24}$: 2158.80 Da; found 2183.58 Da $[M+Na]^+$.

Si_{32}-C_{11} dimethyl ester: Discrete pDMS H-Si_{32}-H (307 mg, 0.13 mmol, 1 eq), methyl 10-undecenoate (62 mg, 0.31 mmol, 2.4 eq), 1 mL DCM. Product obtained as a colorless oil (348 mg, 97%). ^{1}H NMR (400 MHz, $CDCl_3$) δ = 3.66 (s, 6H), 2.30 (t, J = 7.6 Hz, 4H), 1.62 (t, J = 7.3 Hz, 4H), 1.40 – 1.16 (m, 29H), 0.60 – 0.46 (m, 4H), 0.17 – 0.01 (m, 192H). ^{13}C NMR (100 MHz, $CDCl_3$) δ = 174.50, 51.57, 34.27, 33.60, 29.69, 29.64, 29.52, 29.42, 29.32, 25.12, 23.37, 18.41, 1.31, 1.18. MALDI-ToF-MS: m/z calc. for $C_{88}H_{238}O_{35}Si_{32}$: 2750.95 Da; found 2723.63 Da $[M+Na]^+$.

Si_{40}-C_{11} dimethyl ester: Discrete pDMS H-Si_{40}-H (300 mg, 0.10 mmol, 1 eq), methyl 10-undecenoate (52 mg, 0.26 mmol, 2.6 eq), 1 mL DCM. Product obtained as a colorless oil (340 mg, 99%). ^{1}H NMR (400 MHz, $CDCl_3$) δ = 3.66 (s, 6H), 2.30 (t, J = 7.6 Hz, 4H), 1.69 – 1.57 (m, 4H), 1.38 – 1.16 (m, 29H), 0.58 – 0.46 (m, 4H), 0.09 – 0.03 (m, 240H). ^{13}C NMR (100 MHz, $CDCl_3$) δ = 174.50, 51.57, 34.28, 33.60, 29.69, 29.65, 29.52, 29.42, 29.32, 25.12, 23.37, 18.42, 1.32, 1.18, 0.33. MALDI-ToF-MS: m/z calc. for $C_{104}H_{286}O_{43}Si_{40}$: 3343.10 Da; found 3366.66 Da $[M+Na]^+$.

Synthesis of methyl ester-terminated disperse pDMS (C_{11}-$Si_{\sim16}$): Disperse polydimethylsiloxane, hydride terminated (7-10 cSt) (30 g, 1 eq) was mixed with methyl undec-10-enoate (11.93 g, 2.5 eq, 60.16 mmol) in a dried 100 mL round-bottom flask under argon atmosphere. The mixture was cooled with an ice bath, and Karstedt's catalyst (2 wt% in xylene) was added (4 drops), after which the mixture was stirred for 16 hours until the Si-H peak (δ = 4.70 ppm in $CDCl_3$) on ^{1}H NMR fully disappeared. The mixture was transferred to a separatory funnel and extracted with 3 x 50 mL of acetonitrile to remove excess methyl undec-10-enoate. After this, the polymer oil was dissolved in 50 mL of DCM and filtered over a plug of activated charcoal in a glass filter to remove the platinum catalyst. The charcoal plug was washed with 2x 50 mL of DCM. The solvent was removed in *vacuo* after which the product was obtained as a colorless oil (38.3 g, 97%). ^{1}H NMR (400 MHz, $CDCl_3$) δ = 3.66 (s, 6H), 2.30 (t, J = 7.5 Hz, 4H), 1.61 (q, J = 7.2 Hz, 4H), 1.43 – 1.11 (m, 29H), 0.62 – 0.44 (m, 4H), 0.28 – -0.15 (m, 98H). ^{13}C NMR (100 MHz, $CDCl_3$) δ = 174.43, 51.52, 34.26, 33.60, 29.69, 29.65, 29.52, 29.42, 29.32, 25.12, 23.38, 18.42, 1.31, 1.20, 1.18. NMR M_n: 1.60 kDa.

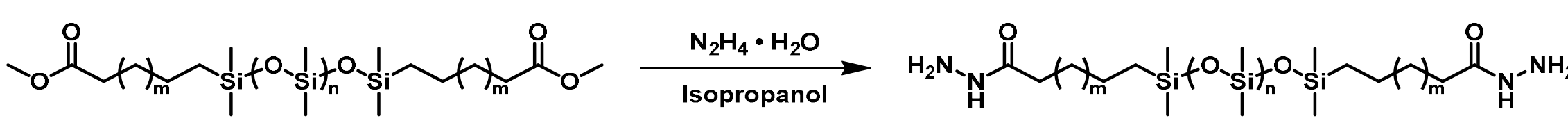

Synthesis of hydrazide-terminated discrete pDMS: *General procedure:* Methyl ester-terminated discrete pDMS (1 eq) was dissolved in isopropanol in a 50 mL round-bottom flask equipped with an oil bath and reflux condenser under argon. Hydrazine monohydrate was added to the solution, after which the mixture was left to stir at 50 °C until the O-CH_3 peak (δ = 3.66 ppm in $CDCl_3$) on ^{1}H NMR fully disappeared. The isopropanol was removed in *vacuo*, and the mixture was dissolved in DCM. The mixture was transferred to a separatory funnel and extracted 3x with demineralized water to remove excess hydrazine. After this, the DCM layer

was dried over Na_2SO_4, filtered using gravity and the filter washed with DCM. The solvent was removed in *vacuo* to yield the pure product.

Si_8-C_6 dihydrazide: Si_8-C_6 dimethyl ester (2.8 g, 3.35 mmol, 1 eq), 3 mL isopropanol, hydrazine monohydrate (3.0 g, 59.9 mmol, 18 eq). Product obtained as a colorless waxy solid (2.72 g, 97%). ^{1}H NMR (400 MHz, $CDCl_3$) δ = 7.09 (s, 2H), 3.91 (s, 3H), 2.13 (t, J = 7.8 Hz, 4H), 1.62 (p, J = 7.4 Hz, 4H), 1.42 – 1.19 (m, 8H), 0.64 – 0.40 (m, 4H), 0.27 – -0.22 (m, 48H). ^{13}C NMR (100 MHz, $CDCl_3$) δ = 174.20, 34.62, 33.10, 25.34, 23.05, 18.19, 1.29, 1.18, 0.27. MALDI-ToF-MS: m/z calc. for $C_{30}H_{74}N_4O_{11}Si_8$: 834.36 Da; found 857.46 Da $[M+Na]^+$.

Si_8-C_{11} dihydrazide: Si_8-C_{11} dimethyl ester (3.45 g, 3.5 mmol, 1 eq), 3 mL isopropanol, hydrazine monohydrate (3.0 g, 59.9 mmol, 17 eq). Product obtained as a colorless waxy solid (3.18 g, 92%). ^{1}H NMR (400 MHz, $CDCl_3$) δ = 6.92 (s, 2H), 3.91 (s, 4H), 2.14 (t, J = 7.8 Hz, 4H), 1.62 (p, J = 7.3 Hz, 4H), 1.39 – 1.13 (m, 28H), 0.61 – 0.40 (m, 4H), 0.14 – -0.00 (m, 48H). ^{13}C NMR (100 MHz, $CDCl_3$) δ = 174.20, 34.73, 33.58, 29.69, 29.65, 29.51, 29.45, 29.44, 25.66, 23.35, 18.40, 1.31, 1.20, 0.32. MALDI-ToF-MS: m/z calc. for $C_{38}H_{94}N_4O_9Si_8$: 974.52 Da; found 997.45 Da $[M+Na]^+$.

Si_{16}-C_6 dihydrazide: Si_{16}-C_6 dimethyl ester (3.5 g, 2.5 mmol, 1 eq), 3 mL isopropanol, hydrazine monohydrate (1.8 g, 37 mmol, 16 eq). Product obtained as a colorless viscous liquid (3.34 g, 91%). ^{1}H NMR (400 MHz, $CDCl_3$) δ = 6.71 (s, 2H), 3.89 (s, 4H), 2.15 (t, J = 7.7 Hz, 4H), 1.63 (p, J = 7.5 Hz, 5H), 1.50 – 1.22 (m, 8H), 0.70 – 0.35 (m, 4H), 0.35 – -0.21 (m, 96H). ^{13}C NMR (100 MHz, $CDCl_3$) δ = 174.12, 34.69, 33.13, 25.36, 23.08, 18.23, 1.32, 1.22, 1.20, 0.30. MALDI-ToF-MS: m/z calc. for $C_{44}H_{122}N_4O_{17}Si_{16}$: 1426.51 Da; found 1451.81 Da $[M+Na]^+$.

Si_{16}-C_{11} dihydrazide: Si_{16}-C_{11} dimethyl ester (3.69 g, 2.4 mmol, 1 eq), 3 mL isopropanol, hydrazine monohydrate (1.8 g, 37 mmol, 16 eq). Product obtained as a colorless oil (3.34 g, 91%). ^{1}H NMR (400 MHz, $CDCl_3$) δ = 6.68 (s, 1H), 3.89 (s, 2H), 2.14 (t, J = 7.9, 7.4 Hz, 4H), 1.62 (q, J = 7.4 Hz, 4H), 1.38 – 1.16 (m, 29H), 0.59 – 0.46 (m, 4H), 0.14 – 0.01 (m, 96H). ^{13}C NMR (100 MHz, $CDCl_3$) δ = 174.14, 34.76, 33.60, 29.70, 29.66, 29.52, 29.46, 25.66, 23.37, 18.42, 1.32, 1.20, 0.34. MALDI-ToF-MS: m/z calc. for $C_{54}H_{142}N_4O_{17}Si_{16}$: 1566.67 Da; found 1589.63 Da $[M+Na]^+$.

Si_{24}-C_{11} dihydrazide: Si_{24}-C_{11} dimethyl ester (480 mg, 0.22 mmol, 1 eq), 1 mL isopropanol, hydrazine monohydrate (1.0 g, 20.0 mmol, 90 eq). Product obtained as a colorless oil (456 mg, 95%). ^{1}H NMR (400 MHz, $CDCl_3$) δ = 6.66 (s, 2H), 3.88 (s, 4H), 2.14 (t, J = 6.8 Hz, 4H), 1.62 (q, J = 7.3 Hz, 4H), 1.38 – 1.14 (m, 28H), 0.62 – 0.44 (m, 4H), 0.17 – 0.02 (m, 144H). ^{13}C NMR (100 MHz, $CDCl_3$) δ = 174.15, 34.76, 33.61, 29.70, 29.66, 29.53, 29.47, 25.66, 23.38, 18.42, 1.32, 1.19, 0.34. MALDI-ToF-MS: m/z calc. for $C_{70}H_{190}N_4O_{25}Si_{24}$: 2158.82 Da; found 2181.79 Da $[M+Na]^+$.

Si_{32}-C_{11} dihydrazide: Si_{32}-C_{11} dimethyl ester (320 mg, 0.12 mmol, 1 eq), 1 mL isopropanol, hydrazine monohydrate (1.0 g, 20.0 mmol, 172 eq). Product obtained as a colorless oil (320 mg, 97%). ^{1}H NMR (400 MHz, $CDCl_3$) δ = 6.65 (s, 2H), 3.88 (s, 4H), 2.14 (t, J = 6.7 Hz, 4H), 1.63 (t, J = 7.1 Hz, 4H), 1.41 – 1.12 (m, 28H), 0.58 – 0.46 (m, 4H), 0.18 – 0.02 (m, 192H). ^{13}C NMR (100 MHz, $CDCl_3$) δ = 174.15, 34.76, 33.60, 29.70, 29.66, 29.52, 29.46, 25.66, 23.37, 18.42, 1.32, 1.19, 0.33. MALDI-ToF-MS: m/z calc. for $C_{86}H_{238}N_4O_{33}Si_{32}$: 2750.97 Da; found 2773.87 Da $[M+Na]^+$.

Si_{40}-C_{11} dihydrazide: Si_{40}-C_{11} dimethyl ester (320 mg, 0.096 mmol, 1 eq), 1 mL isopropanol, hydrazine monohydrate (1.0 g, 20.0 mmol, 209 eq). Product obtained as a colorless oil (285 mg, 89%). ^{1}H NMR (400 MHz, $CDCl_3$) δ = 6.63 (s, 2H), 3.88 (s, 4H), 2.14 (t, J = 7.6 Hz, 4H), 1.63 (t, J = 7.3 Hz, 4H), 1.38 – 1.16 (m, 29H), 0.57 – 0.46 (m, 4H), 0.18 – 0.02 (m, 240H). ^{13}C NMR (100 MHz, $CDCl_3$) δ = 174.14, 34.76, 33.61, 29.70, 29.66, 29.53, 29.46, 25.66, 23.38,

18.42, 1.32, 1.19, 0.34. MALDI-ToF-MS: m/z calc. for $C_{102}H_{286}N_4O_{41}Si_{40}$: 3343.12 Da; found 3366.96 Da $[M+Na]^+$.

Synthesis of hydrazide-terminated disperse pDMS (C_{11}-$Si_{\sim 16}$): Methyl ester-terminated disperse pDMS (38.3 g, 1 eq) was dissolved in isopropanol in a 50 mL round-bottom flask equipped with an oil bath and reflux condenser under argon. Hydrazine monohydrate (9.0 g, 0.18 mol) was added to the solution, after which the mixture was left to stir at 50 °C until the O-CH_3 peak (δ = 3.66 ppm in $CDCl_3$) on ^{1}H NMR fully disappeared. The isopropanol was removed in *vacuo*, and the mixture was dissolved in 150 mL of DCM. The mixture was transferred to a separatory funnel and extracted with 3 x 150 mL of demineralized water to remove excess hydrazine. After this, the DCM layer was dried over Na_2SO_4, filtered using gravity and the filter washed with 50 mL of DCM. The solvent was removed in *vacuo* to yield the pure product (35 g, 91%). ^{1}H NMR (400 MHz, $CDCl_3$) δ = 6.83 (s, 2H), 3.90 (s, 4H), 2.14 (t, J = 7.6 Hz, 4H), 1.63 (p, J = 6.5, 5.9 Hz, 4H), 1.52 – 1.07 (m, 28H), 0.51 (t, J = 7.4 Hz, 4H), 0.28 – -0.18 (m, 95H). ^{13}C NMR (100 MHz, $CDCl_3$) δ = 174.24, 34.70, 33.57, 29.68, 29.64, 29.50, 29.45, 29.43, 25.66, 23.34, 18.38, 1.29, 1.17, 1.15. NMR M_n: 1.55 kDa. SEC (RI detector): M_n: 1.4 kDa, M_w: 1.8 kDa, PDI: 1.3.

Casting procedure

A petri dish or aluminium dish was cleaned using acetone and dried thoroughly. Two vials were filled with argon gas. The pDMS prepolymer was dissolved in THF in the first argon filled vial. The HDI-trimer was dissolved in the second argon filled vial. The vials were combined, and shaken thoroughly for 15 seconds, after which the contents were casted into the dish and placed on a hotplate set to 50 °C. Using a funnel attached to the argon line, the dish was covered by the funnel and left under minimal argon flow at 50 °C. After 1 hour, the film was removed from the dish, and put on a Teflon block in an oven for 1 hour at 160 °C to cure. The amounts of sample used are summarized in Table S1.

Table S1: Conditions for preparation of polymer network films.

Network	Dish diameter /type (cm)	Prepolymer mass (mg)	Amount THF vial 1 (mL)	HDI-trimer mass (mg)	Amount THF vial 2 (mL)
**Si_8-C_6**	4, glass	100	2	51	1
**Si_8-C_{11}**	8, aluminium	500	5	218	3
**Si_{16}-C_6**	4, glass	100	1	29.8	1
**Si_{16}-C_{11}**	8, aluminium	500	6	127	4
**Si_{24}-C_{11}**	3, glass	100	1	19.7	1
**Si_{32}-C_{11}**	3, glass	100	1	15.5	1
**Si_{40}-C_{11}**	3, glass	100	1	12.7	1
**$Si_{\sim 16}$-C_{11}**	8, aluminium	500	6	123	4

2. Supporting figures and tables

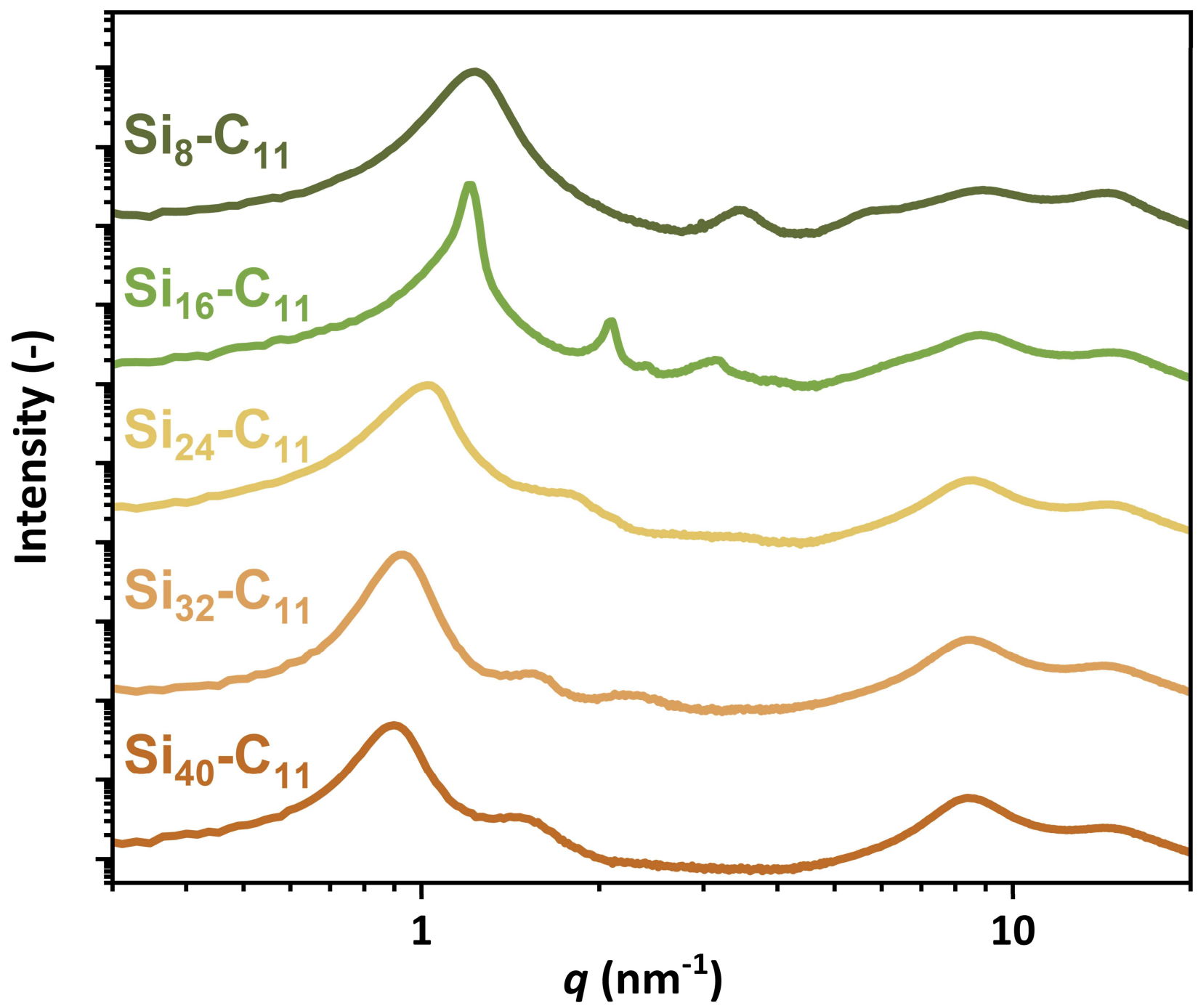

Figure S1: MAXS and WAXS 1D transmission scattering profiles of uncured Si_n-C_{11} networks.

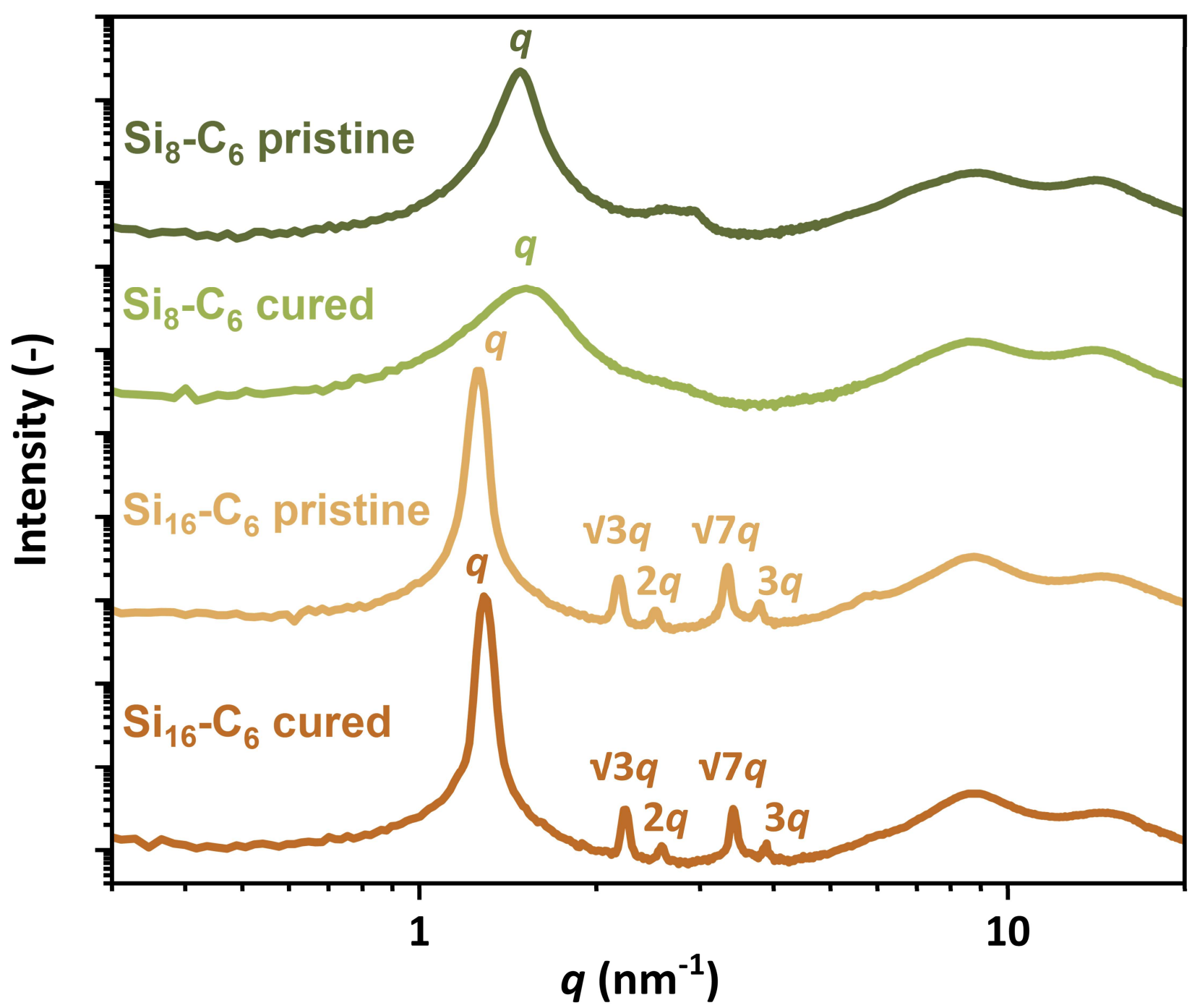

Figure S2: MAXS and WAXS 1D transmission scattering profiles of cured Si_n-C_6 networks.

Table S2: Miller indices, *q*-values, *q* / *q*(100) values, expected *q* / *q*(100) values for hexagonal columnar packing, domain spacings, lattice parameters, FWHM Δ*q* and estimated correlation lengths *ξ* for all polymer networks.

Network	hkl	q (nm^{-1})	q / q(100)	Expected q / q(100)	d (nm)	Lattice parameter (nm)	FWHM Δq (nm^{-1})	Estimated Correlation Length ξ (nm)
Si_8-C_6	-	1.54	-	-	4.08	-	0.4524	14
Si_8-C_{11}	-	1.31	-	-	4.79	-	0.2819	22
Si_{16}-C_6	(100)	1.29	1.00	1.00	4.87	a = 5.62	0.0755	83
	(110)	2.20	1.71	1.73	2.85			
	(200)	2.53	1.96	2.00	2.48			
	(210)	3.35	2.60	2.65	1.87			
	(300)	3.80	2.95	3.00	1.65			
Si_{16}-C_{11}	(100)	1.15	1.00	1.00	5.46	a = 6.30	0.0761	83
	(110)	1.97	1.71	1.73	3.19			
	(200)	2.29	1.99	2.00	2.74			
	(210)	3.01	2.62	2.65	2.09			
	(300)	3.42	2.97	3.00	1.84			
	(310)	3.94	3.43	3.46	1.59			
$Si_{\sim16}$-C_{11}	-	0.99	-	-	6.34	-	0.3233	19
Si_{24}-C_{11}	(100)	1.06	1.00	1.00	5.92	a = 6.84	0.1568	40
	(110)	1.81	1.71	1.73	3.47			
	(210)	2.77	2.61	2.65	2.27			
Si_{32}-C_{11}	(100)	0.99	1.00	1.00	6.34	a = 7.32	0.1725	36
	(110)	1.66	1.68	1.73	3.78			
	(210)	2.57	2.60	2.65	2.44			
Si_{40}-C_{11}	(100)	0.99	1.00	1.00	6.34	a = 7.32	0.1633	38

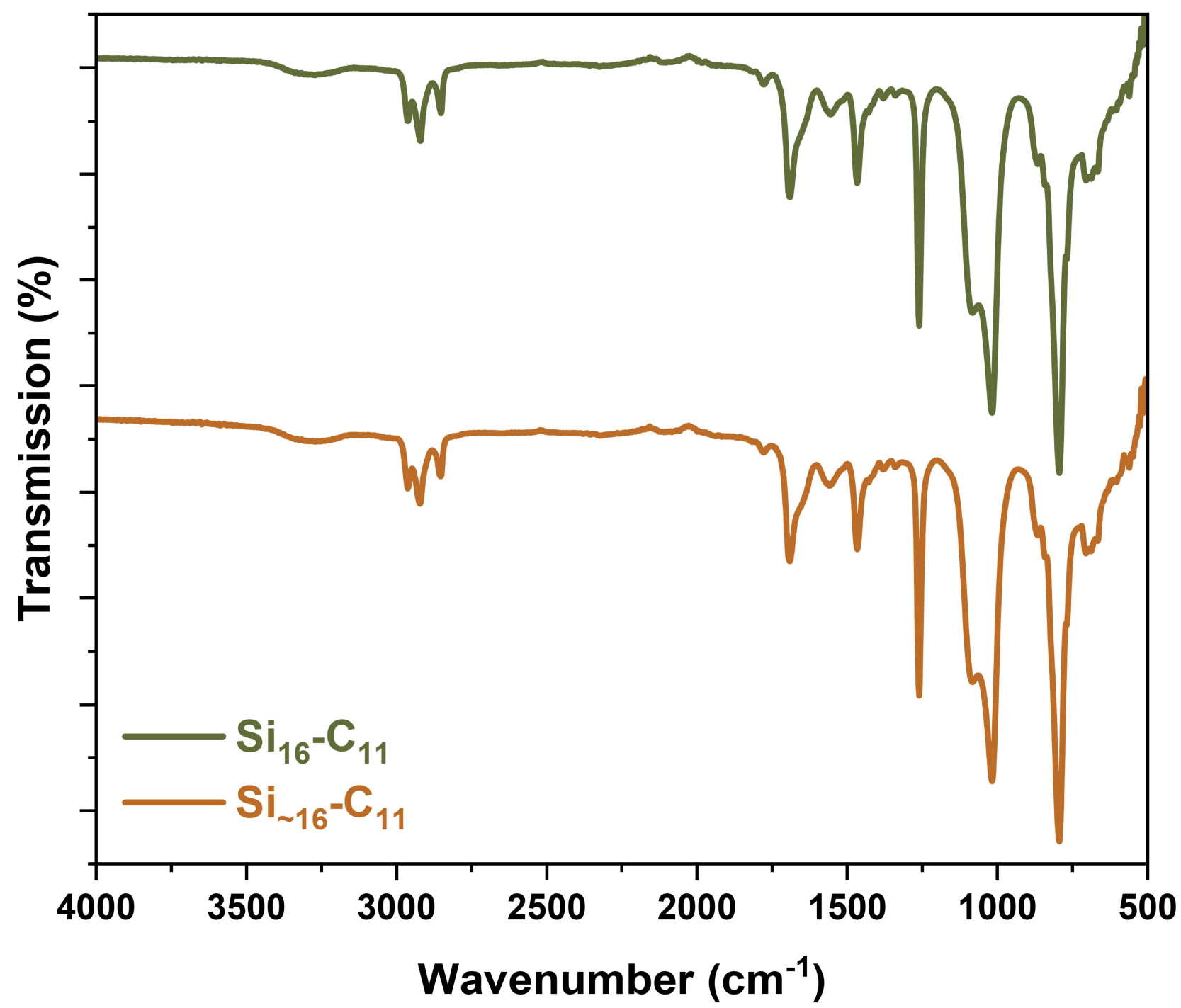

Figure S3: FTIR spectra of cured Si_{16}-C_{11} and $Si_{\sim 16}$-C_{11} networks.

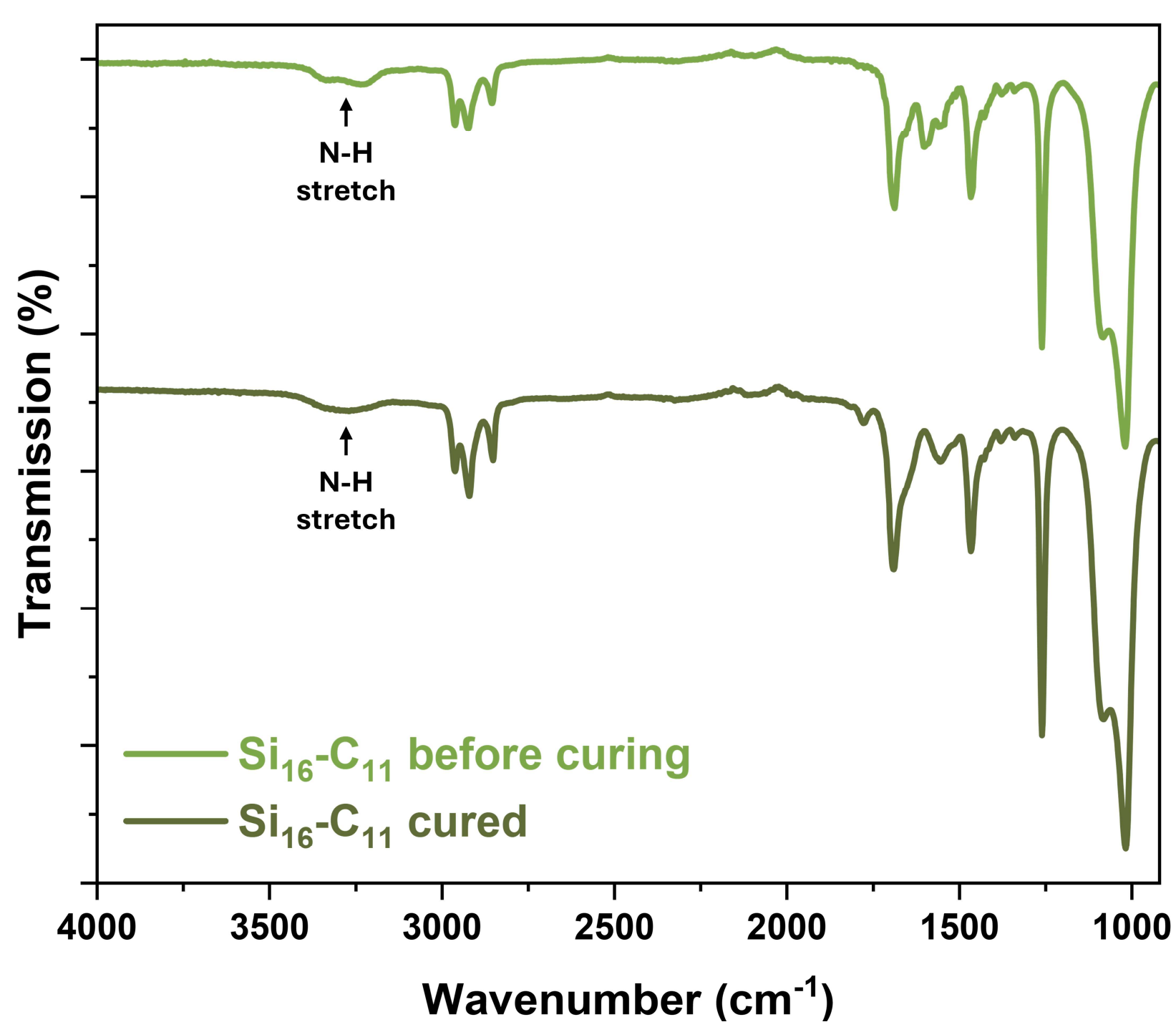

Figure S4: FTIR spectra of Si_{16}-C_{11} network before and after curing.

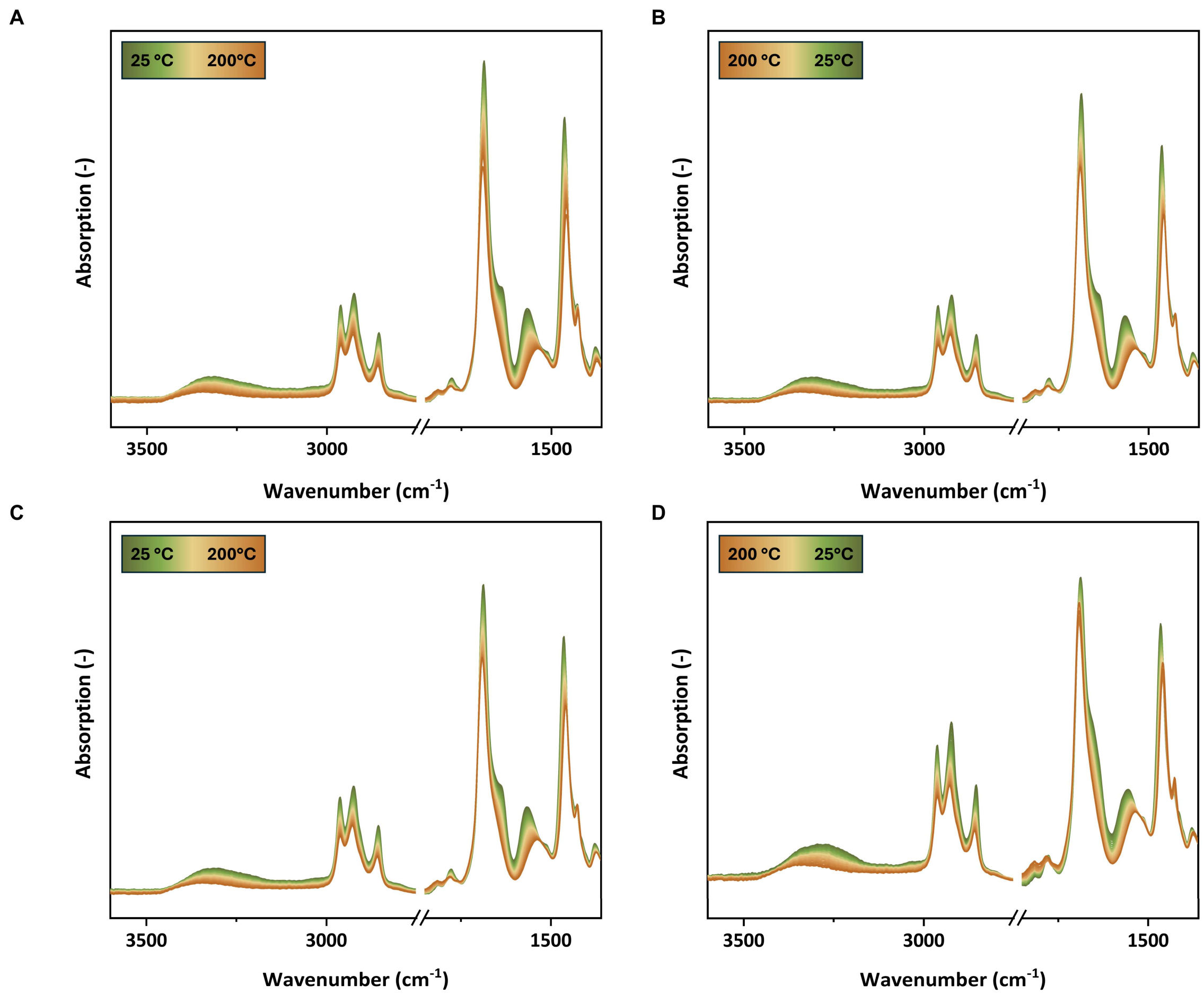

Figure S5: Variable temperature FTIR spectra of Si_{16}-C_{11} network after curing, during A) first heating, B) first cooling, C) second heating and D) second cooling.

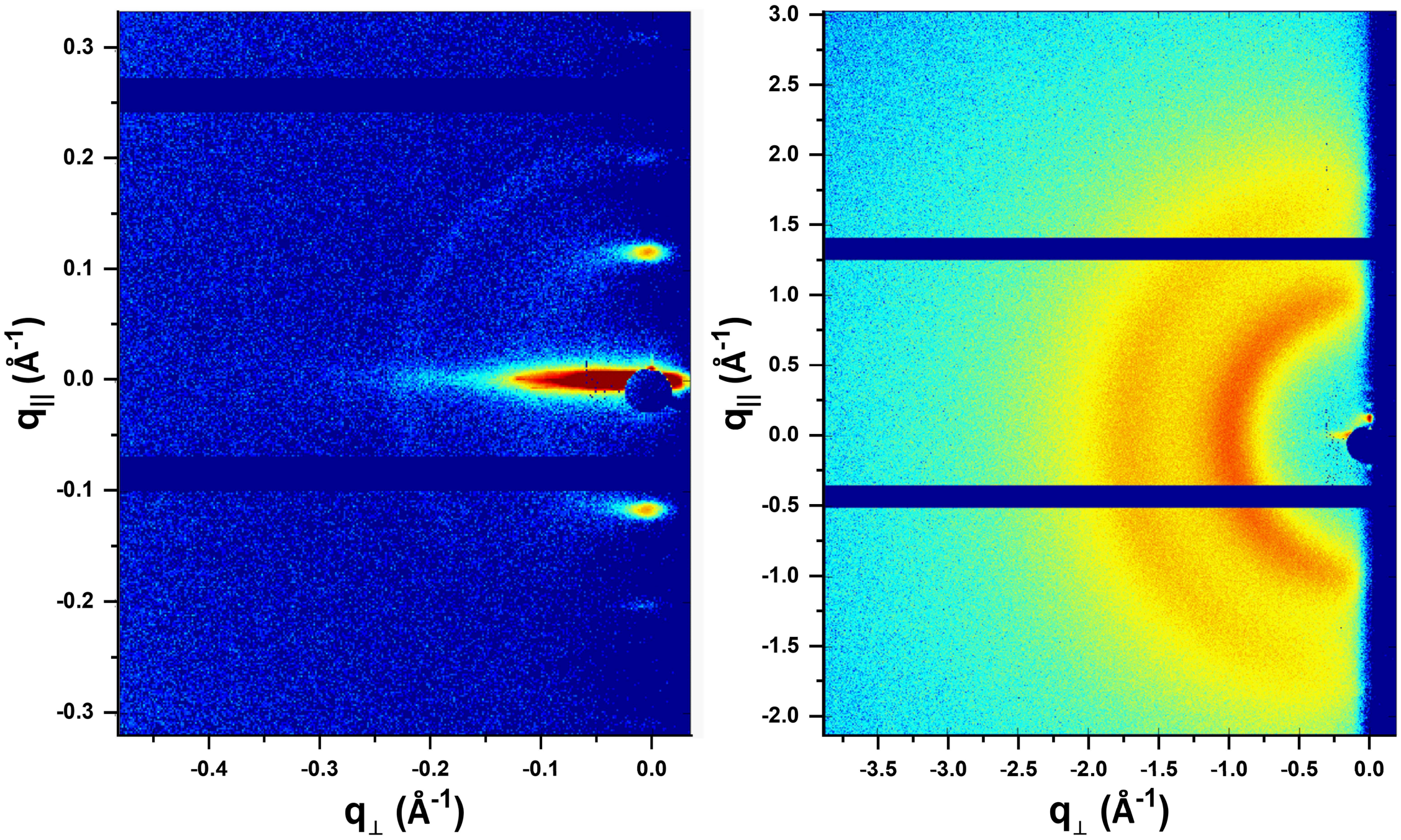

Figure S6: 2D GIMAXS (left) and GIWAXS (right) patterns of Si_{16}-C_{11} (top side with respect to casting dish).

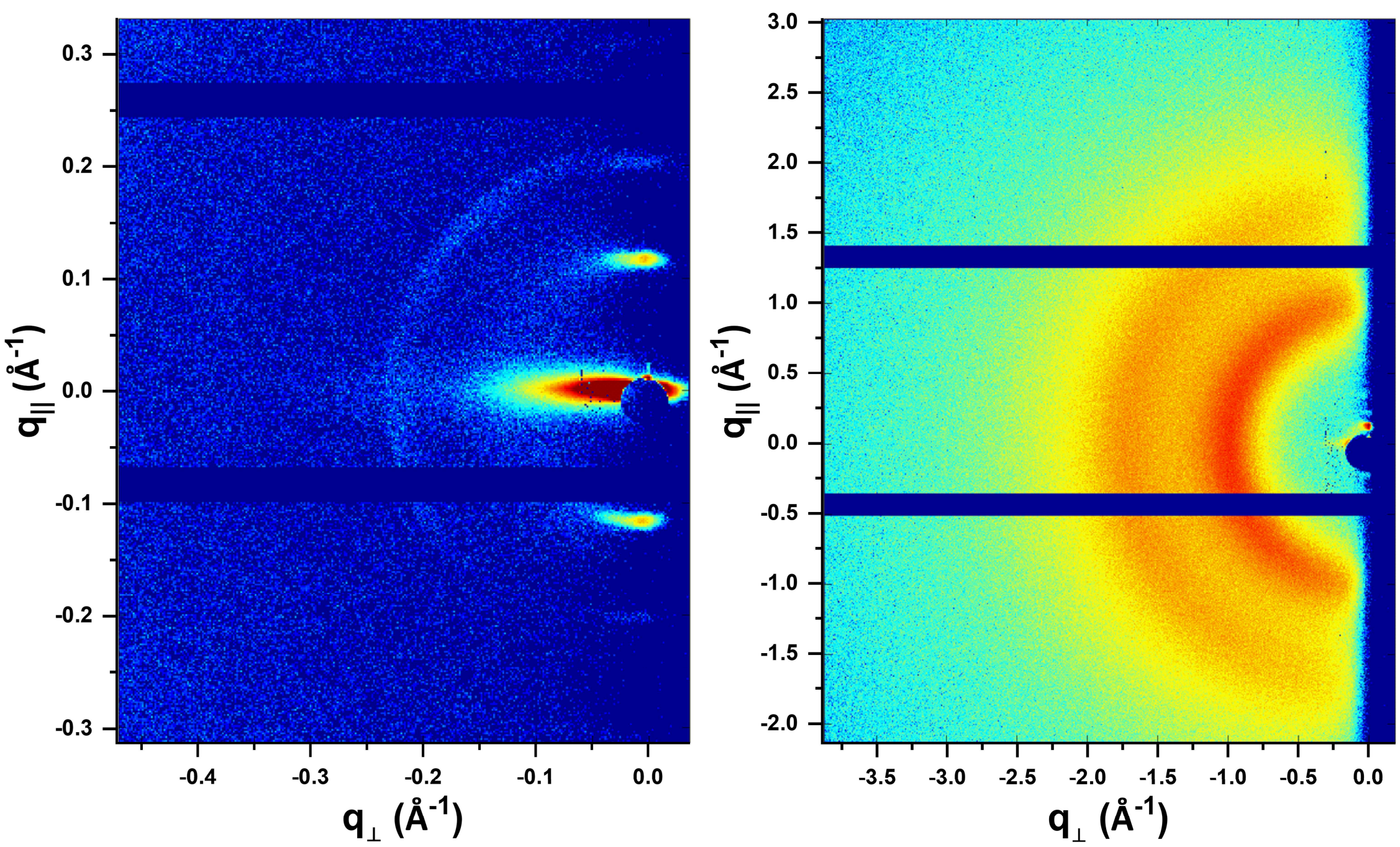

Figure S7: 2D GIMAXS (left) and GIWAXS (right) patterns of Si_{16}-C_{11} (bottom side with respect to casting dish).

Unlike Si_{16}-C_{11}, $Si_{\sim16}$-C_{11} shows no alignment in GIMAXS due to the absence of any distinct scattering rods (Figure S8).

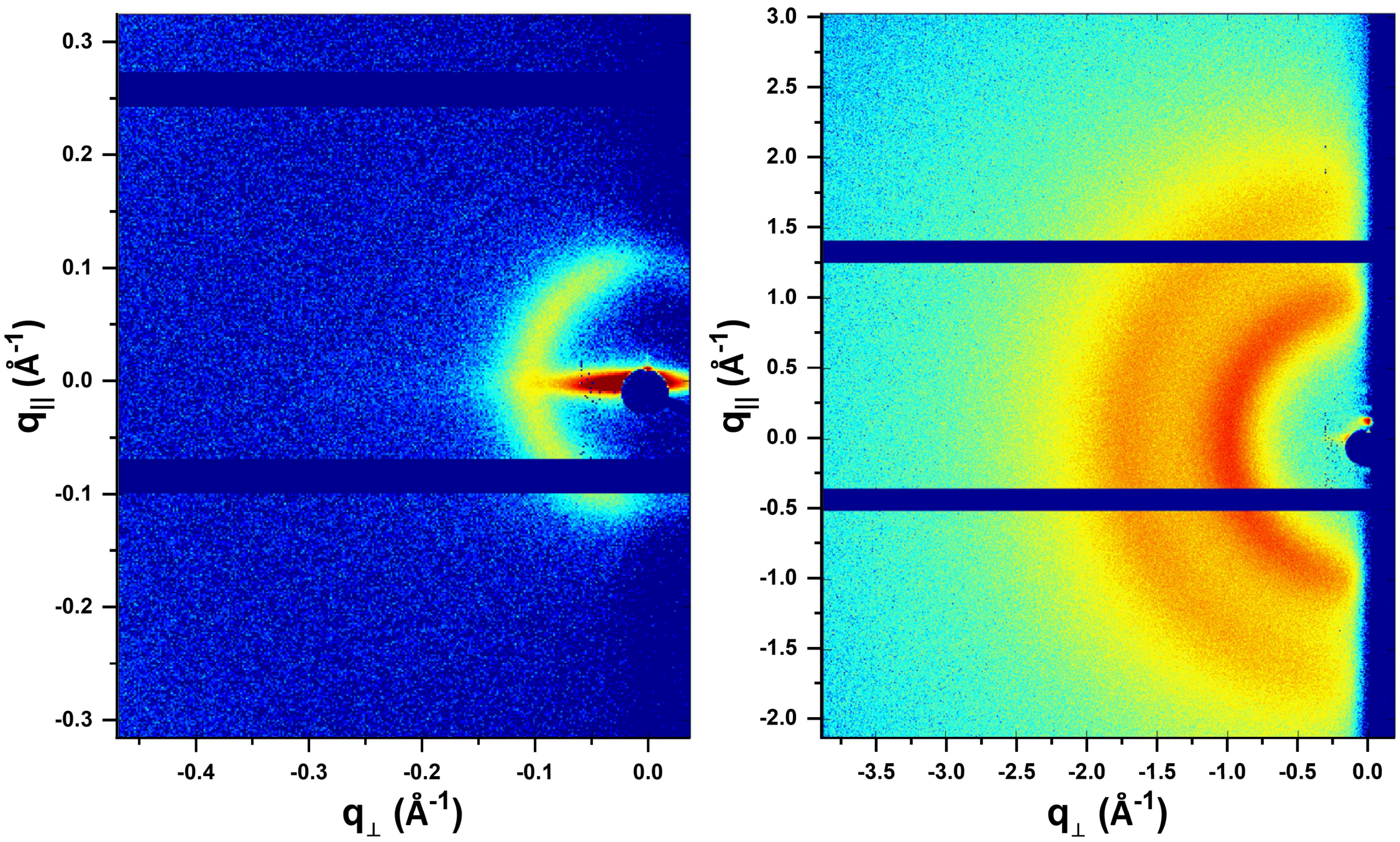

Figure S8: 2D GIMAXS (left) and GIWAXS (right) patterns of $Si_{\sim16}$-C_{11} (top side with respect to casting dish). Here, alignment of the network microstructure was not observed.

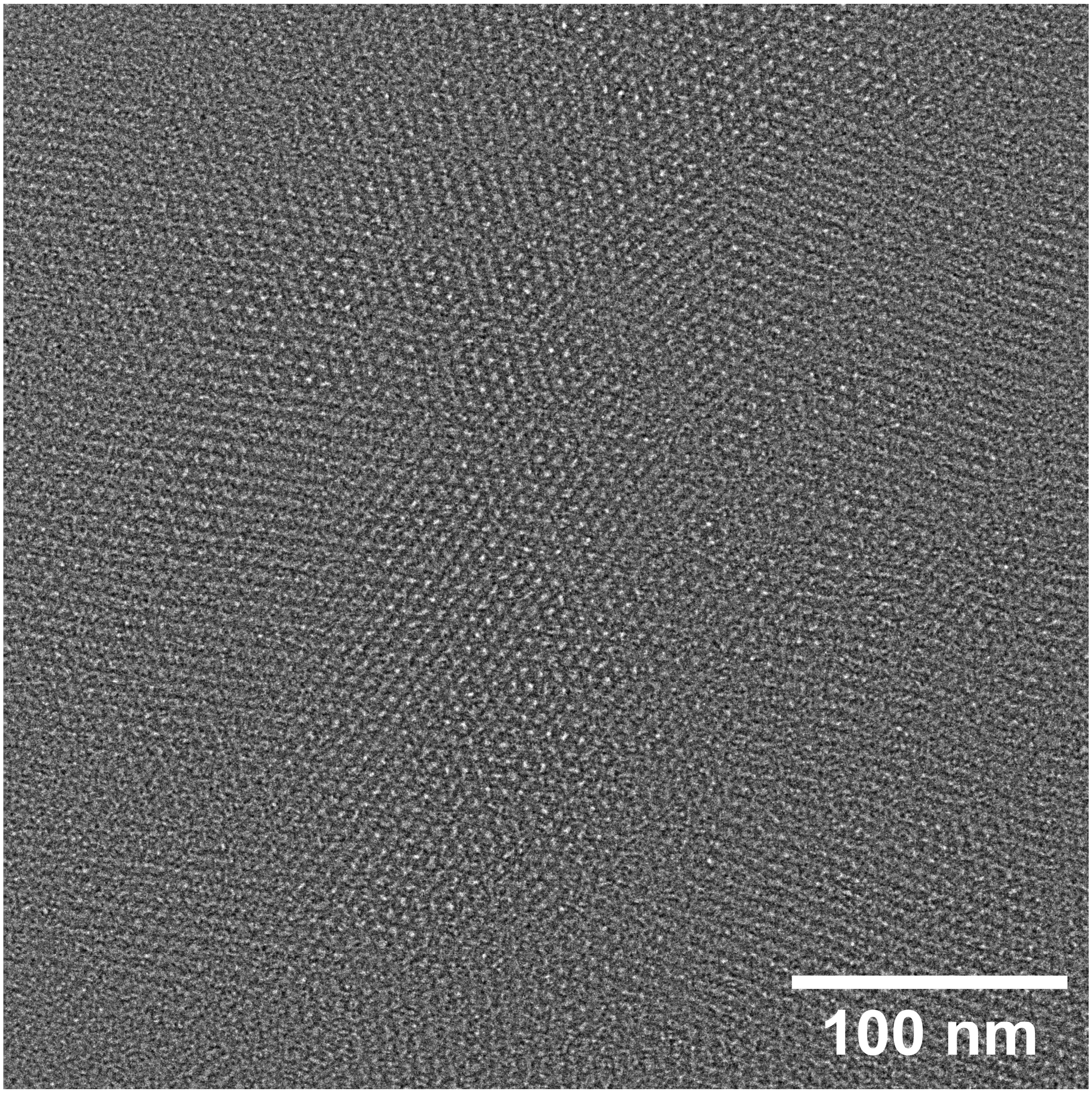

Figure S9: TEM image of Si_{16}-C_{11} sample prepared through microtomy.

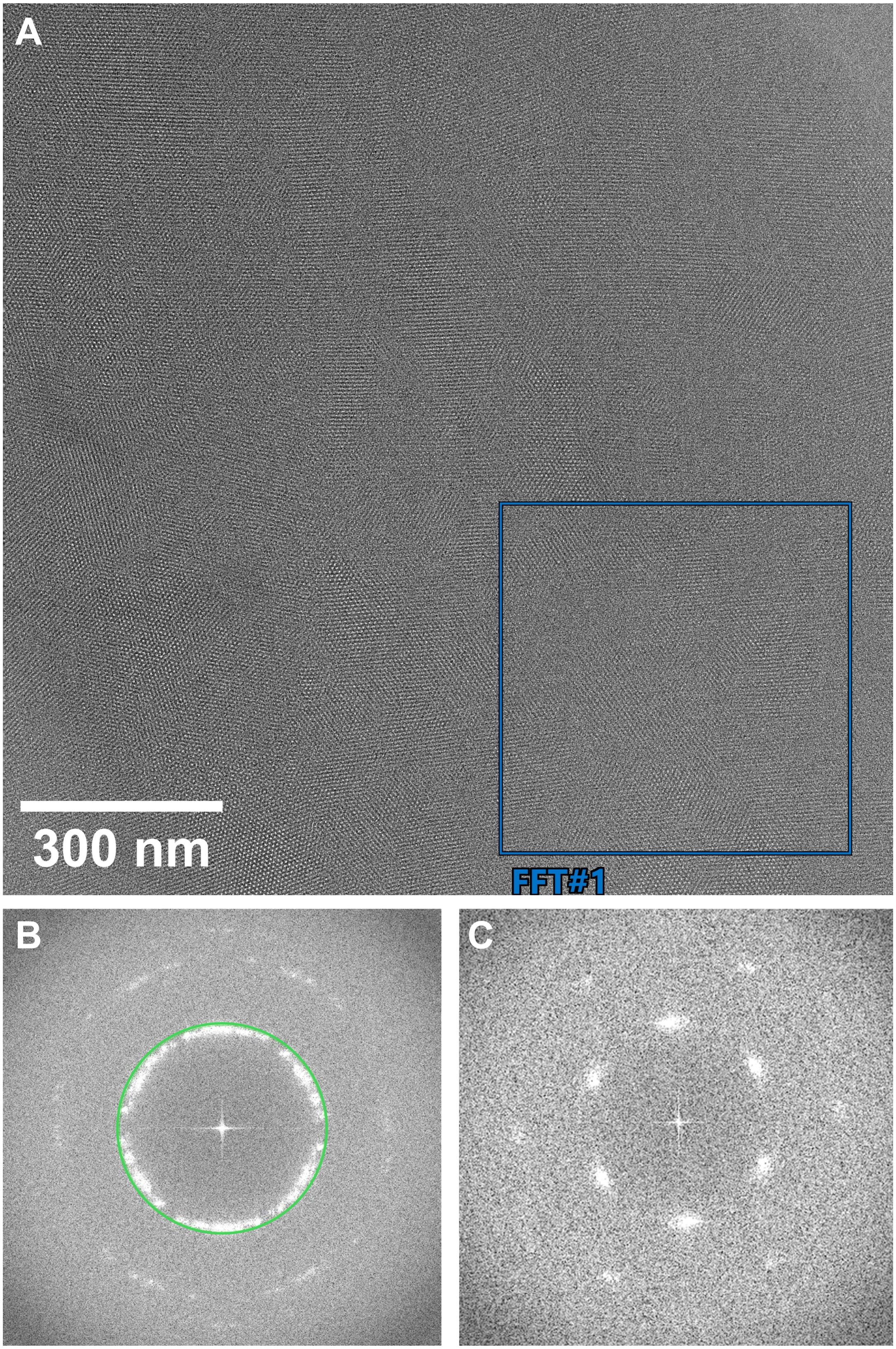

Figure S10: A) TEM image of Si_{16}-C_{11} sample prepared through microtomy. B) Reduced FFT of TEM image; green circle represents a distance of 5.5 nm. C) Reduced FFT of the selected area #1, displaying a hexagonal symmetry.

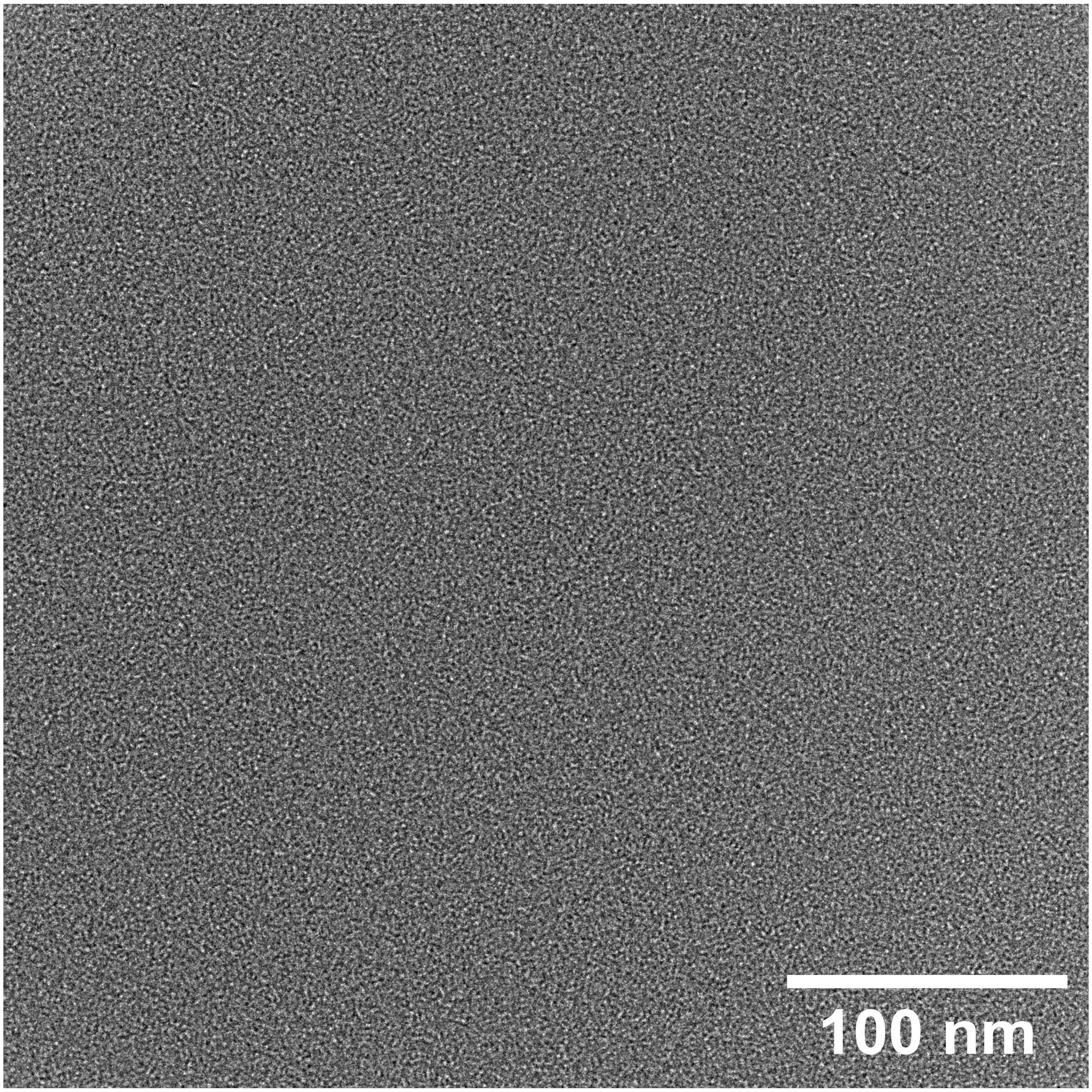

Figure S11: TEM image of $Si_{\sim16}$-C_{11} sample prepared through microtomy.

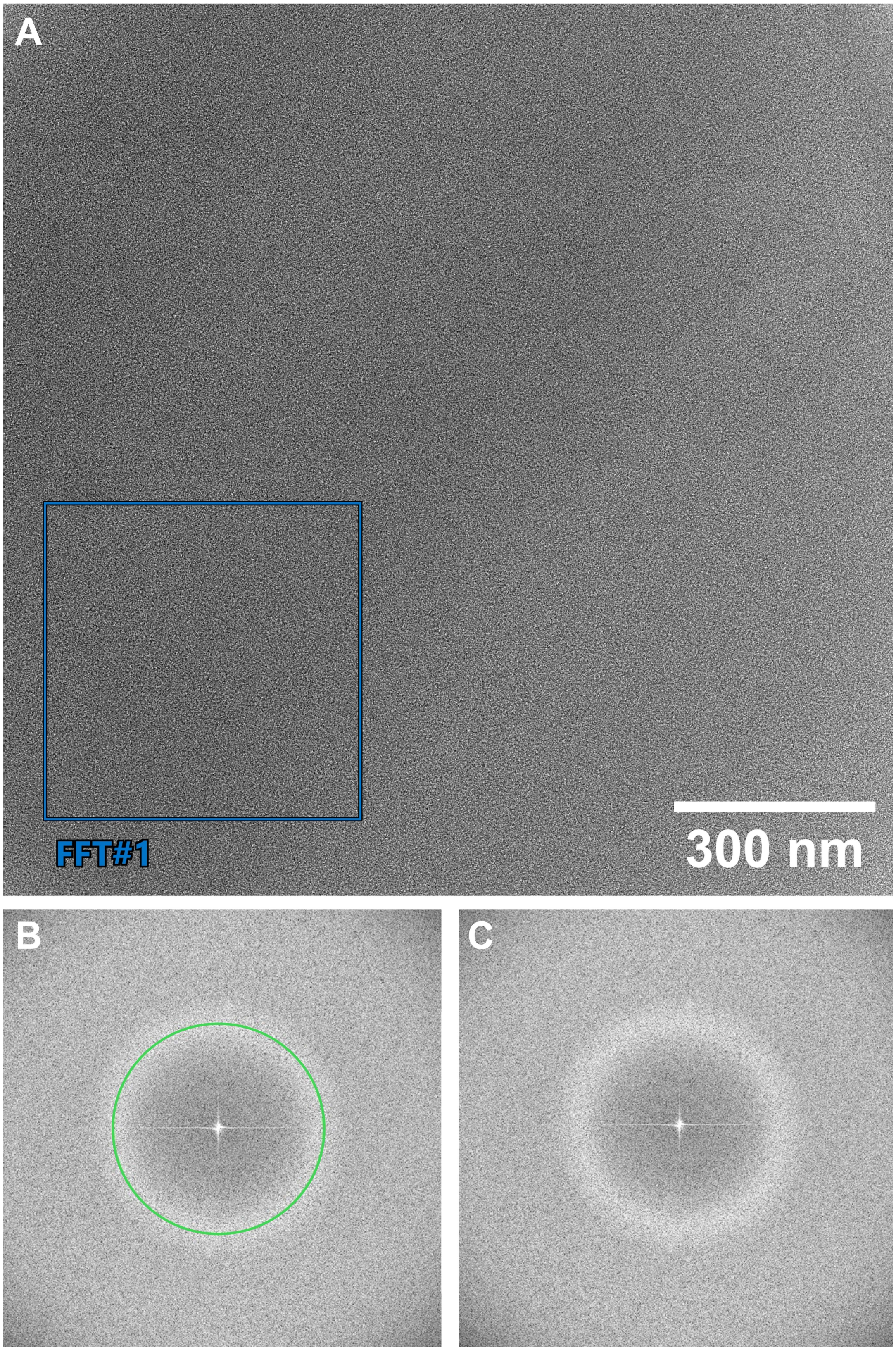

Figure S12: A) TEM image of $Si_{\sim16}$-C_{11} sample prepared through microtomy showing no ordered structures. B) Reduced FFT of TEM image; green circle represents a distance of 6.3 nm. C) Reduced FFT of the selected area #1.

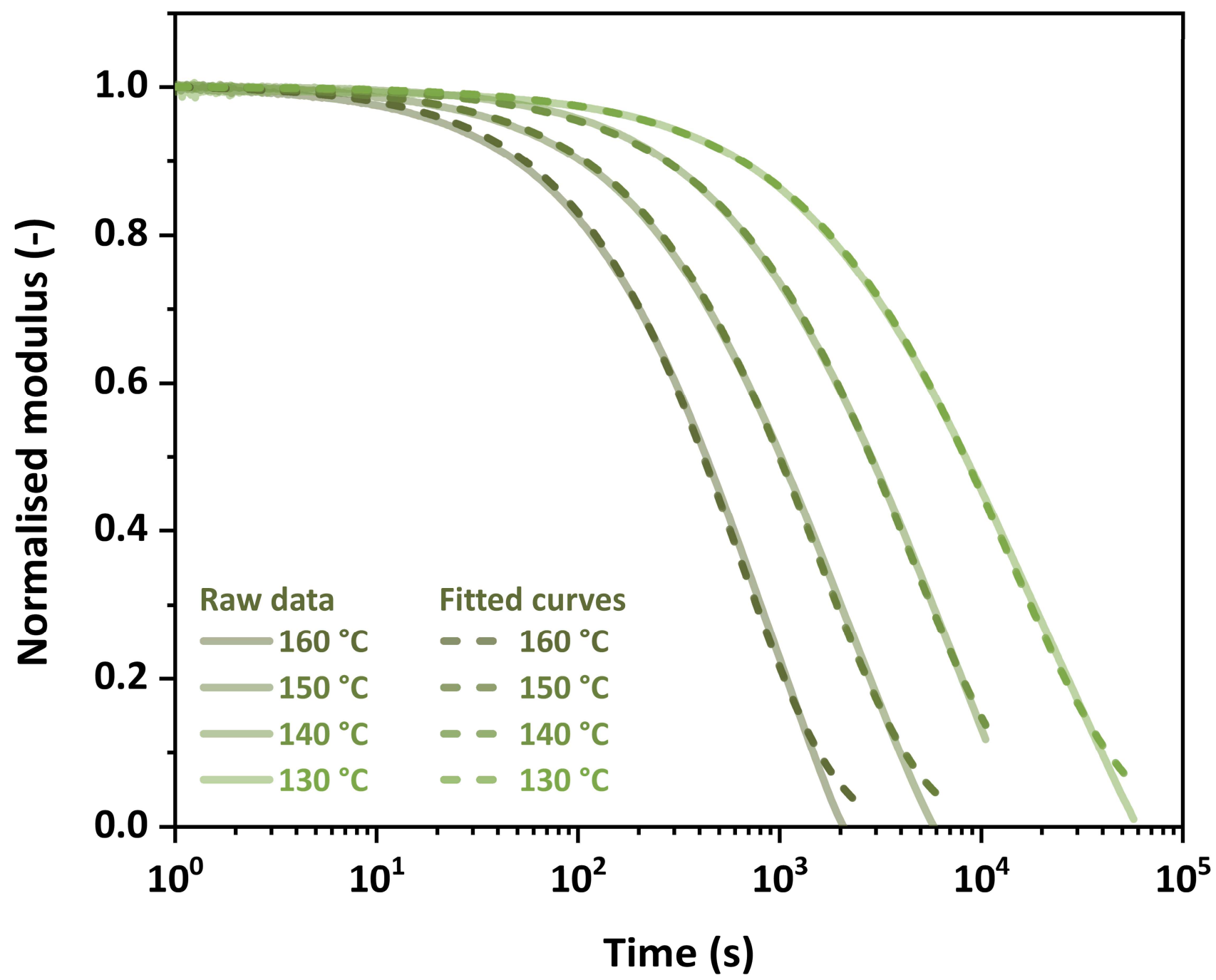

Figure S13: Stress relaxation curves in tension of the ordered Si_{16}-C_{11} network and curves fitted to the stretched exponential equation at various temperatures and 5 % strain.

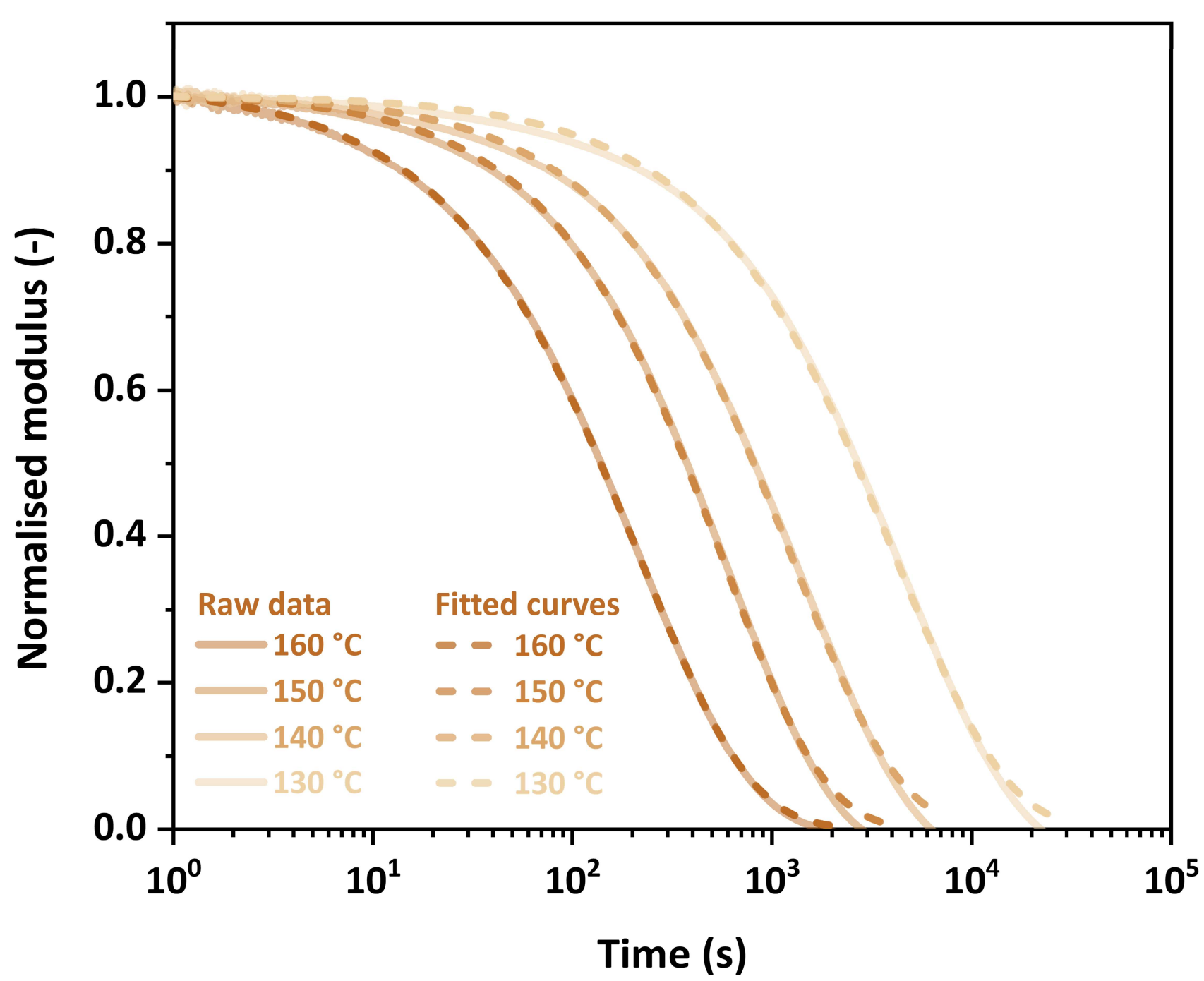

Figure S14: Stress relaxation curves in tension of the disordered $Si_{\sim 16}$-C_{11} network and curves fitted to the stretched exponential equation at various temperatures and 5 % strain.

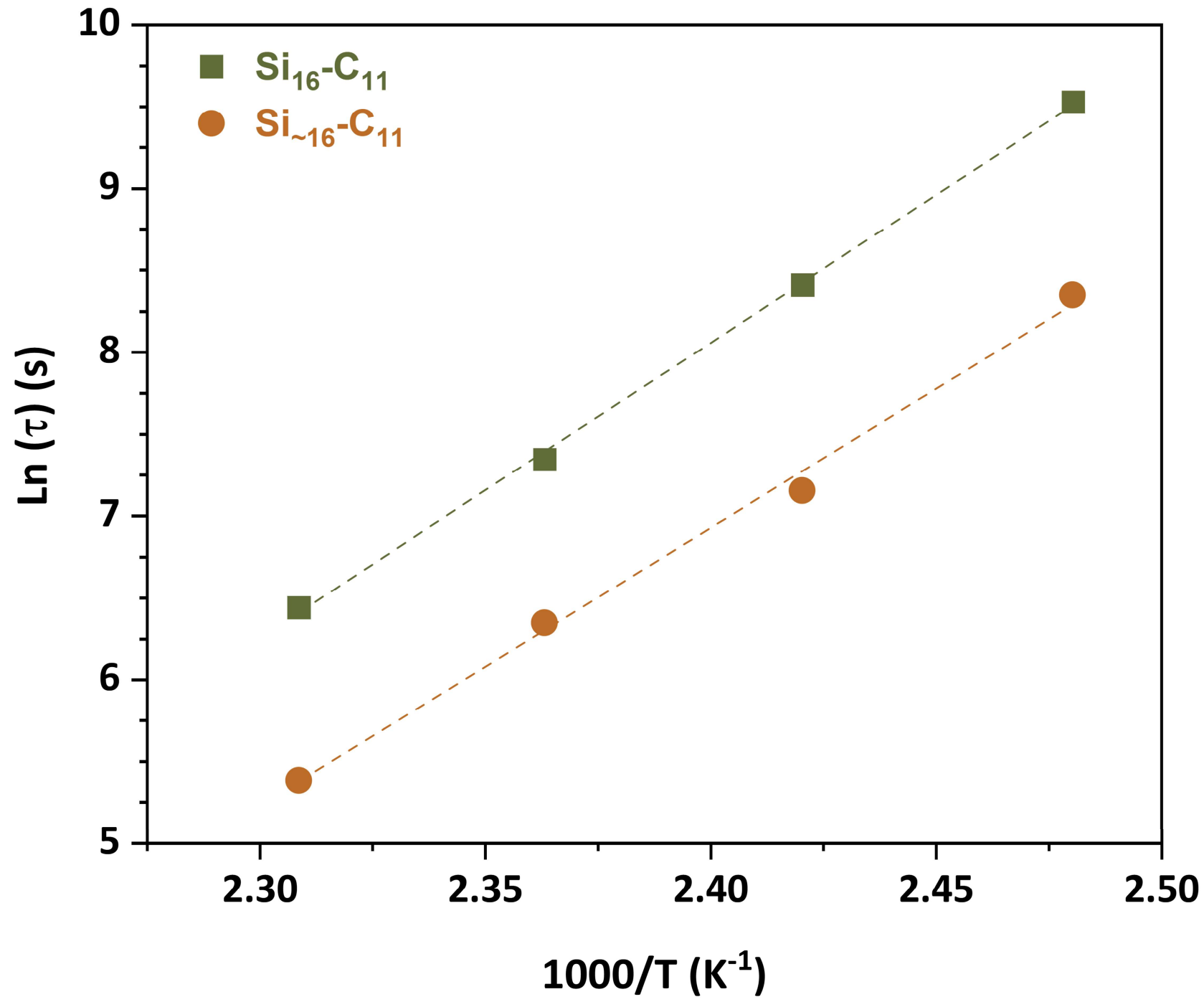

Figure S15: Arrhenius plot of characteristic relaxation times (using τ) of the Si_{16}-C_{11} and $Si_{\sim16}$-C_{11} networks.

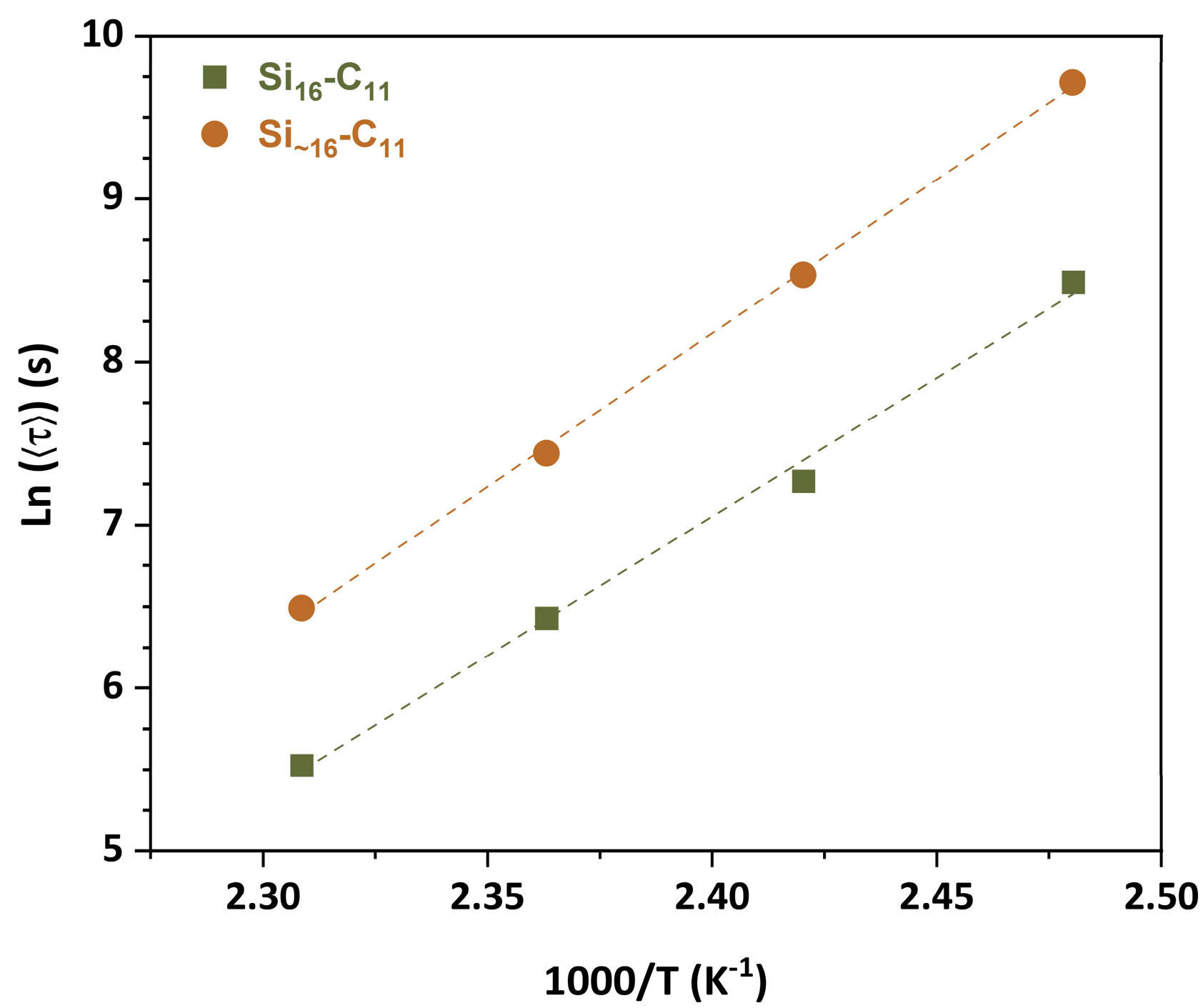

Figure S16: Arrhenius plot of characteristic relaxation times (using $\langle\tau\rangle$) of the Si_{16}-C_{11} and $Si_{\sim16}$-C_{11} networks.

3. Characterization data

3.1 SEC

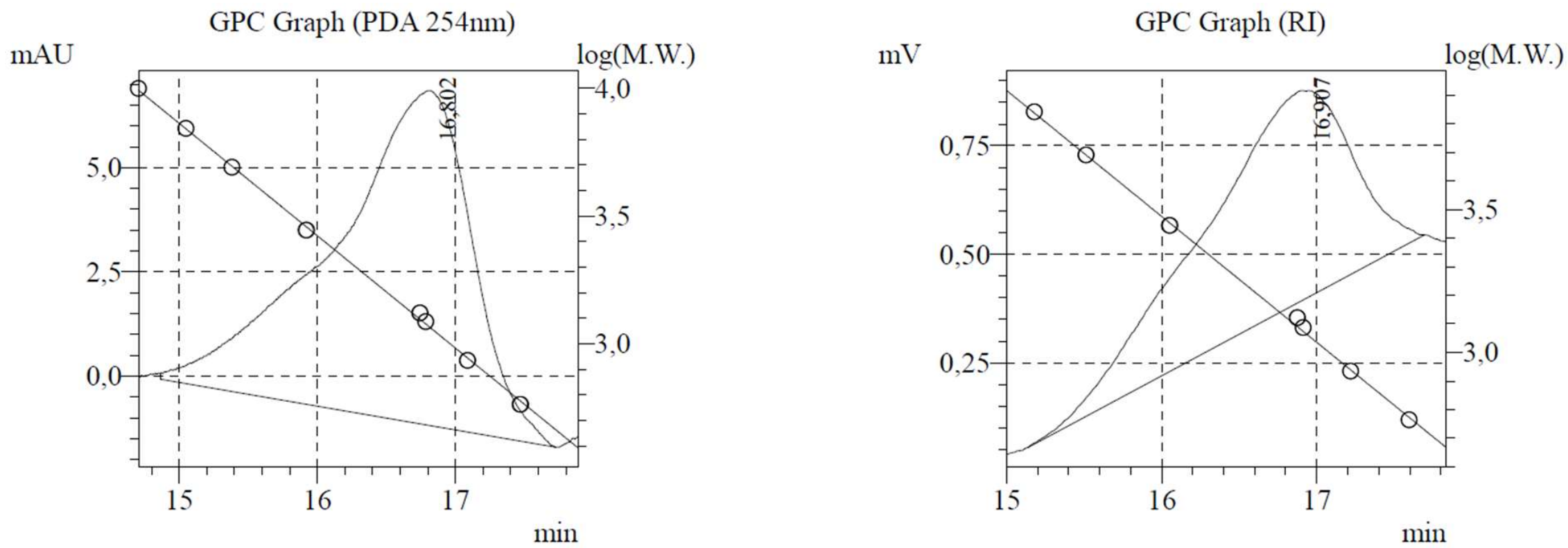

Figure S17: SEC chromatogram (PDA detector (left and RI detector (right) of $Si_{\sim16}$-C_{11} dihydrazide. Note: There are interactions of the molecule with the column which gives rise to the tailing in the SEC trace.

3.2 Gel fraction and swelling ratio

Table S3: Gel fractions and swelling ratios of Si_{16}-C_{11} and $Si_{\sim16}$-C_{11} networks.

Network	Gel fraction (%)	Swelling ratio (-)
**Si_{16}-C_{11}**	92 ± 1	4.2 ± 0.8
**$Si_{\sim16}$-C_{11}**	88 ± 1	5.4 ± 1.1

3.3 TGA and DSC

The TGA curve and DSC thermogram of C_{11}-$Si_{\sim16}$ have been reported in our previous work as the M-PDMS network.[3]

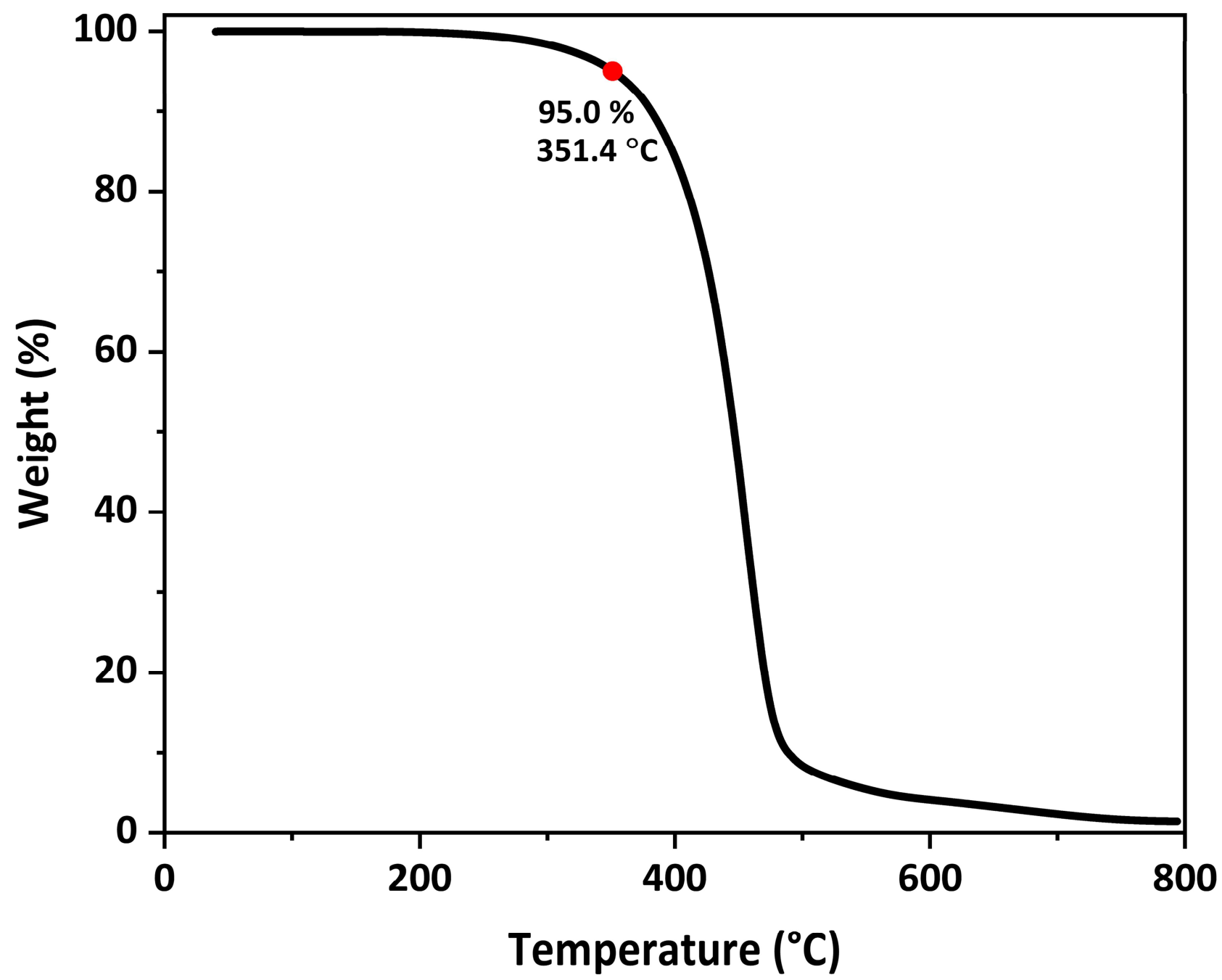

Figure S18: TGA curve of the cured Si_{16}-C_{11} network.

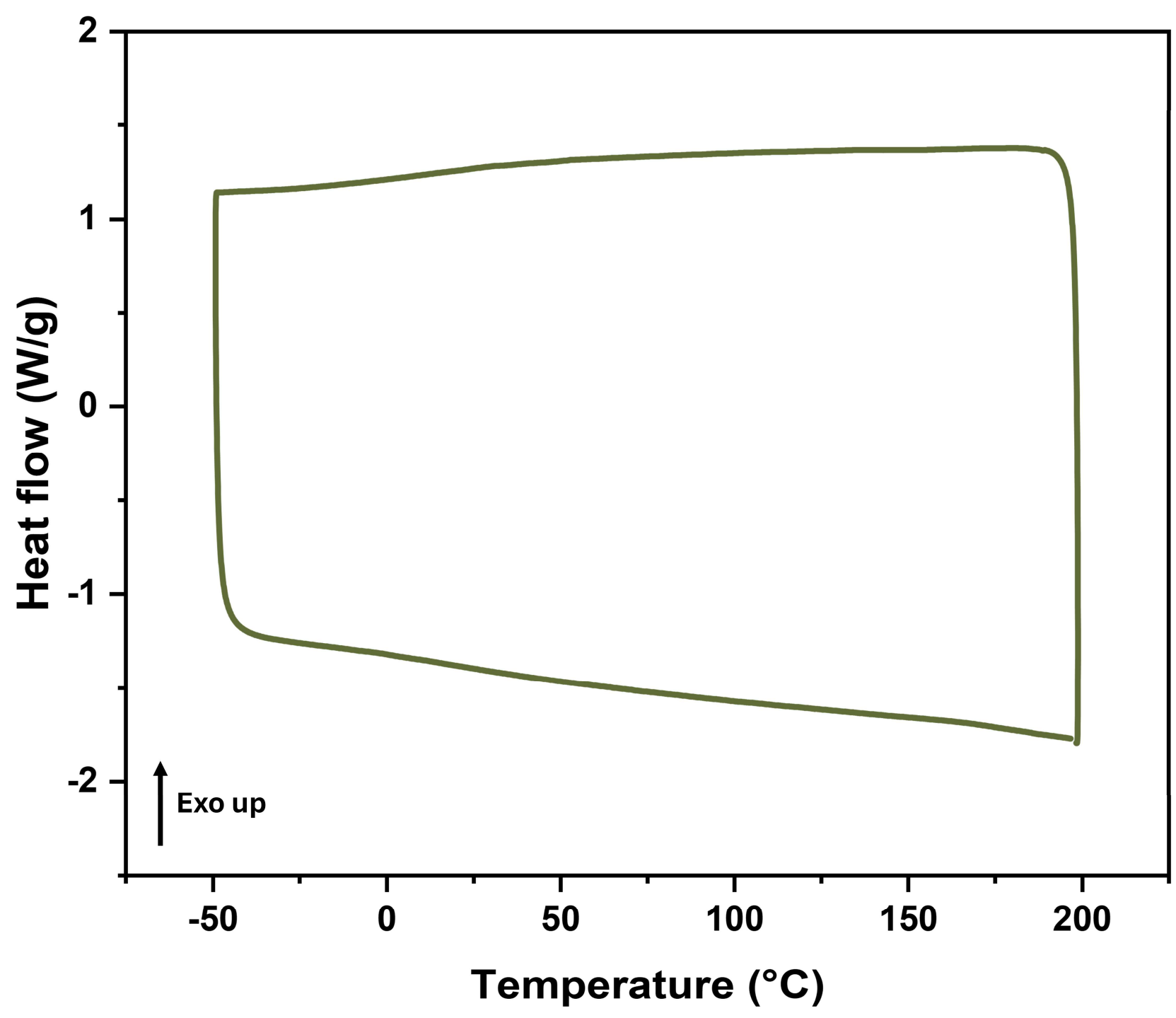

Figure S19: DSC thermogram of the cured Si_{16}-C_{11} network. Heating and cooling rates are 10 °C/min.

3.4 NMR and MALDI-ToF-MS

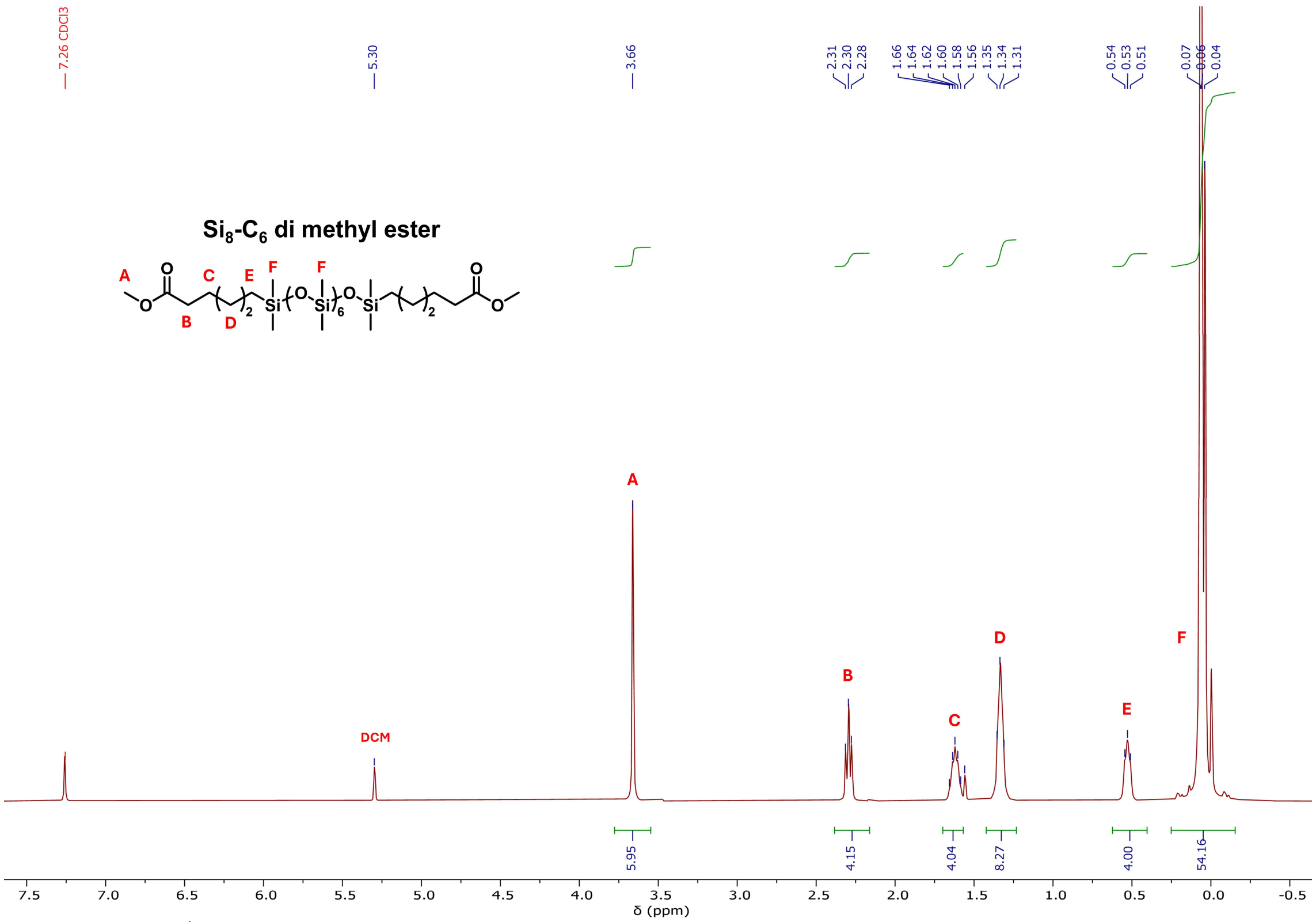

Figure S20: ^1H NMR spectrum of Si$_8$-C$_6$ dimethyl ester in $CDCl_3$.

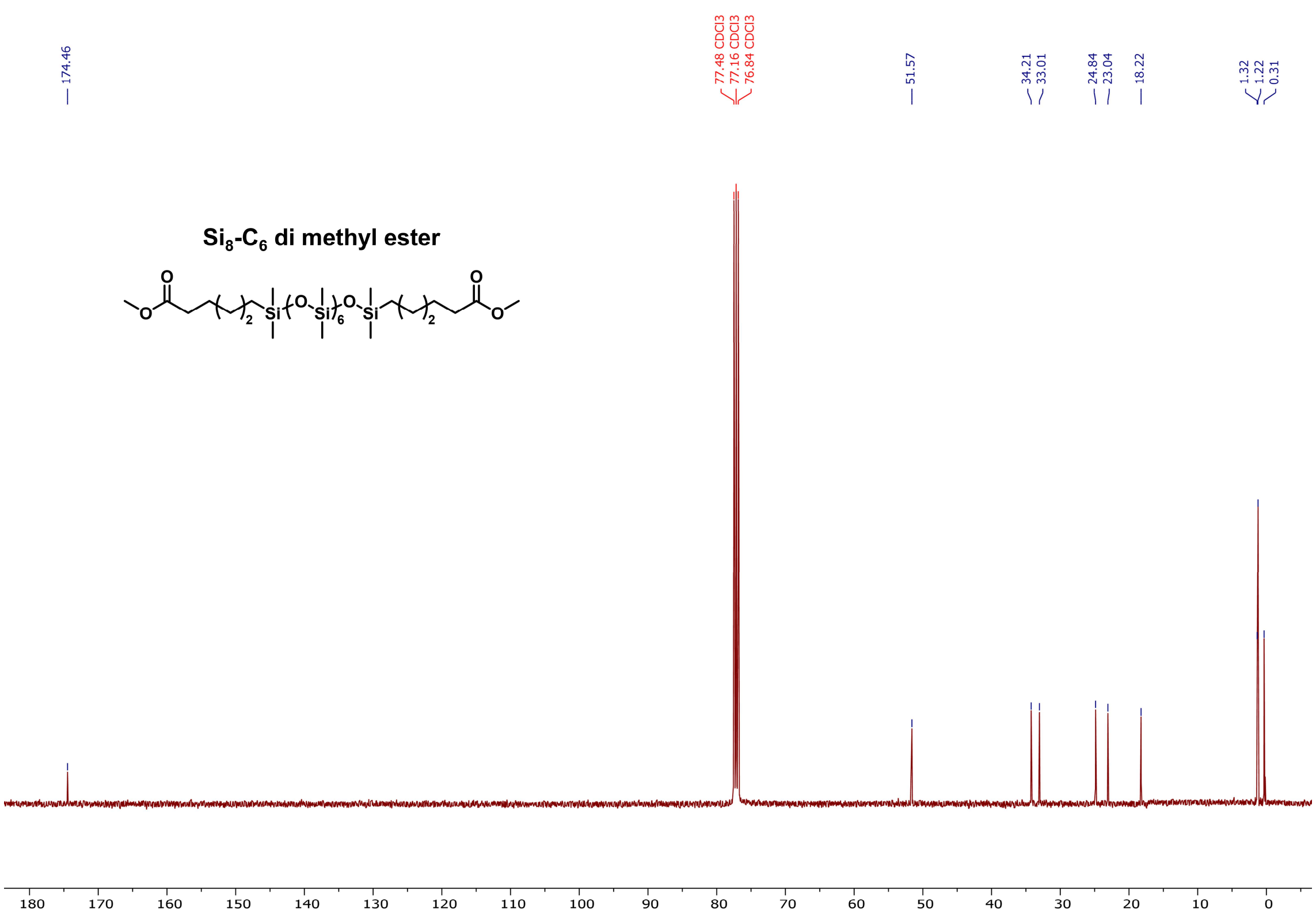

Figure S21: ^{13}C NMR spectrum of Si$_8$-C$_6$ dimethyl ester in $CDCl_3$.

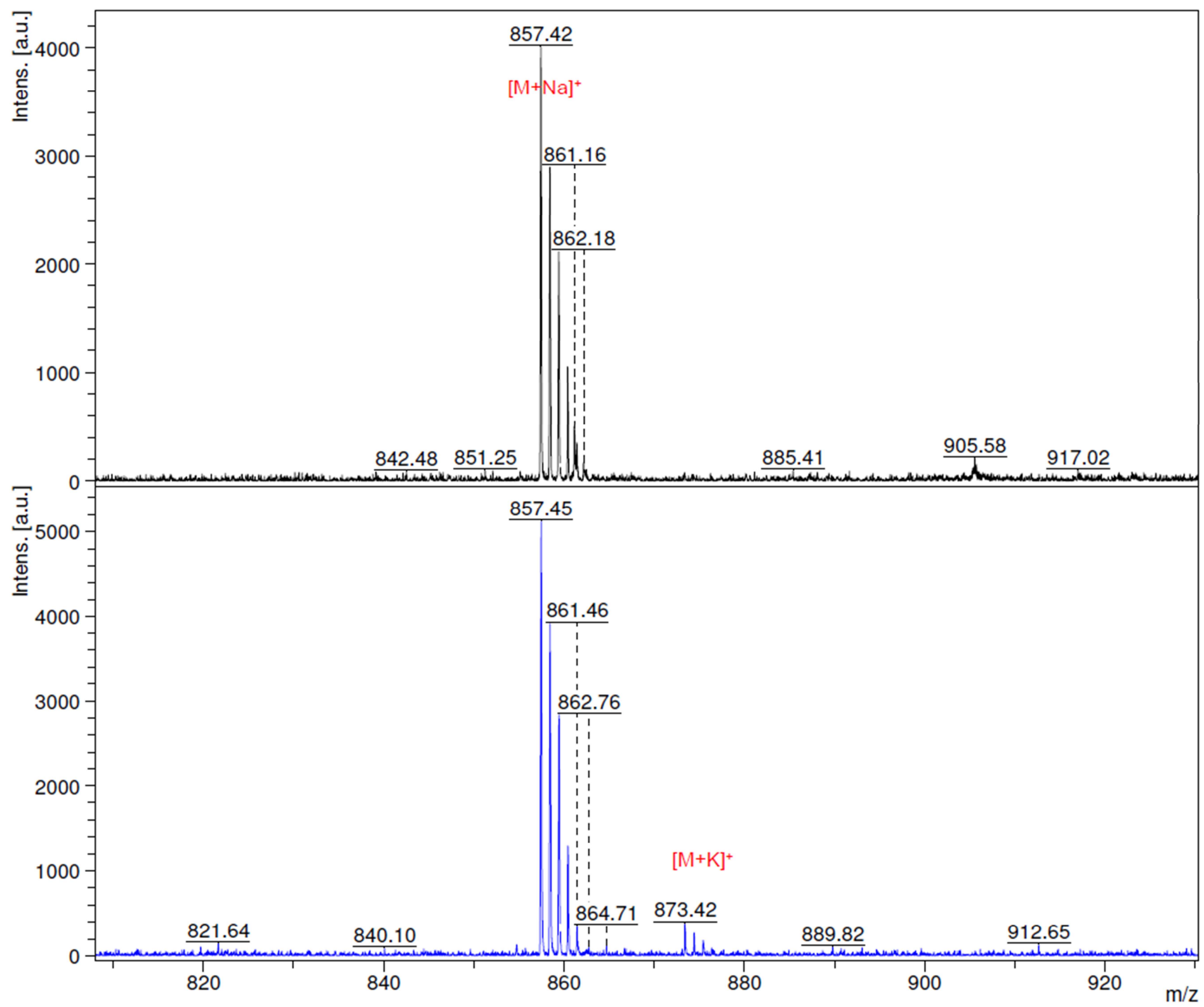

Figure S22: MALDI-ToF-MS spectra of Si_8-C_6 dimethyl ester (black: CHCA, blue: DCTB matrix).

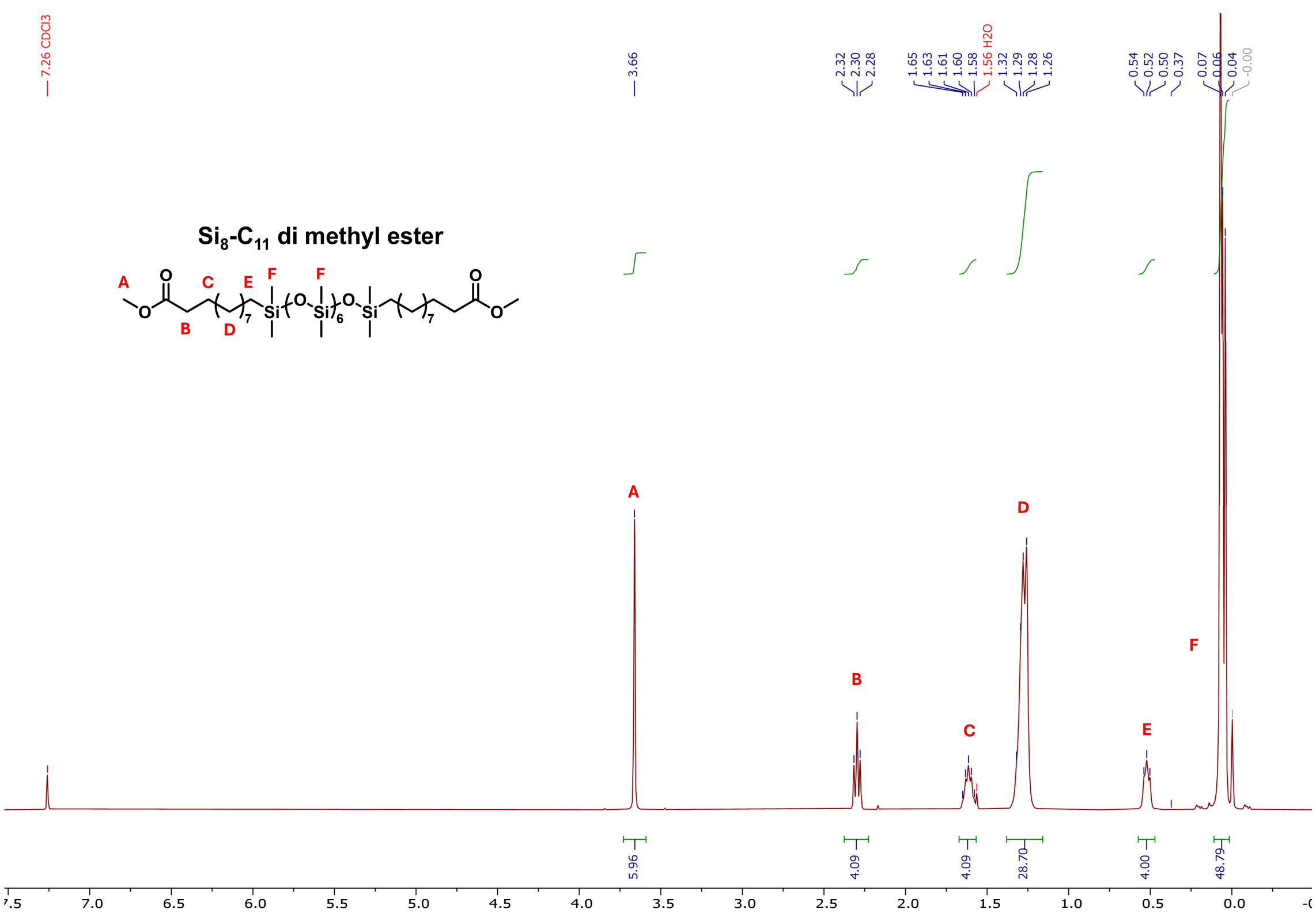

Figure S23: ^{1}H NMR spectrum of Si_8-C_{11} dimethyl ester in $CDCl_3$.

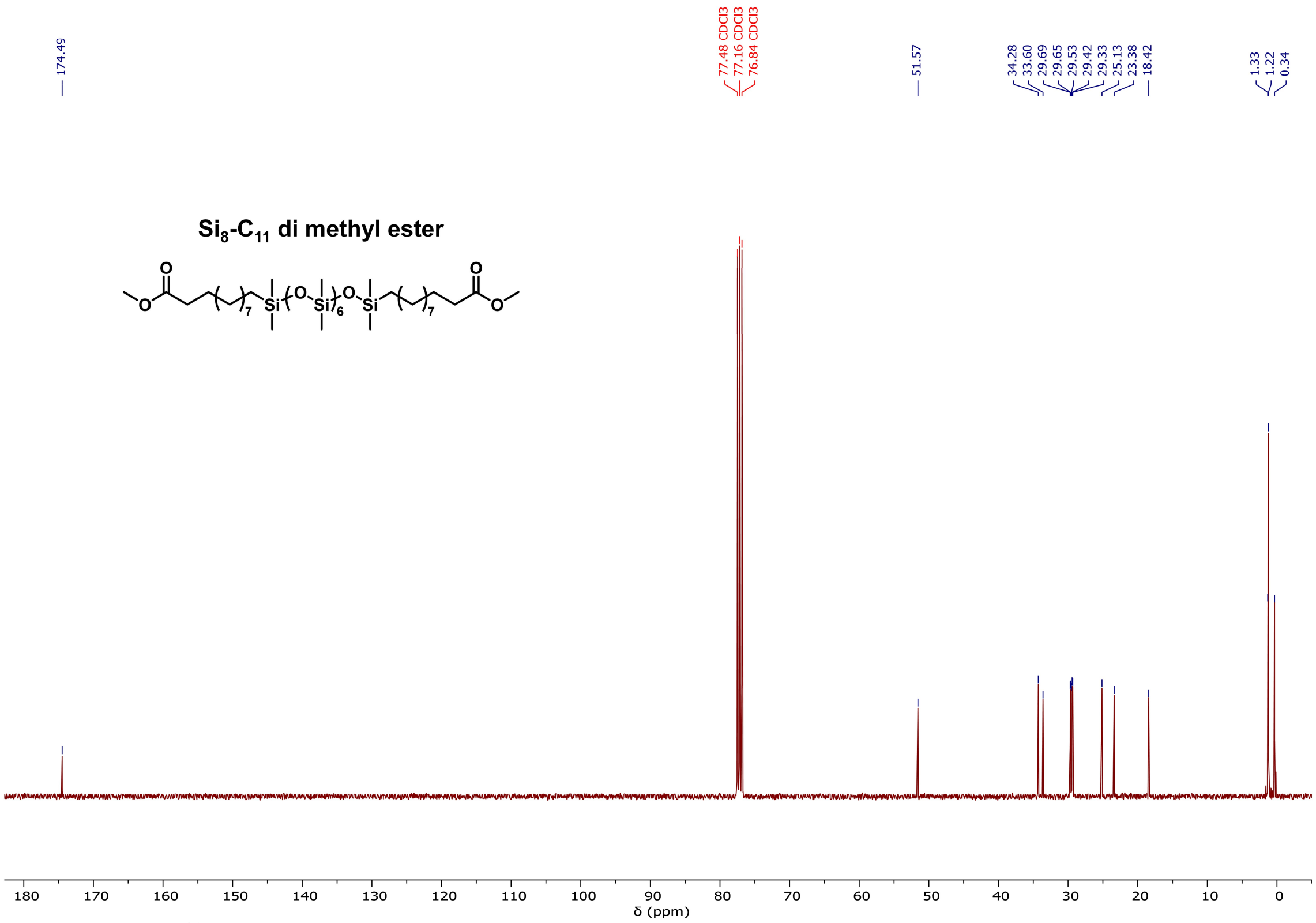

Figure S24: ^{13}C NMR spectrum of Si_8-C_{11} dimethyl ester in $CDCl_3$.

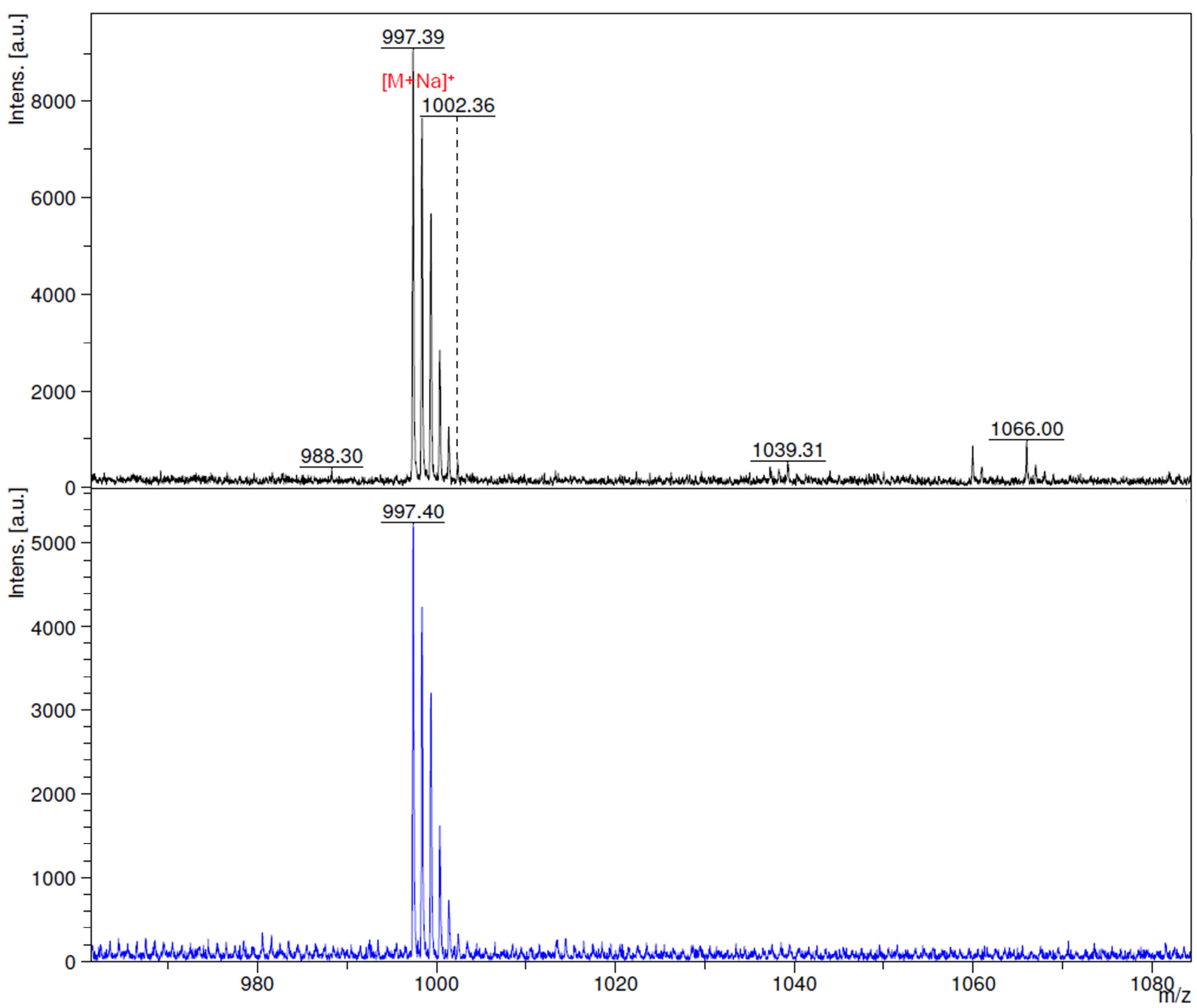

Figure S25: MALDI-ToF-MS spectra of Si_8-C_{11} dimethyl ester (black: CHCA, blue: DCTB matrix).

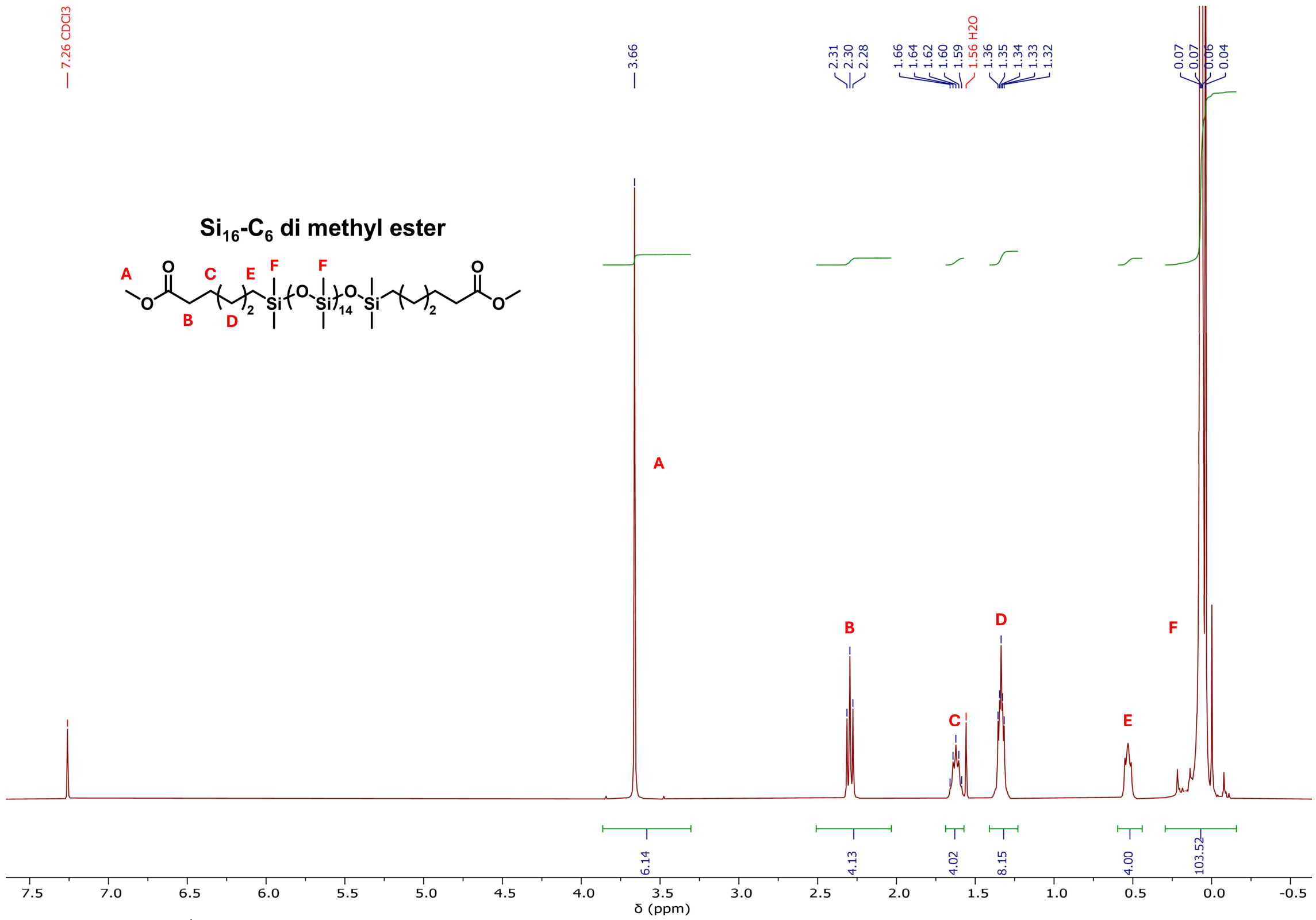

Figure S26: ^{1}H NMR spectrum of Si_{16}-C_6 dimethyl ester in $CDCl_3$.

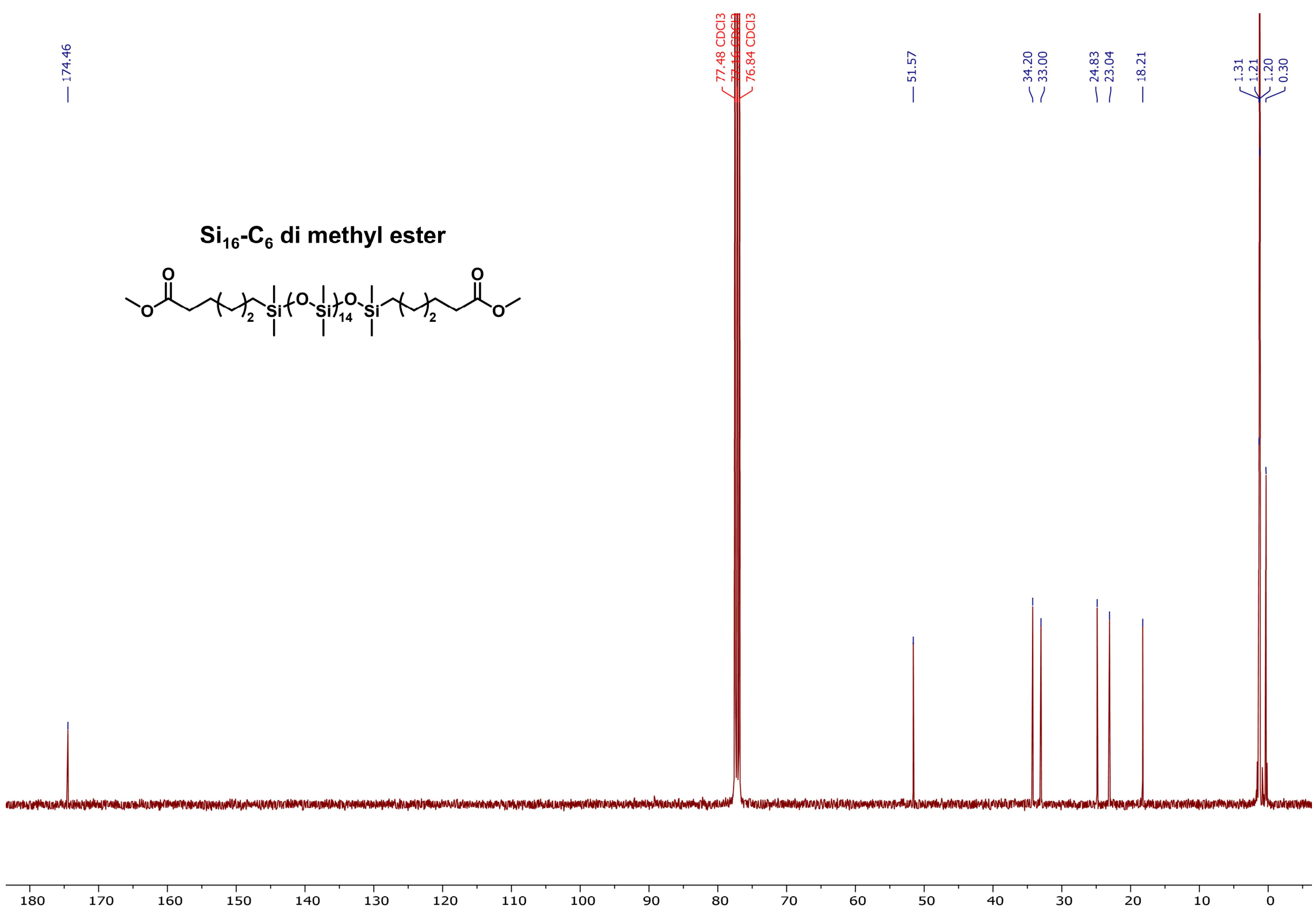

Figure S27: ^{13}C NMR spectrum of Si_{16}-C_6 dimethyl ester in $CDCl_3$.

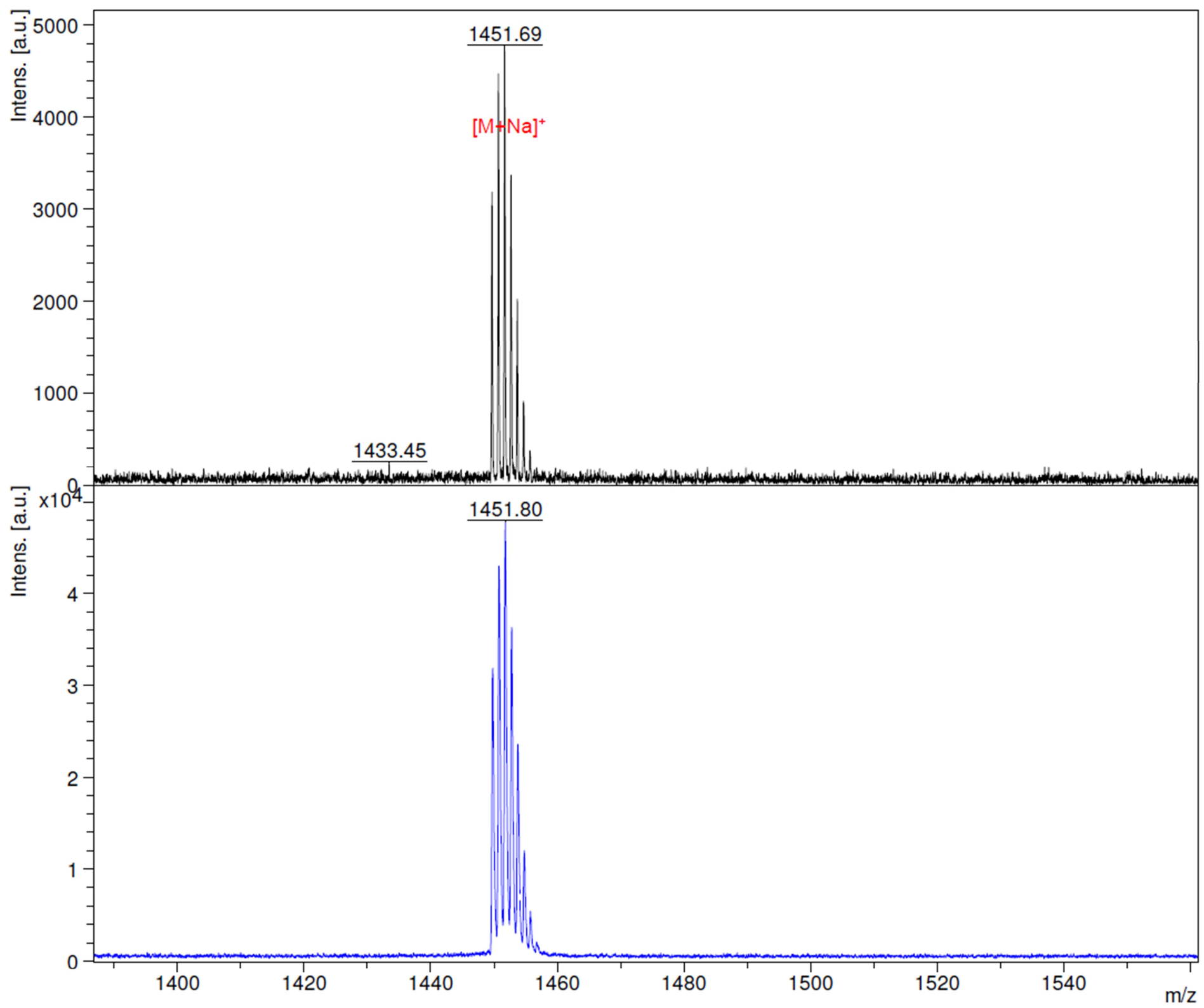

Figure S28: MALDI-ToF-MS spectra of Si_{16}-C_6 dimethyl ester (black: CHCA, blue: DCTB matrix).

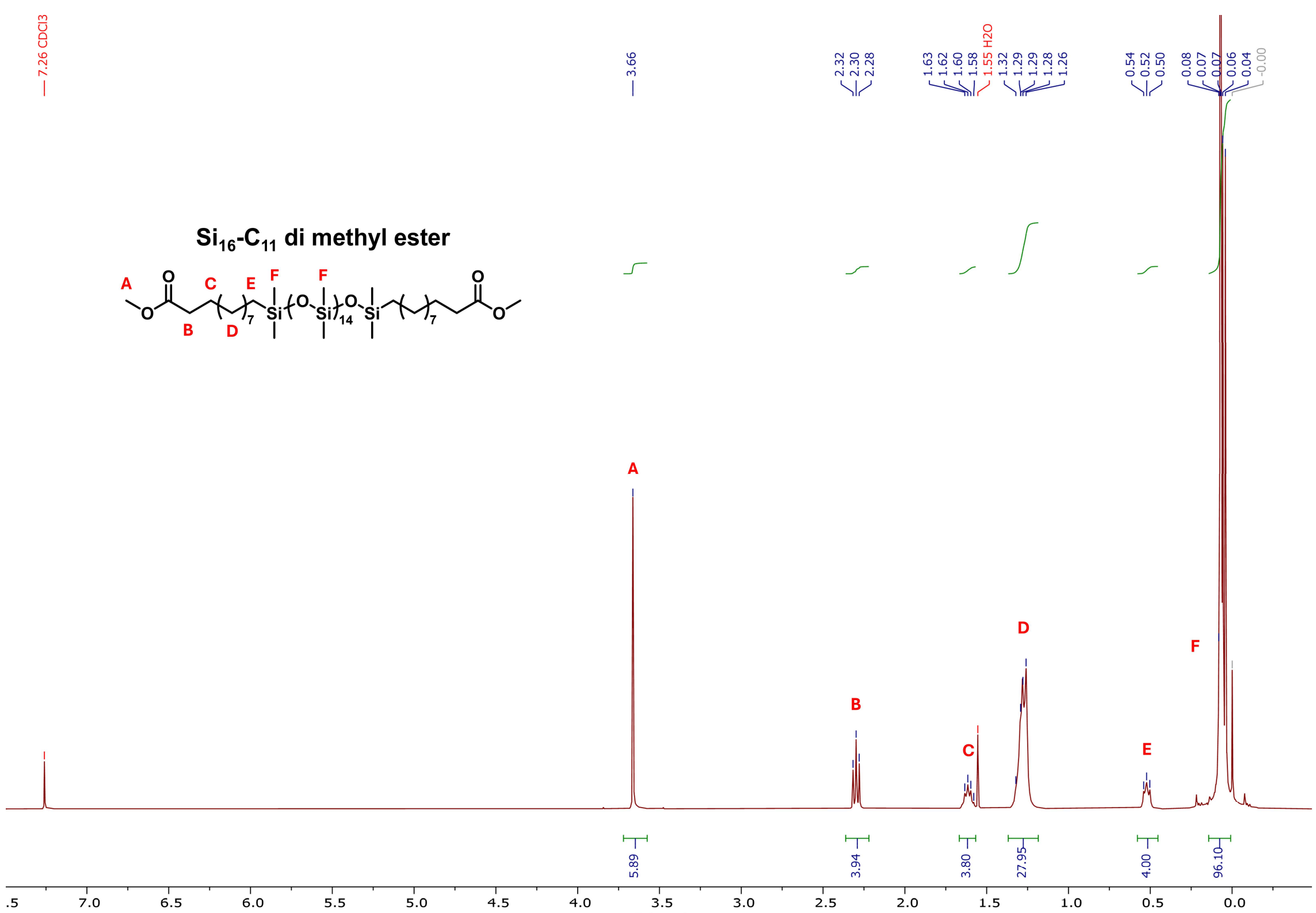

Figure S29: ^{1}H NMR spectrum of Si_{16}-C_{11} dimethyl ester in $CDCl_3$.

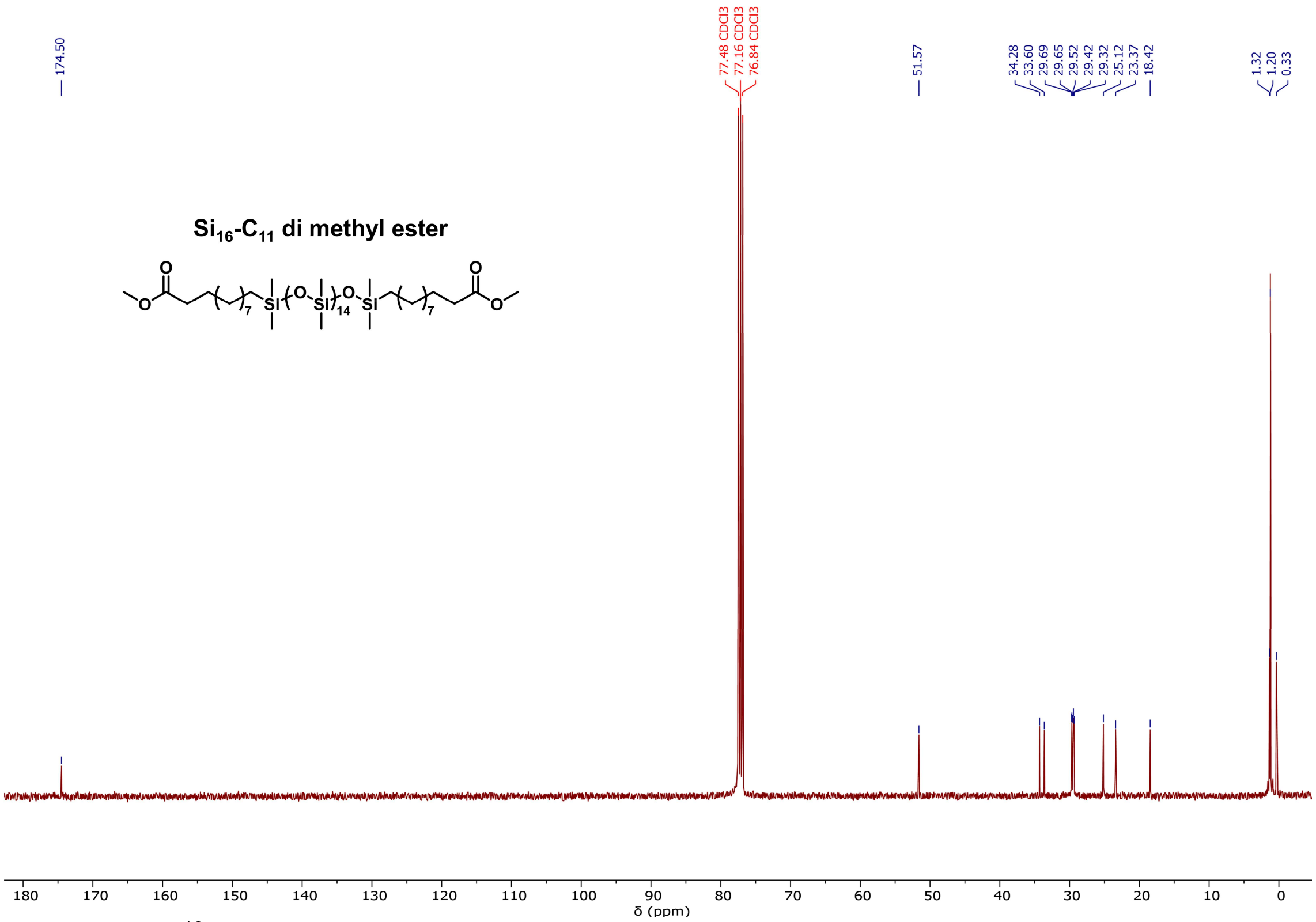

Figure S30: ^{13}C NMR spectrum of Si_{16}-C_{11} dimethyl ester in $CDCl_3$.

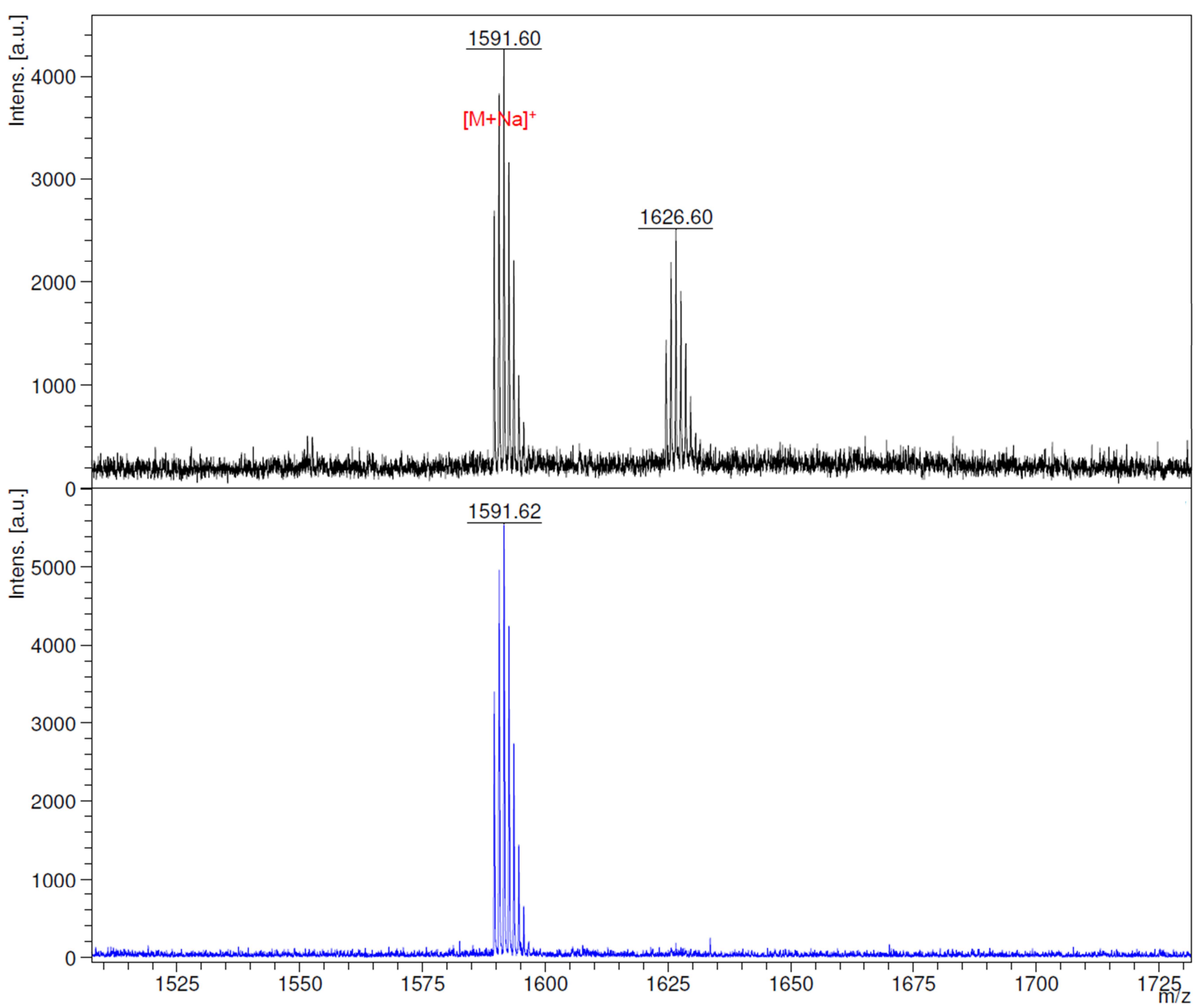

Figure S31: MALDI-ToF-MS spectra of Si_{16}-C_{11} dimethyl ester (black: CHCA, blue: DCTB matrix).

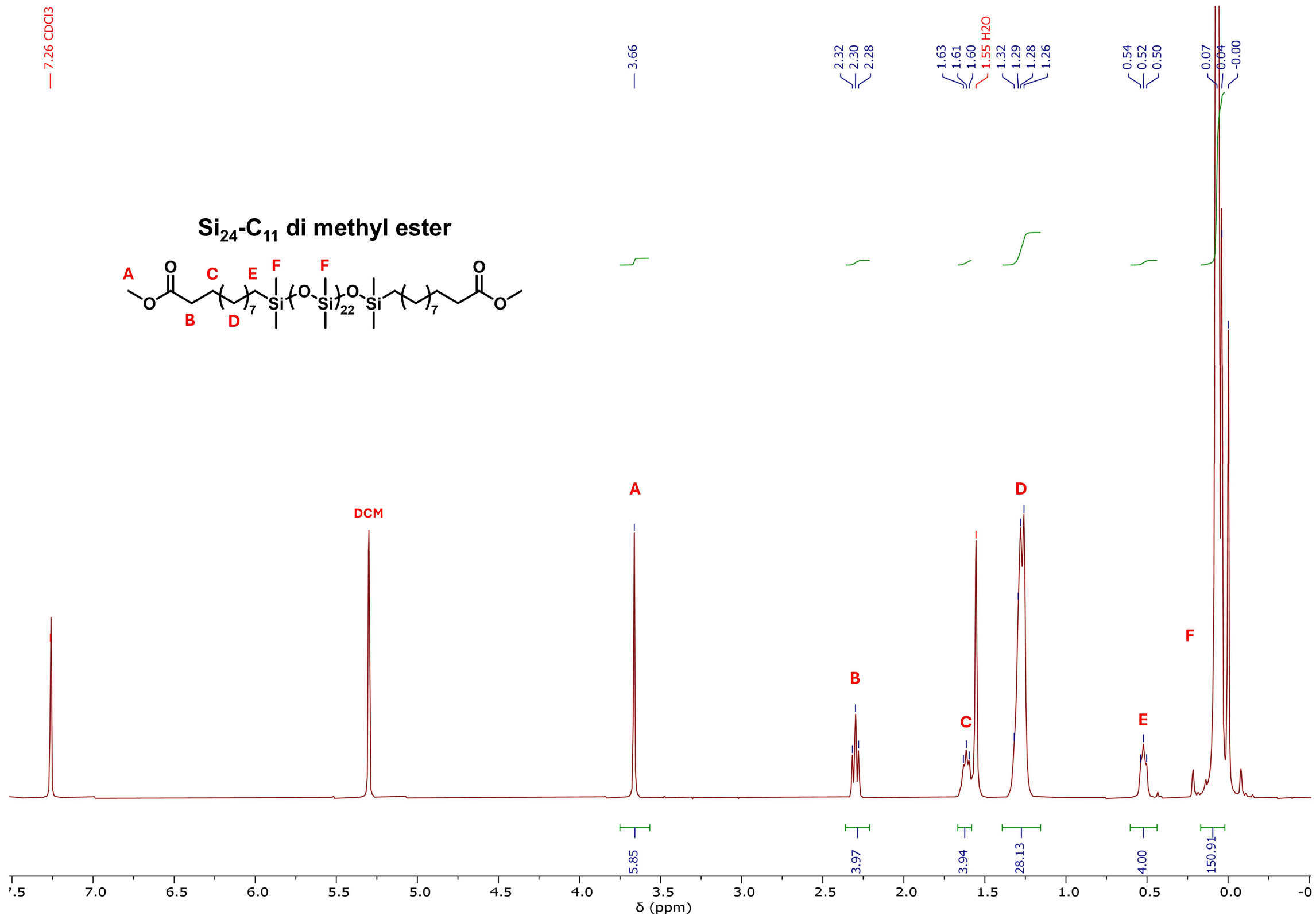

Figure S32: ^{1}H NMR spectrum of Si_{24}-C_{11} dimethyl ester in $CDCl_3$.

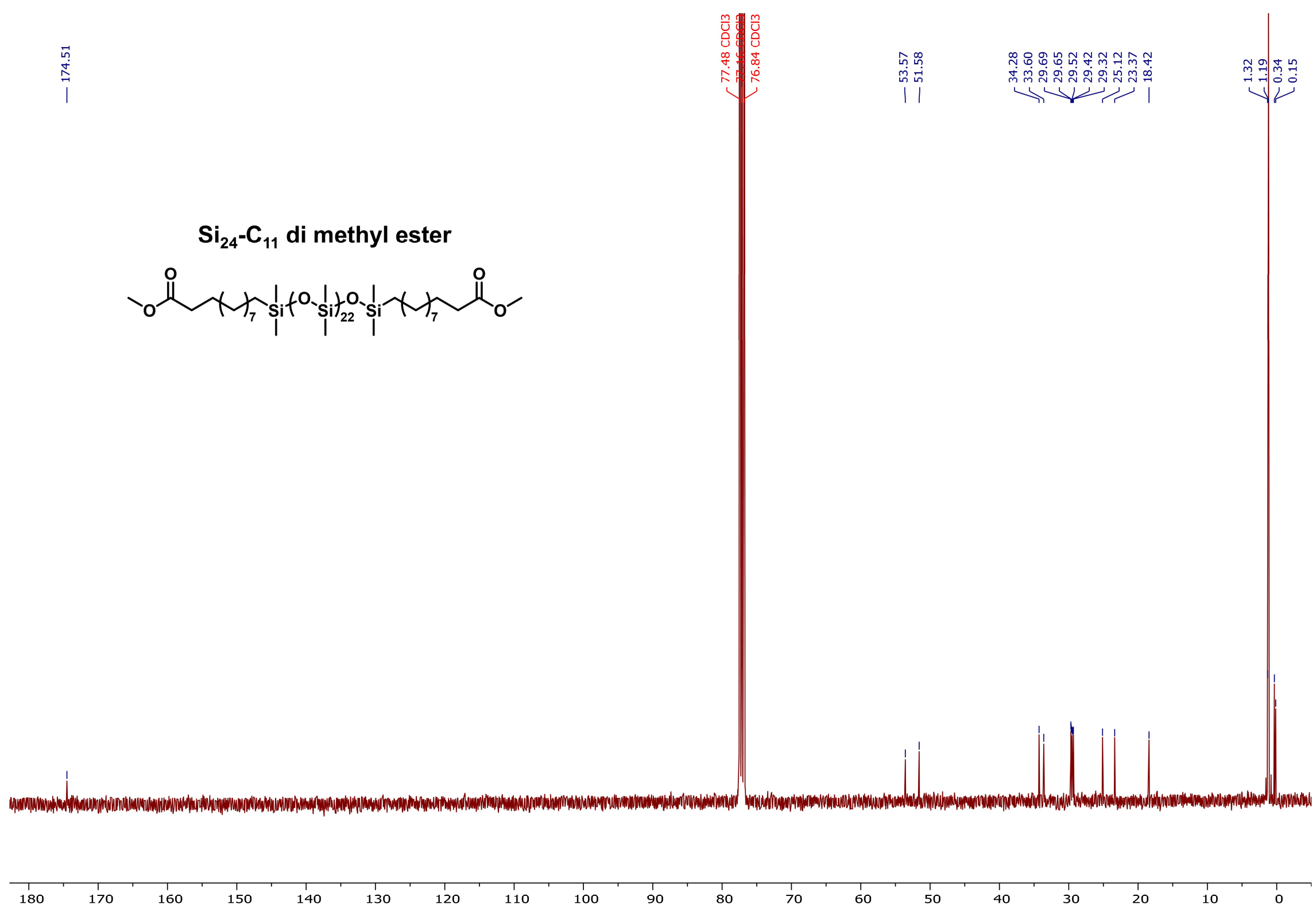

Figure S33: ^{13}C NMR spectrum of Si_{24}-C_{11} dimethyl ester in $CDCl_3$.

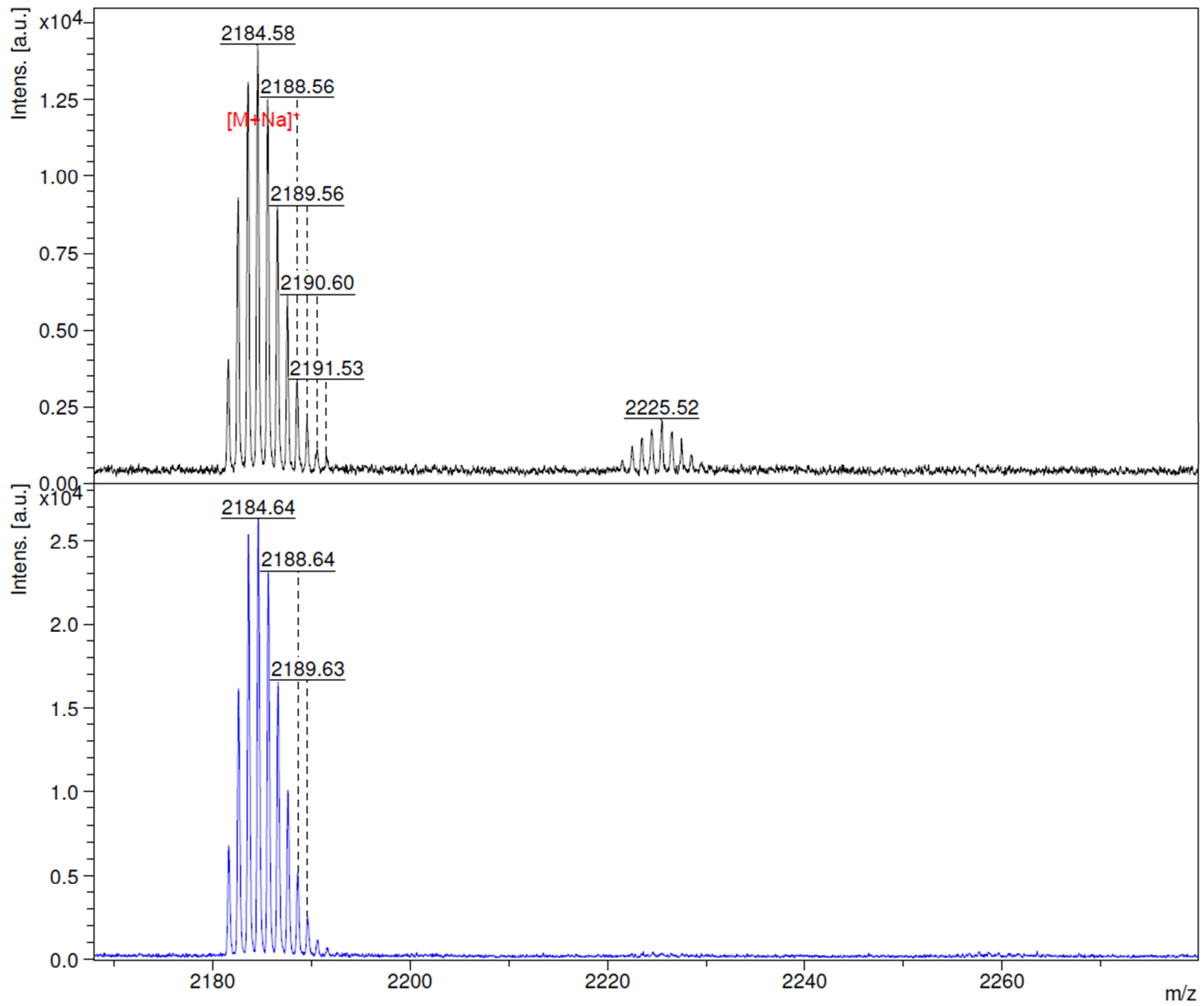

Figure S34: MALDI-ToF-MS spectra of Si_{24}-C_{11} dimethyl ester (black: CHCA, blue: DCTB matrix).

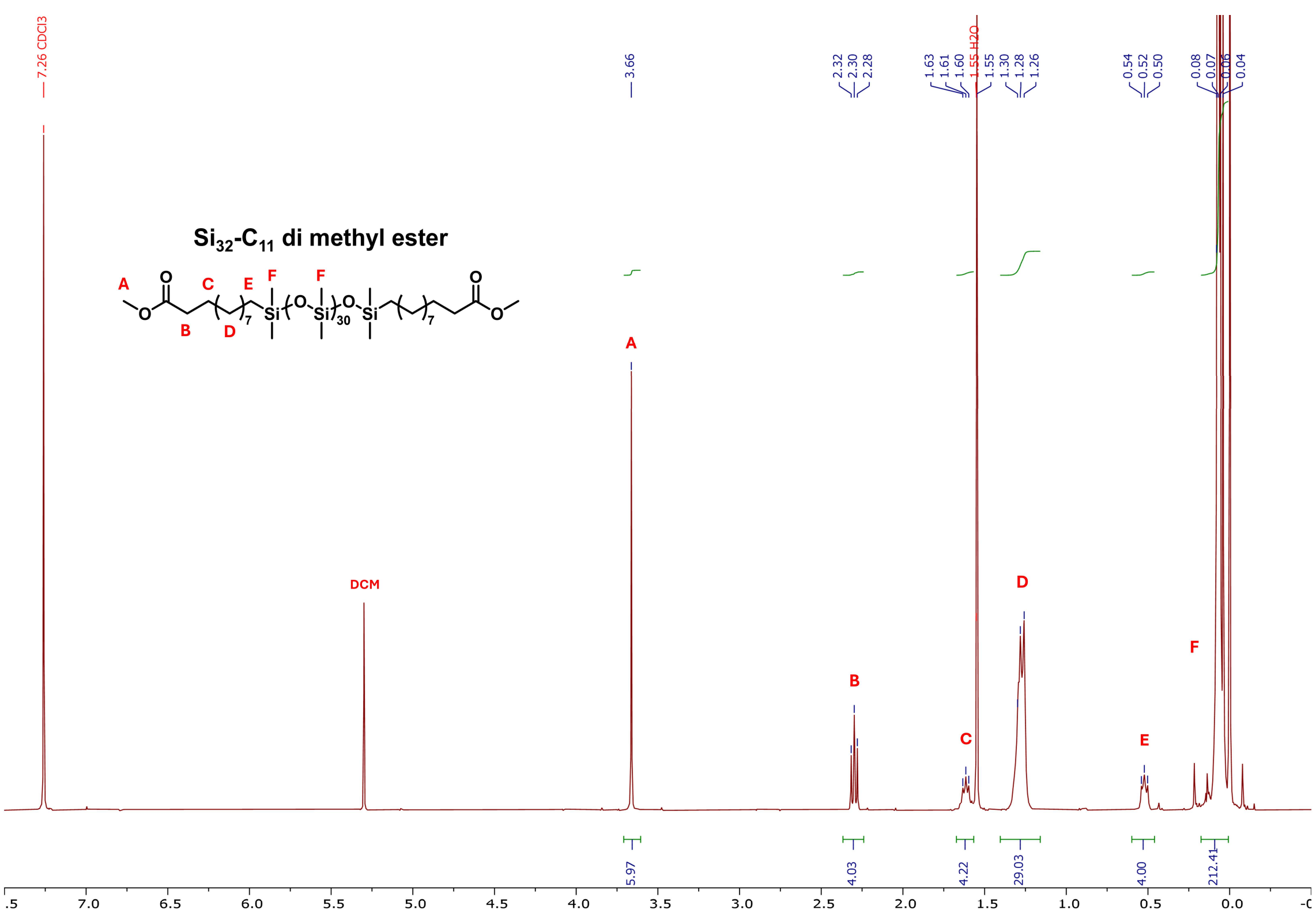

Figure S35: ^{1}H NMR spectrum of Si_{32}-C_{11} dimethyl ester in $CDCl_3$.

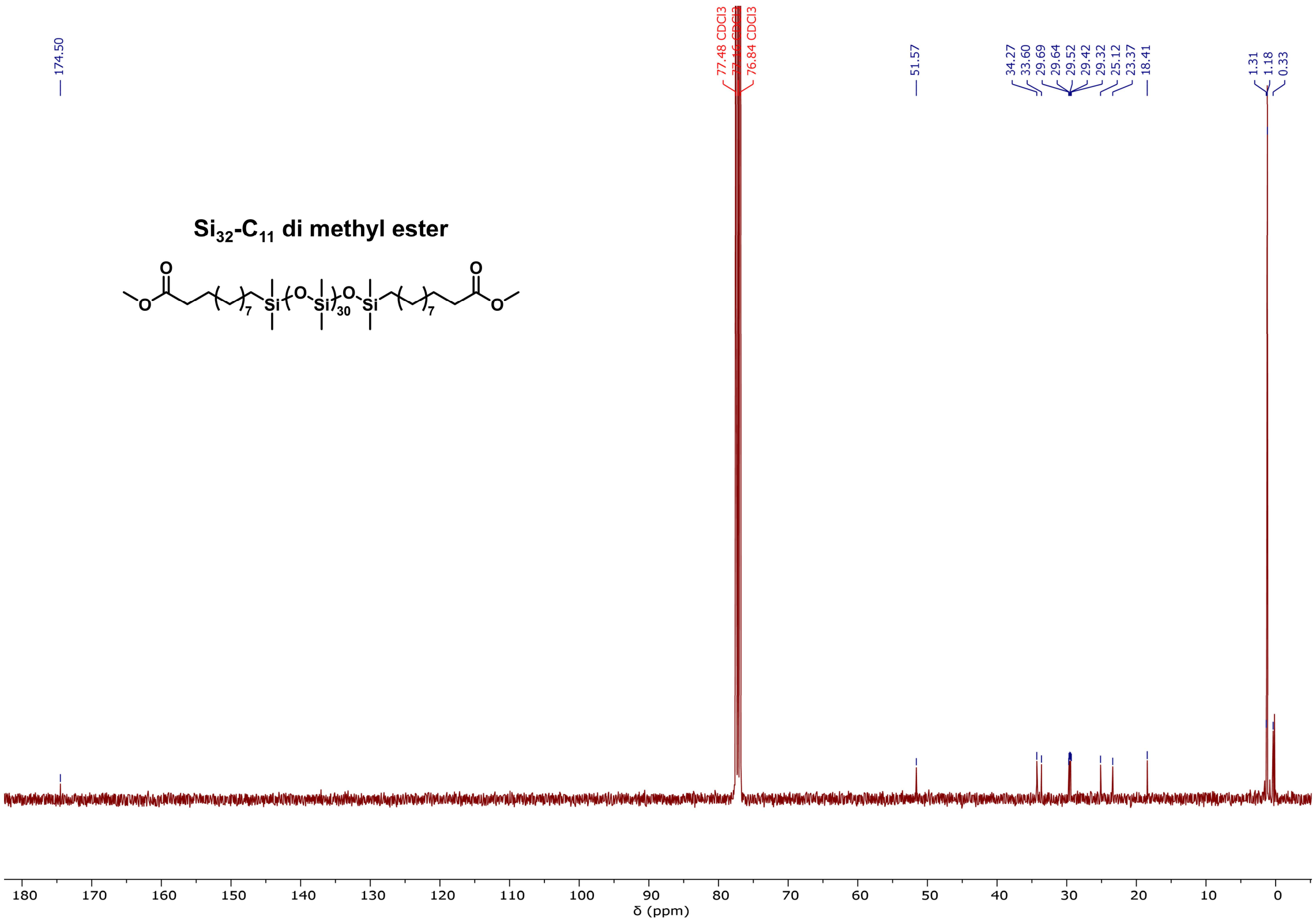

Figure S36: ^{13}C NMR spectrum of Si_{32}-C_{11} dimethyl ester in $CDCl_3$.

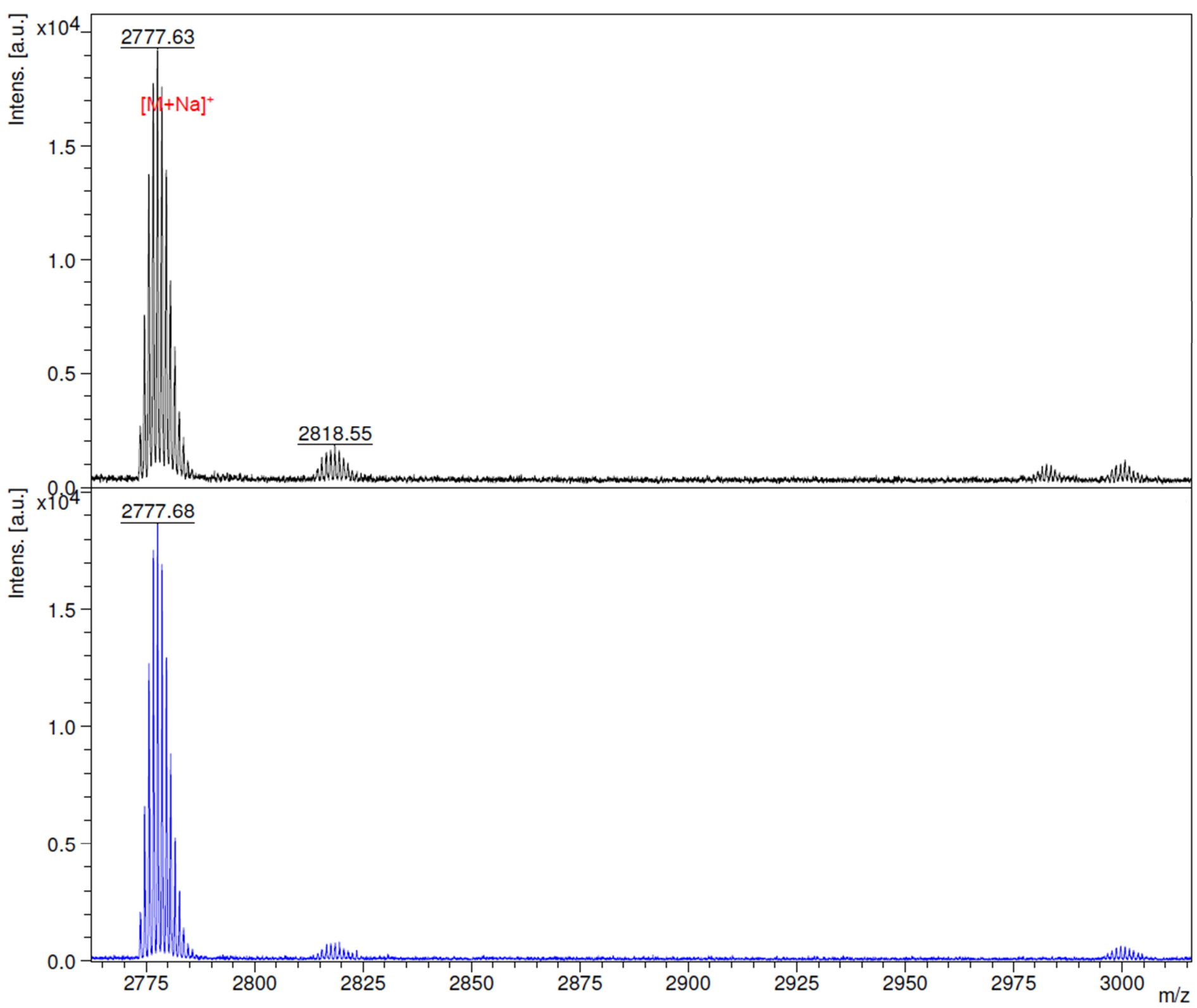

Figure S37: MALDI-ToF-MS spectra of Si_{32}-C_{11} dimethyl ester (black: CHCA, blue: DCTB matrix).

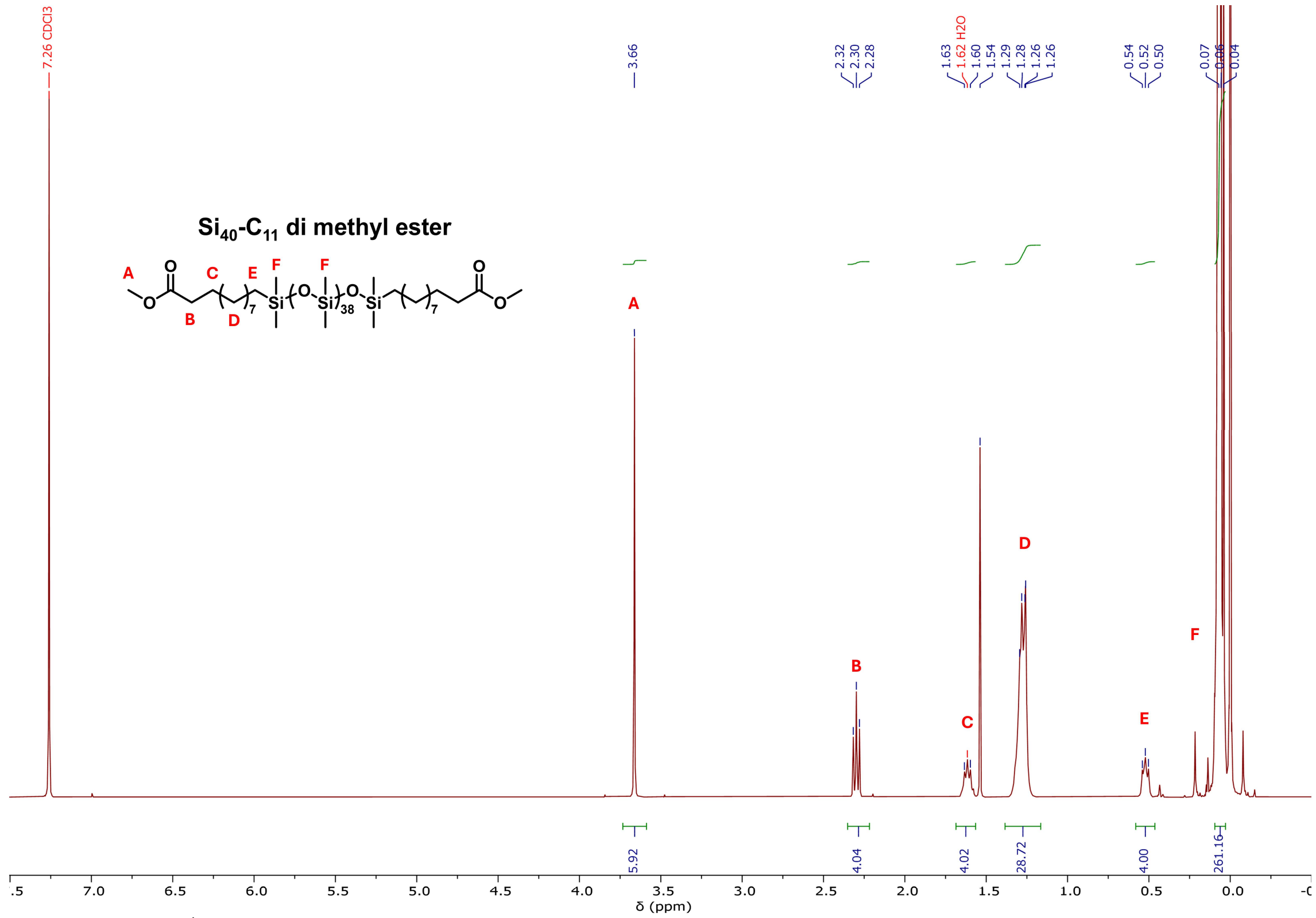

Figure S38: ^{1}H NMR spectrum of Si_{40}-C_{11} dimethyl ester in $CDCl_3$.

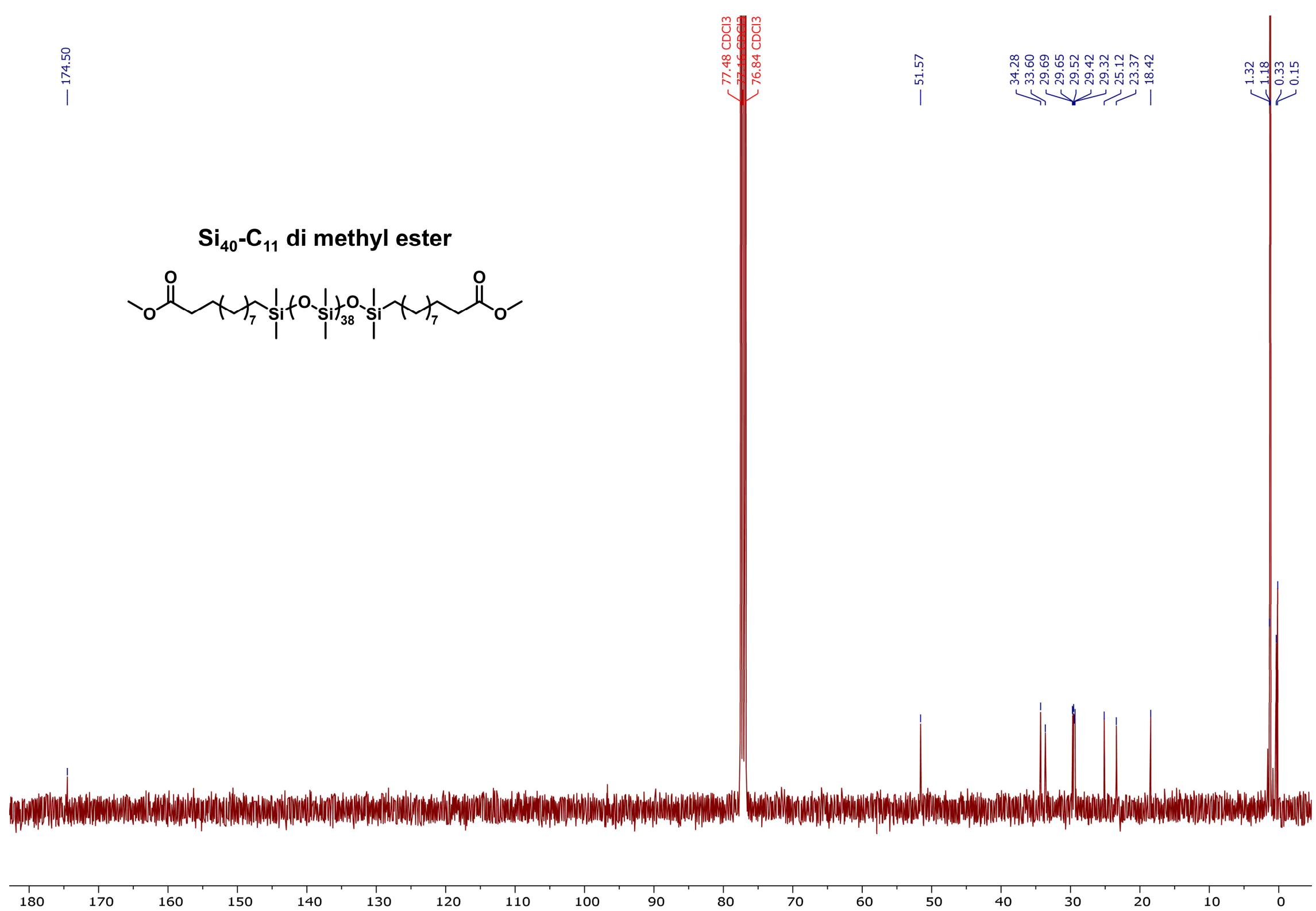

Figure S39: ^{13}C NMR spectrum of Si_{40}-C_{11} dimethyl ester in $CDCl_3$.

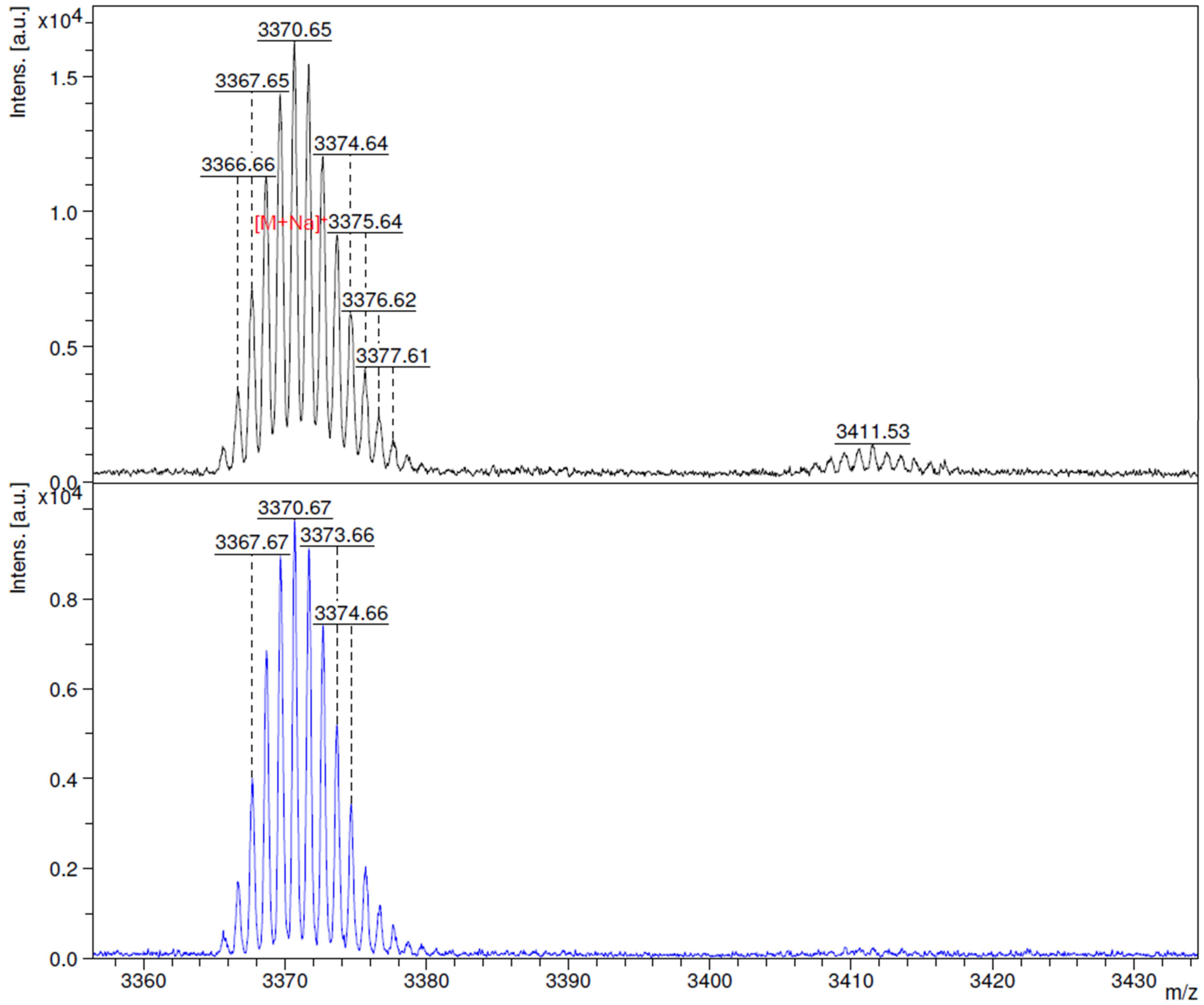

Figure S40: MALDI-ToF-MS spectra of Si_{40}-C_{11} dimethyl ester (black: CHCA, blue: DCTB matrix).

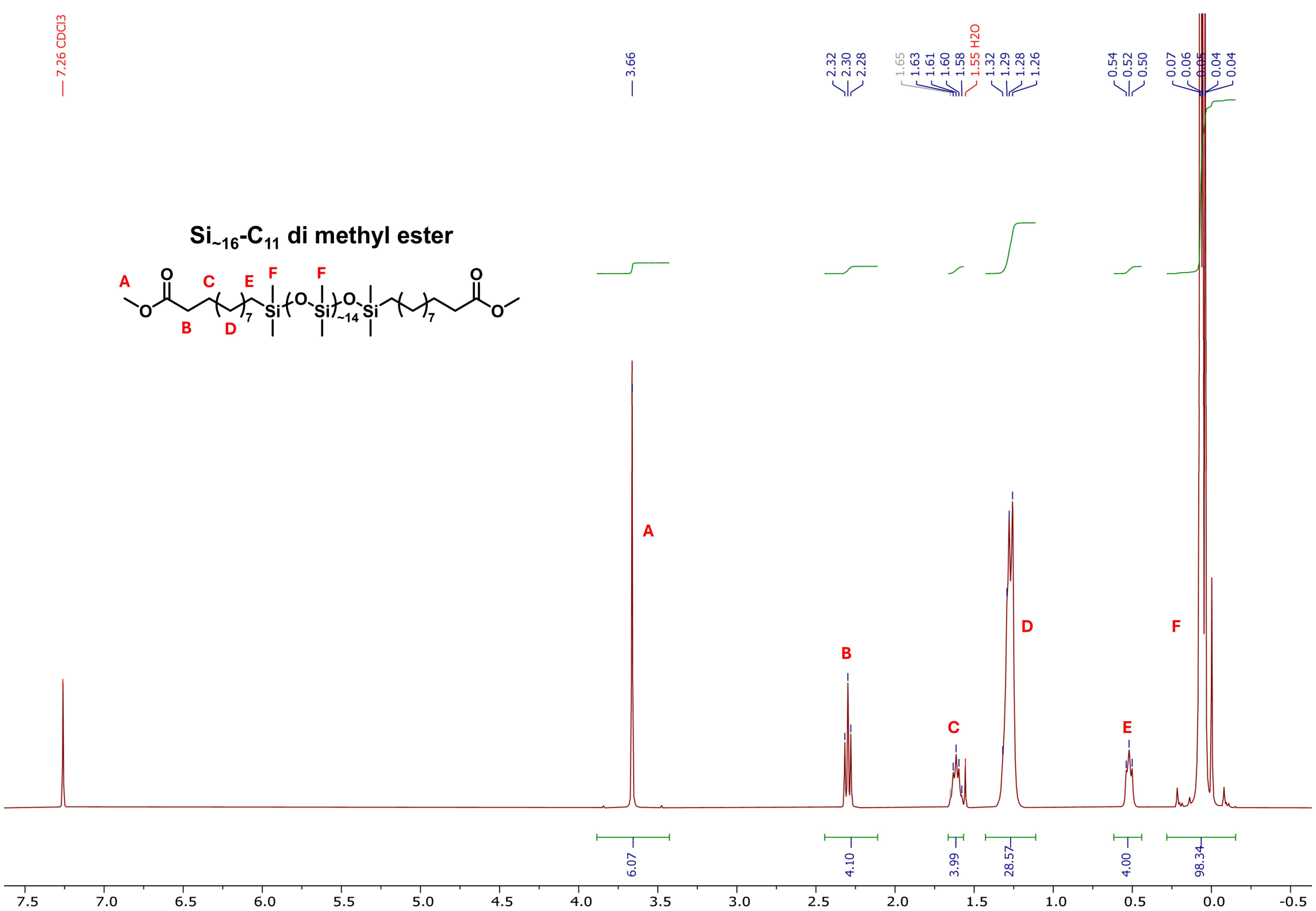

Figure S41: ^{1}H NMR spectrum of $Si_{\sim 16}$-C_{11} dimethyl ester in $CDCl_3$.

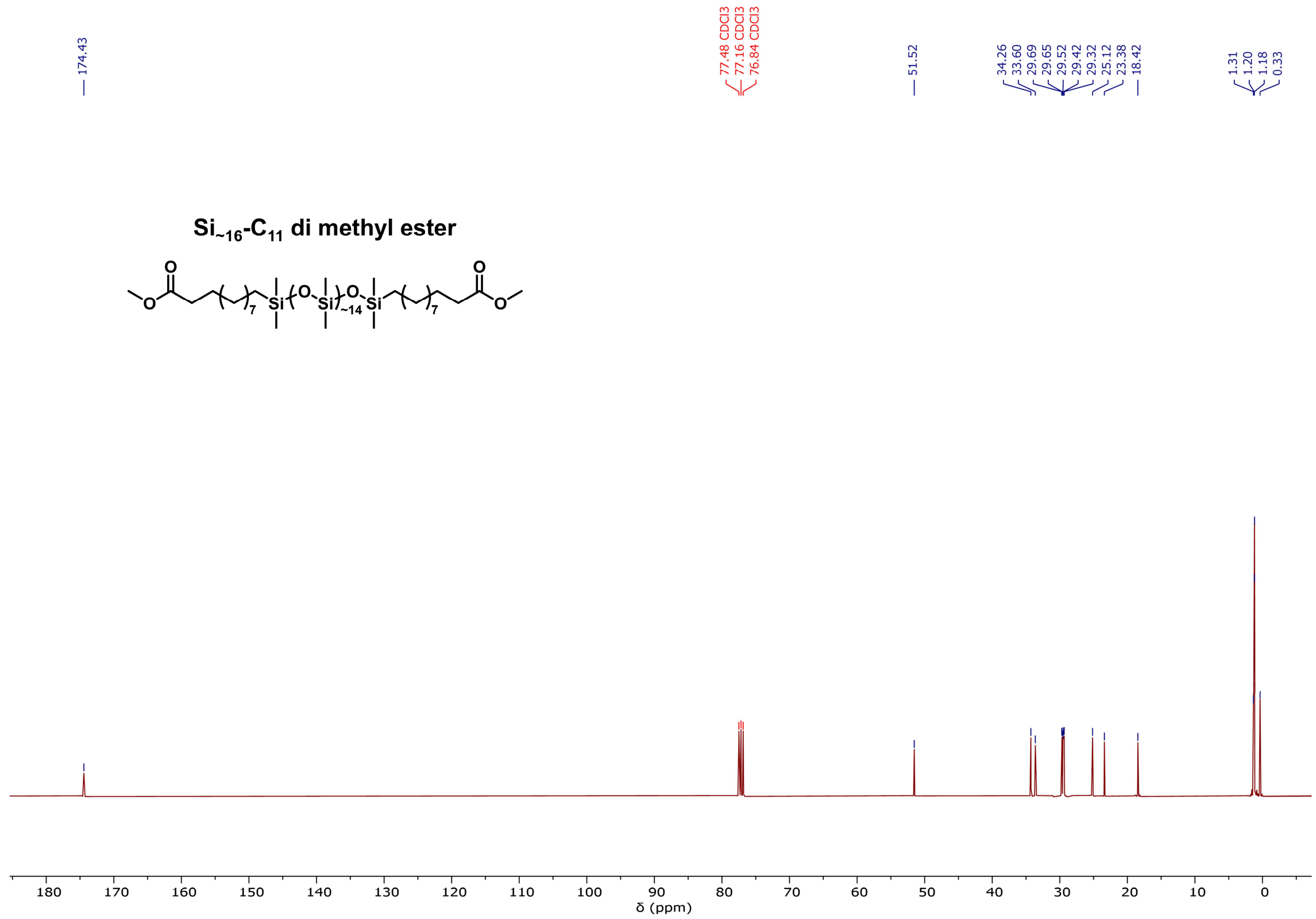

Figure S42: ^{13}C NMR spectrum of $Si_{\sim16}$-C_{11} dimethyl ester in $CDCl_3$.

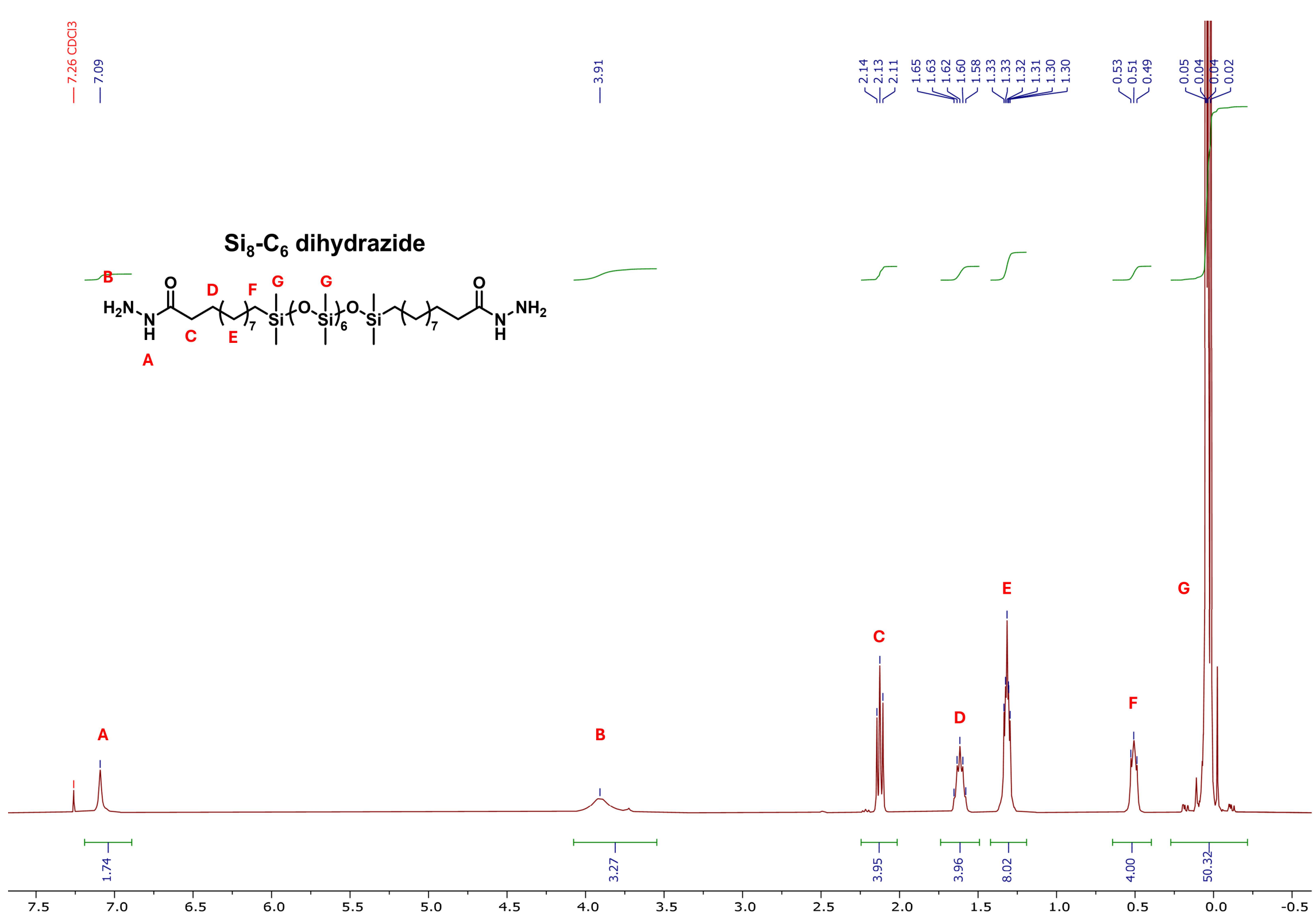

Figure S43: ^{1}H NMR spectrum of Si_8-C_6 dihydrazide in $CDCl_3$.

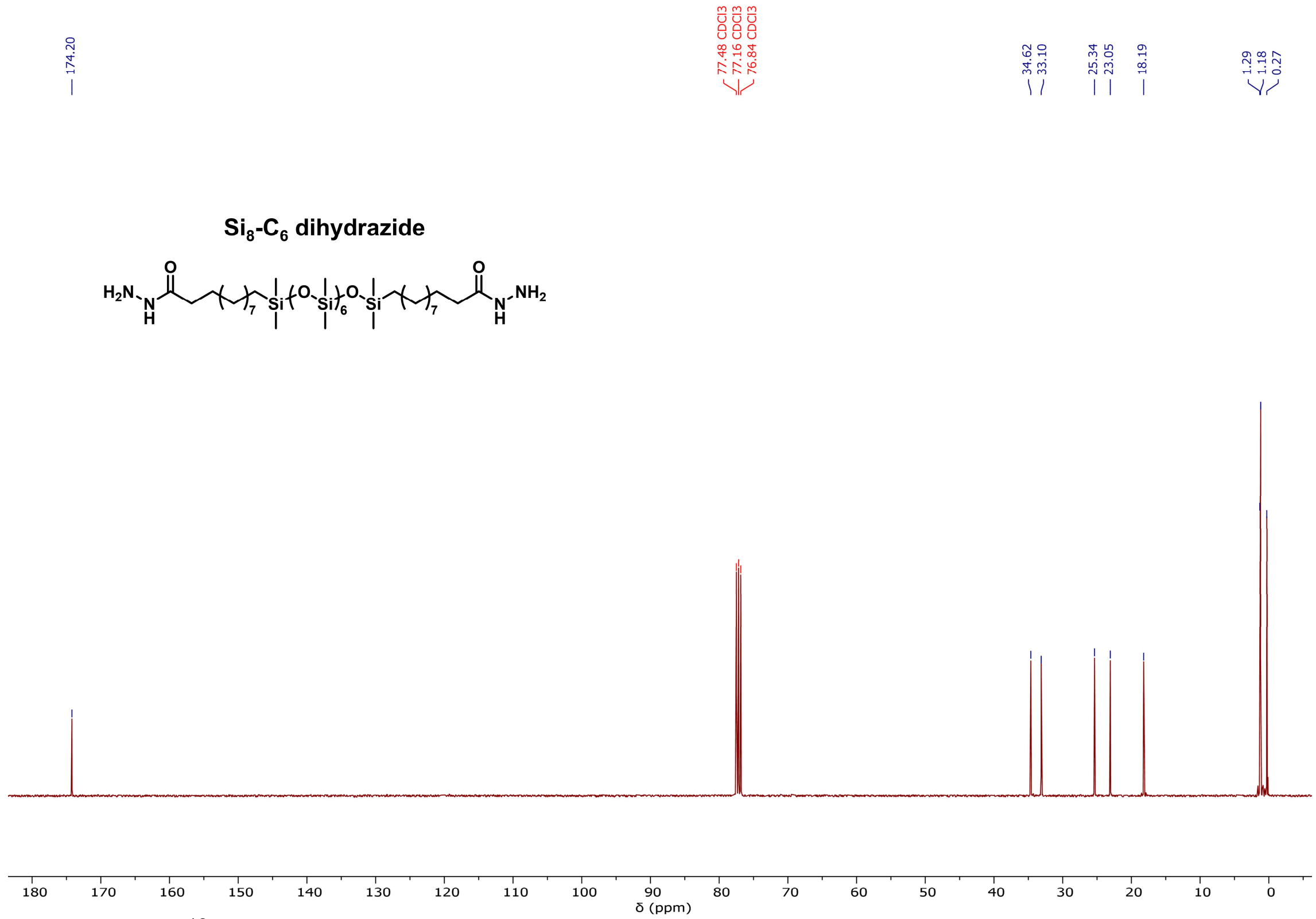

Figure S44: ^{13}C NMR spectrum of Si_8-C_6 dihydrazide in $CDCl_3$.

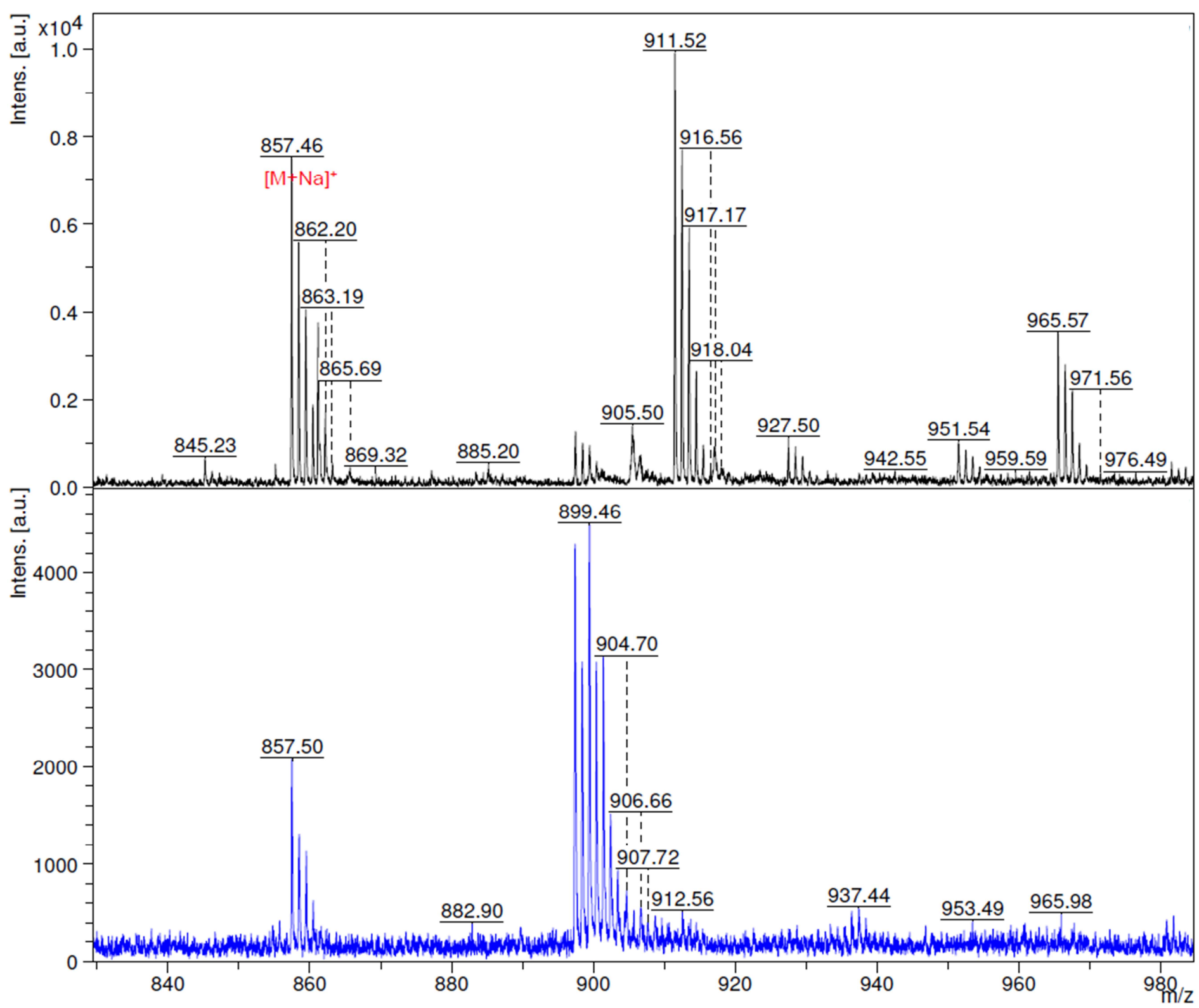

Figure S45: MALDI-ToF-MS spectra of Si_8-C_6 dihydrazide (black: CHCA, blue: DCTB matrix).

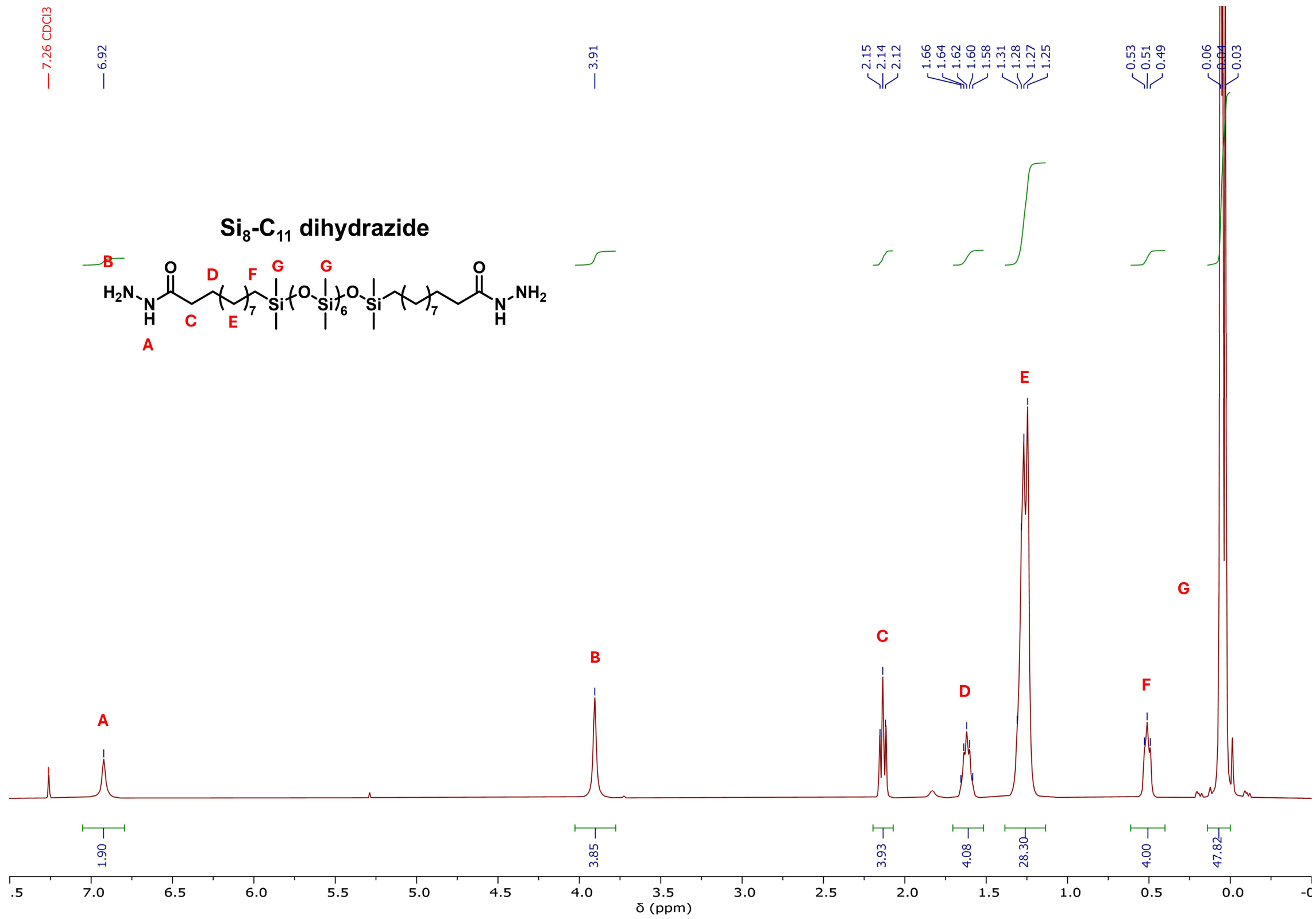

Figure S46: ^{1}H NMR spectrum of Si$_8$-C$_{11}$ dihydrazide in $CDCl_3$.

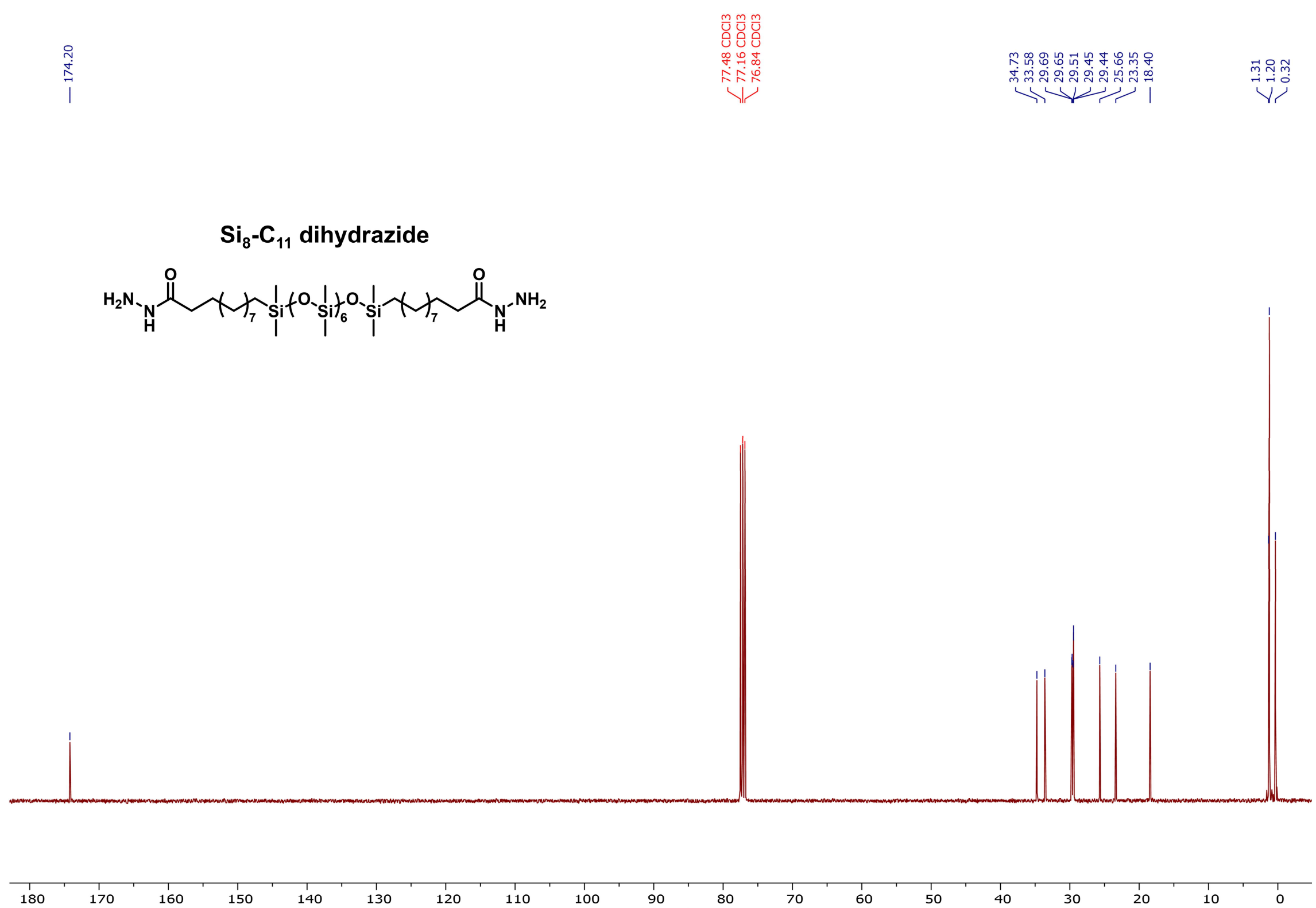

Figure S47: ^{13}C NMR spectrum of Si$_8$-C$_{11}$ dihydrazide in $CDCl_3$.

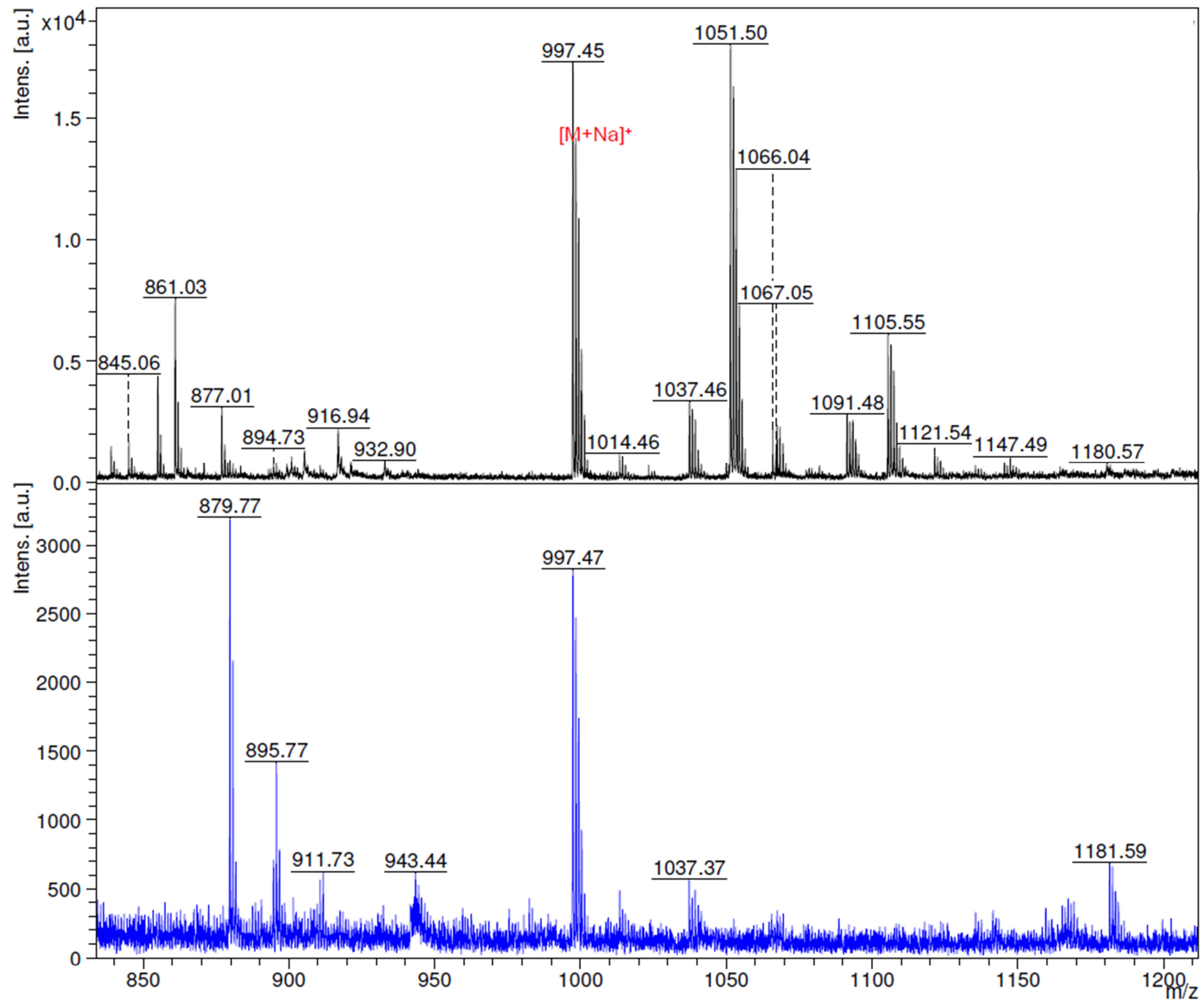

Figure S48: MALDI-ToF-MS spectra of Si_8-C_{11} dihydrazide (black: CHCA, blue: DCTB matrix).

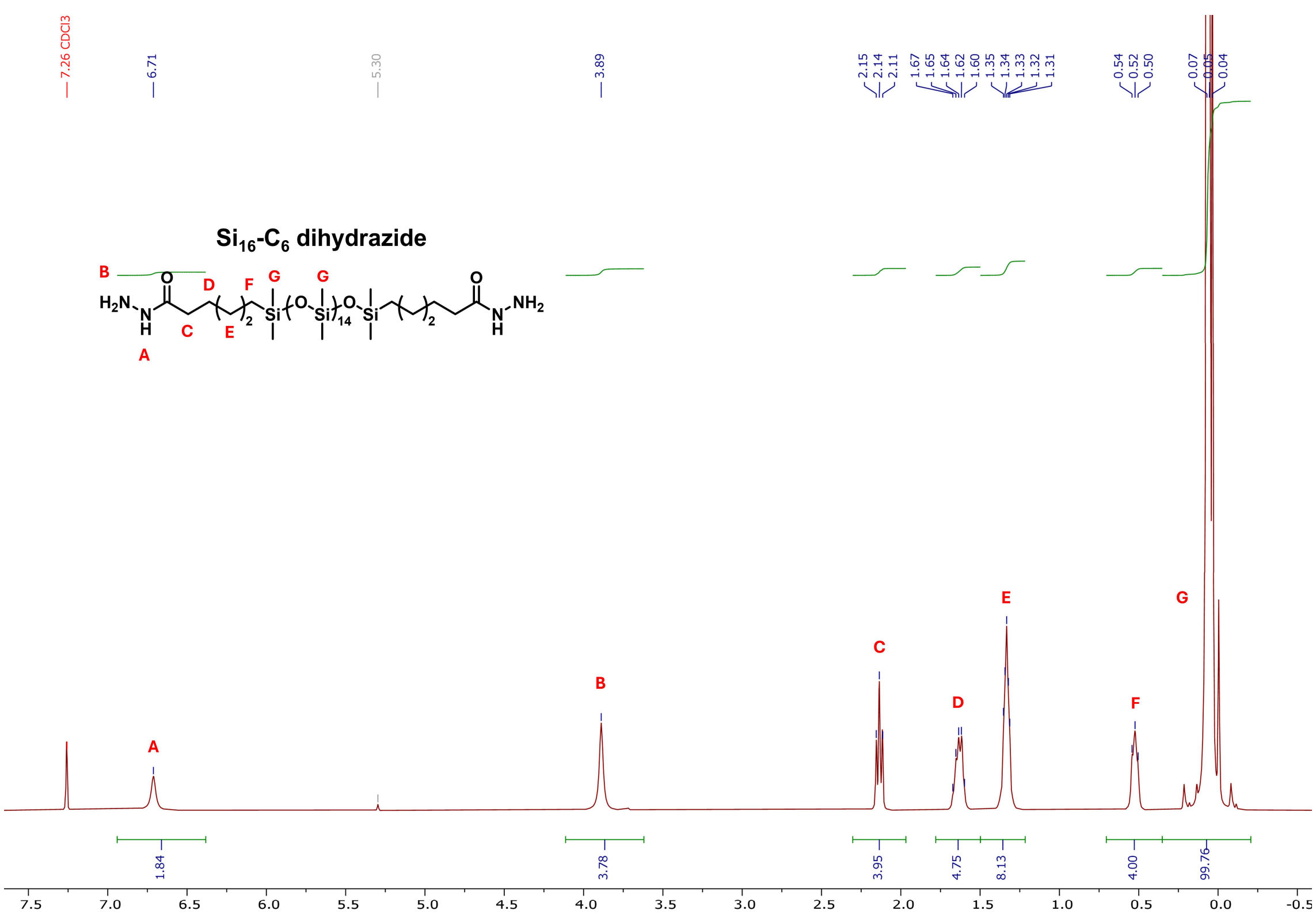

Figure S49: ^{1}H NMR spectrum of Si_{16}-C_6 dihydrazide in $CDCl_3$.

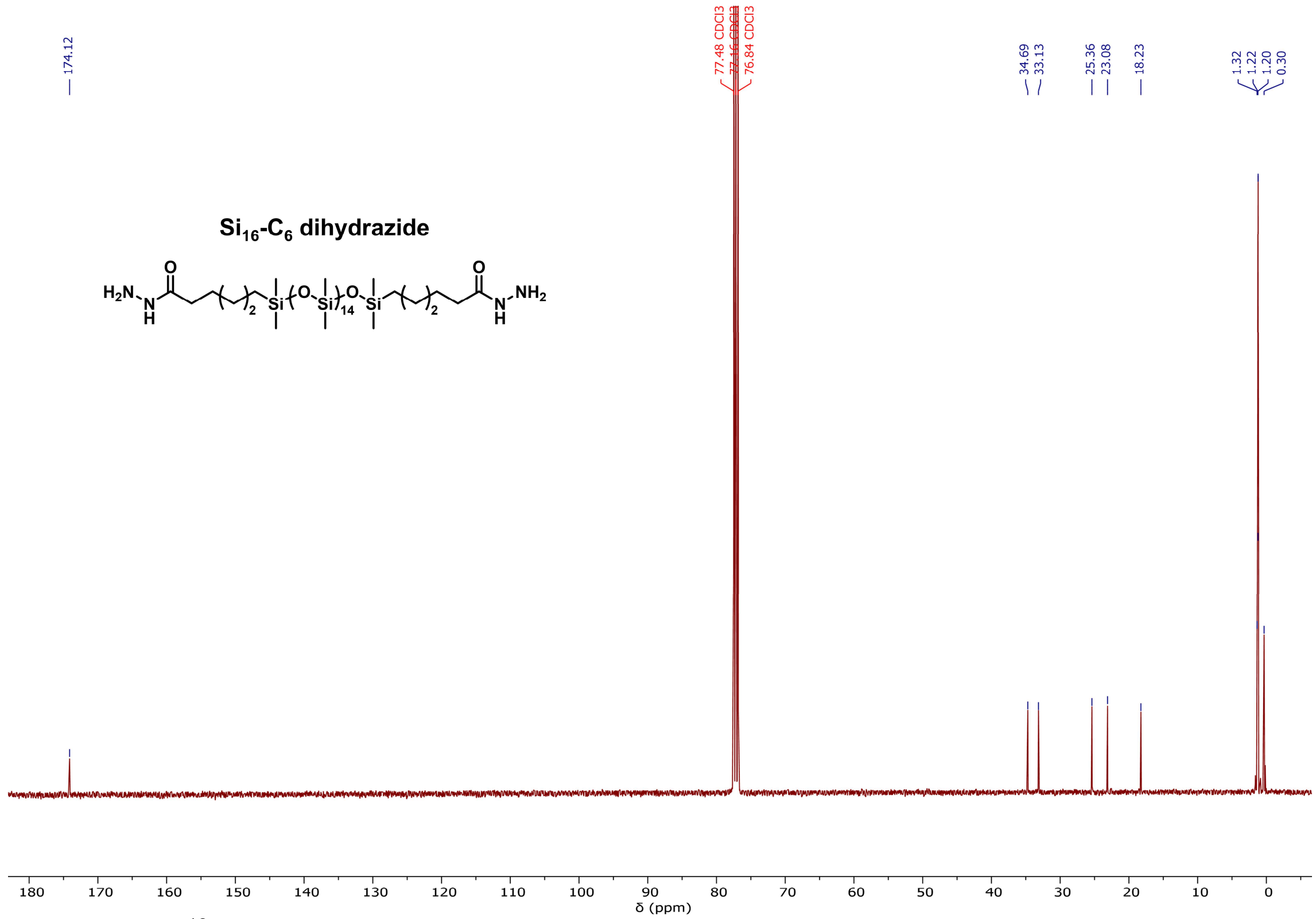

Figure S50: ^{13}C NMR spectrum of Si_{16}-C_6 dihydrazide in $CDCl_3$.

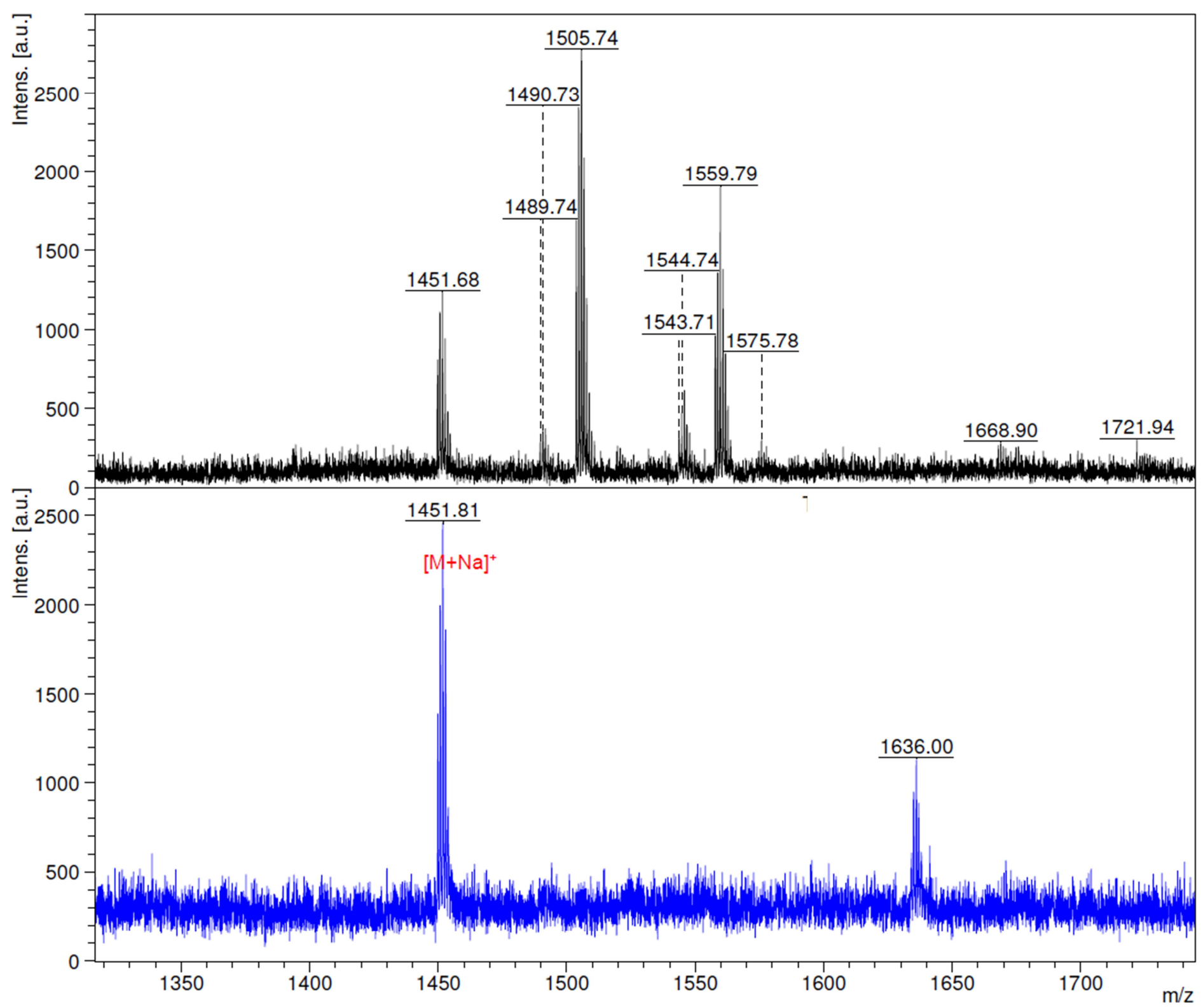

Figure S51: MALDI-ToF-MS spectra of Si_{16}-C_6 dihydrazide (black: CHCA, blue: DCTB matrix).

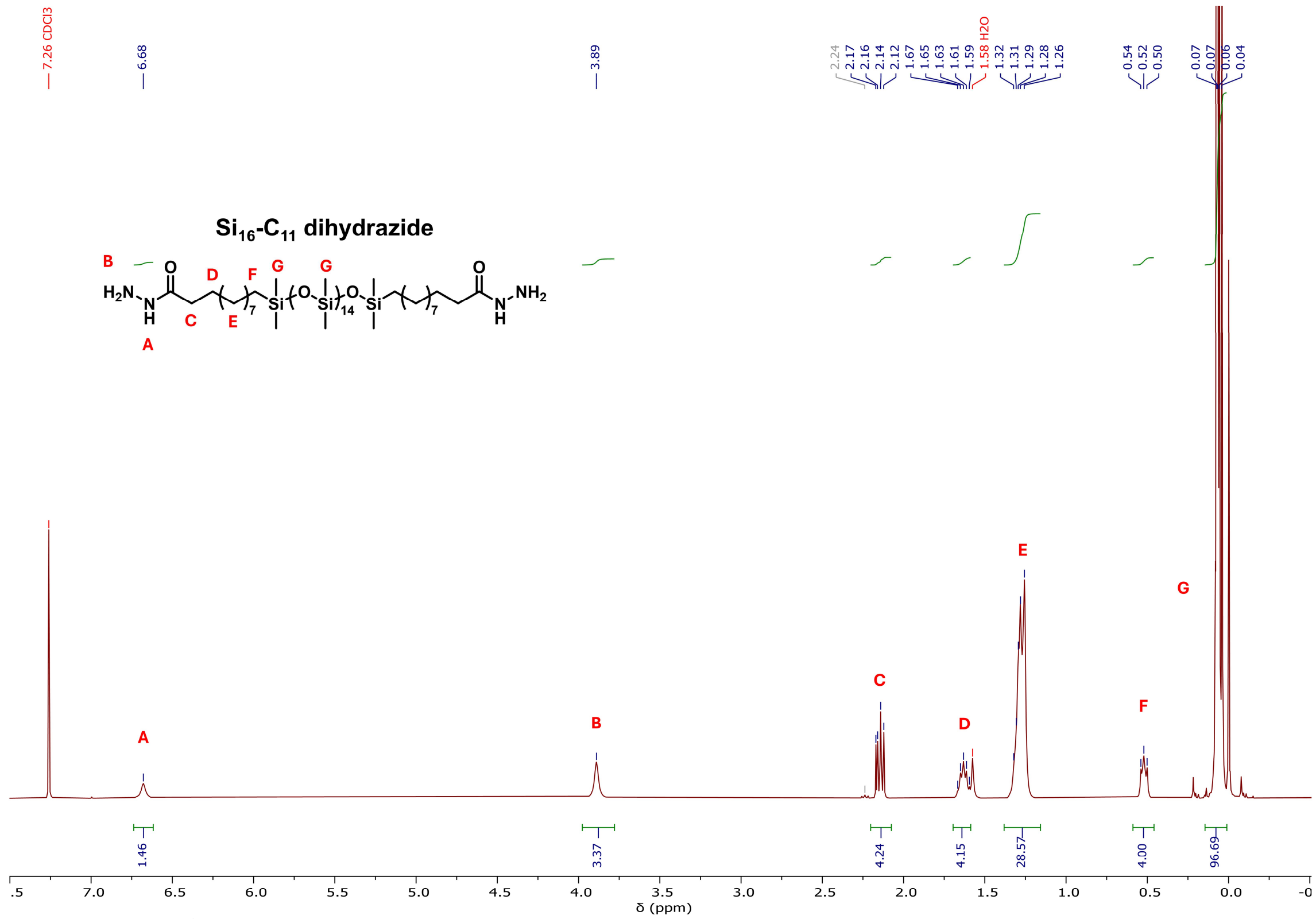

Figure S52: ^{1}H NMR spectrum of Si_{16}-C_{11} dihydrazide in $CDCl_3$.

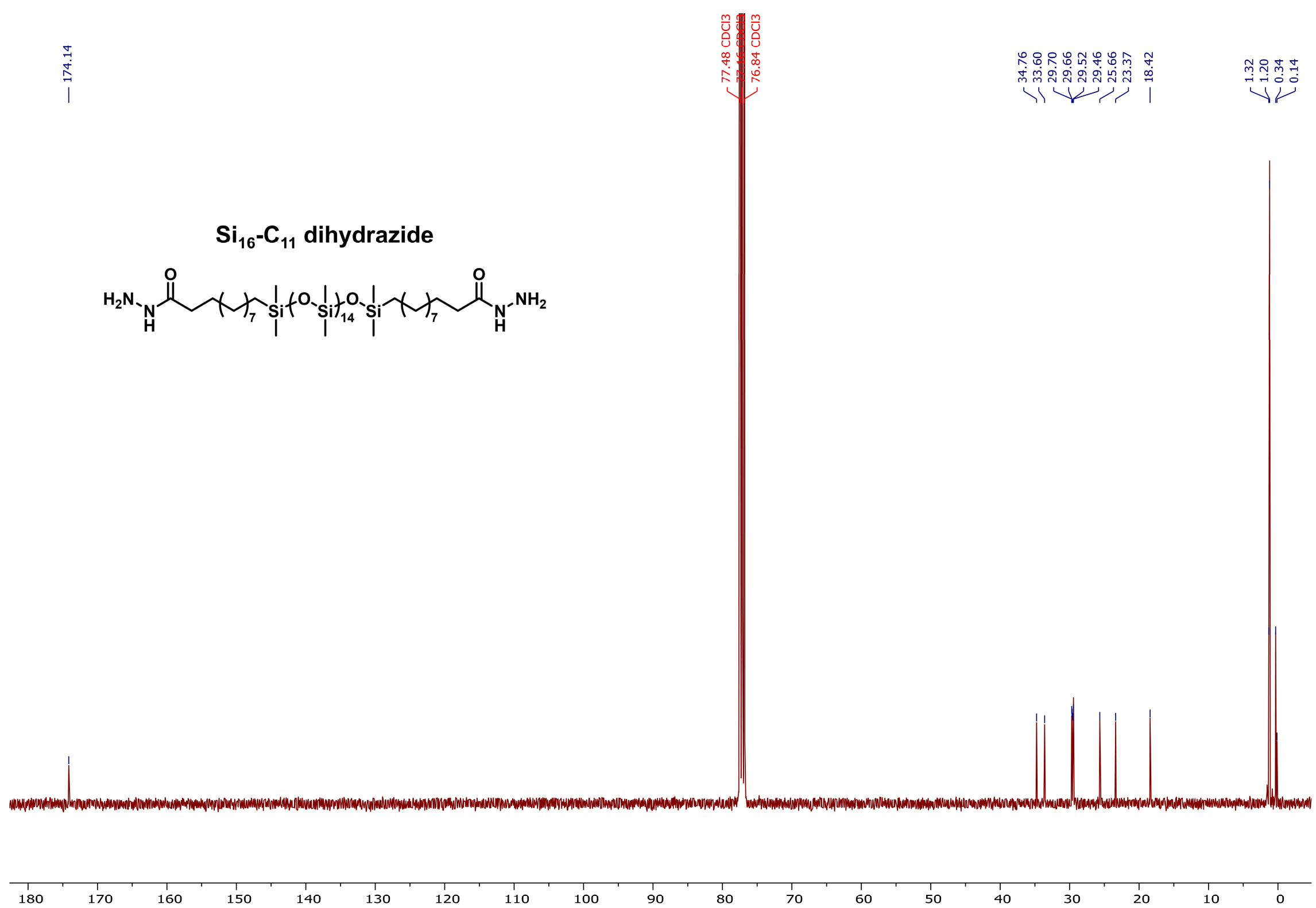

Figure S53: ^{13}C NMR spectrum of Si_{16}-C_{11} dihydrazide in $CDCl_3$.

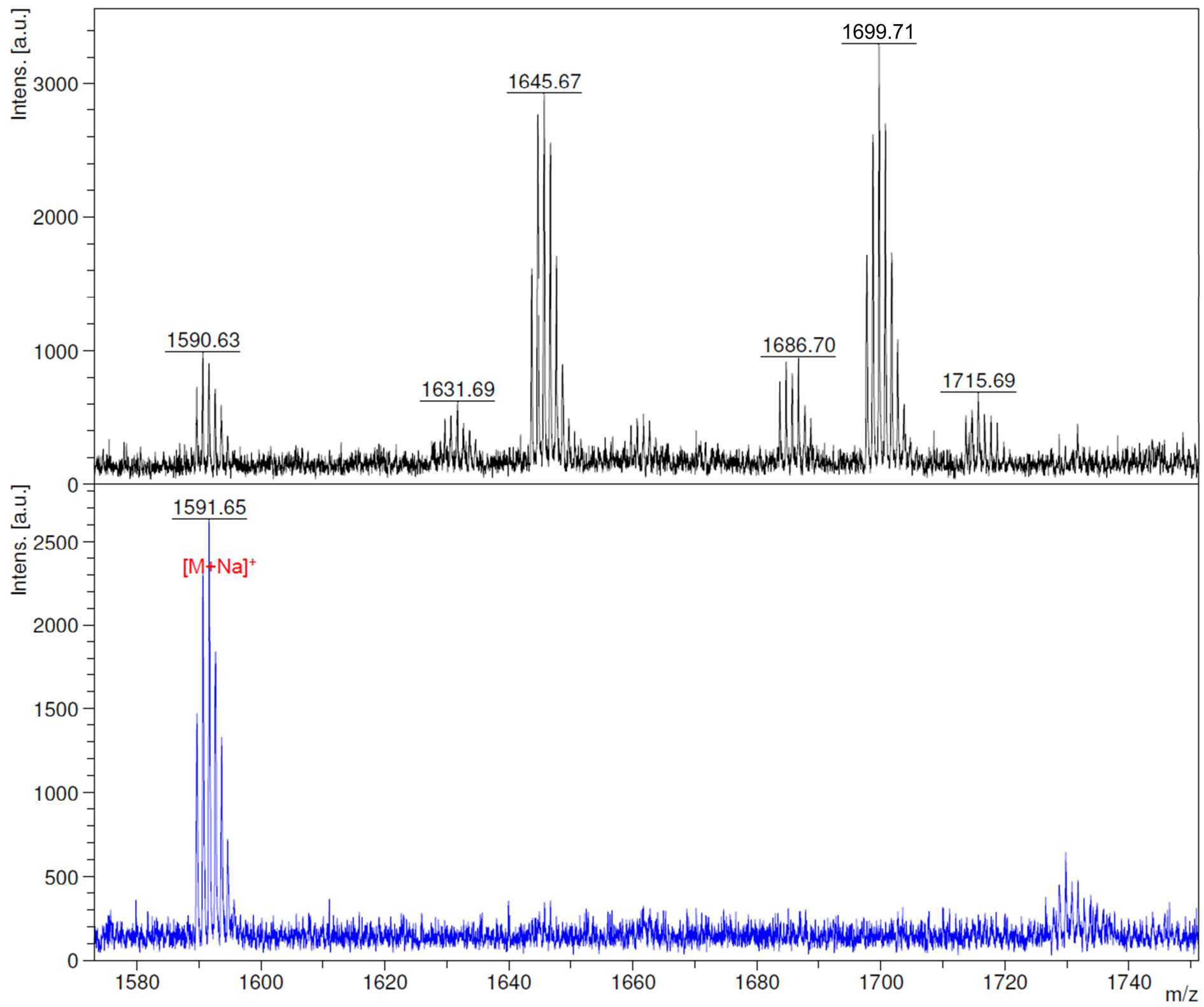

Figure S54: MALDI-ToF-MS spectra of Si_{16}-C_{11} dihydrazide (black: CHCA, blue: DCTB matrix).

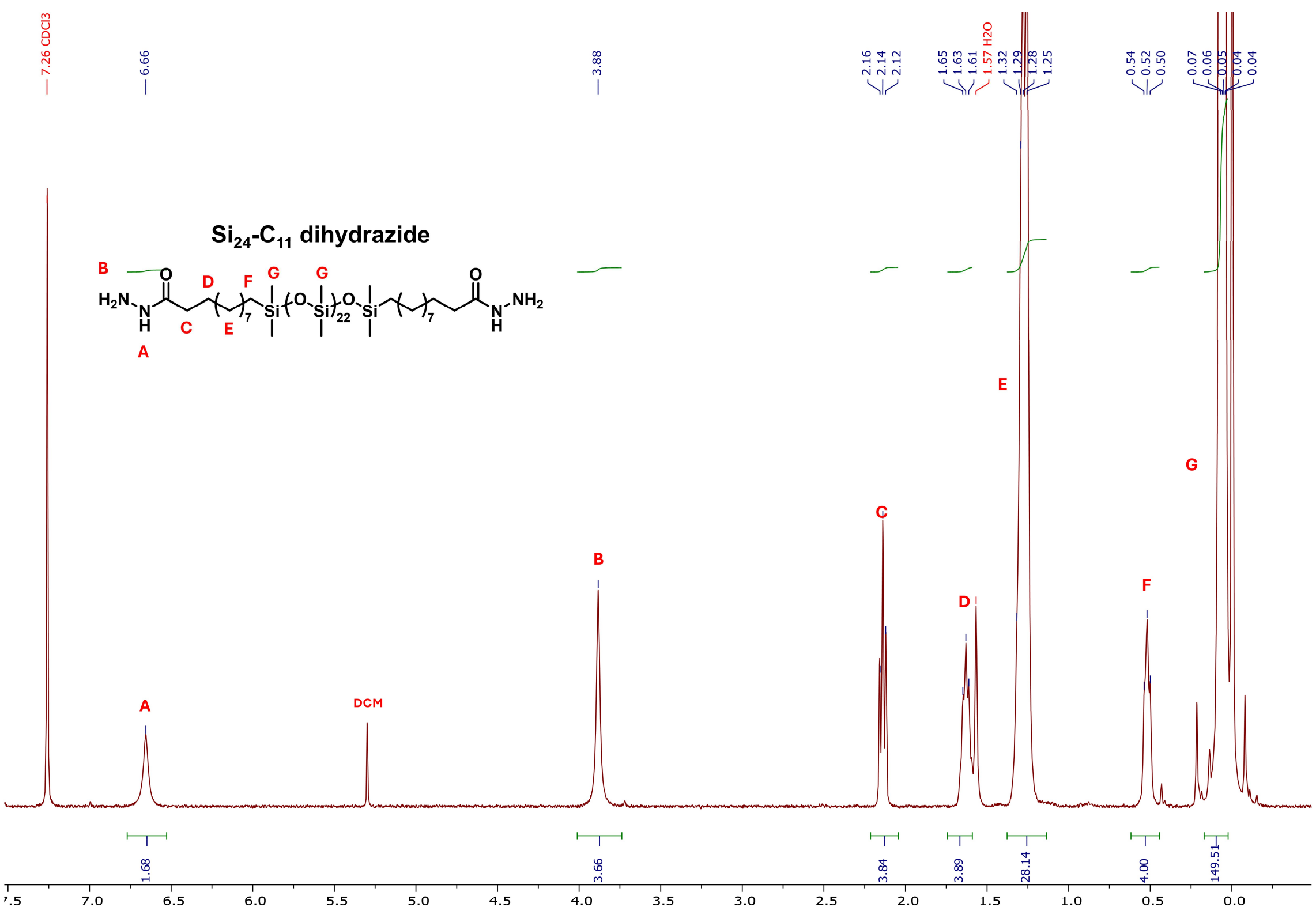

Figure S55: ^{1}H NMR spectrum of Si_{24}-C_{11} dihydrazide in $CDCl_3$.

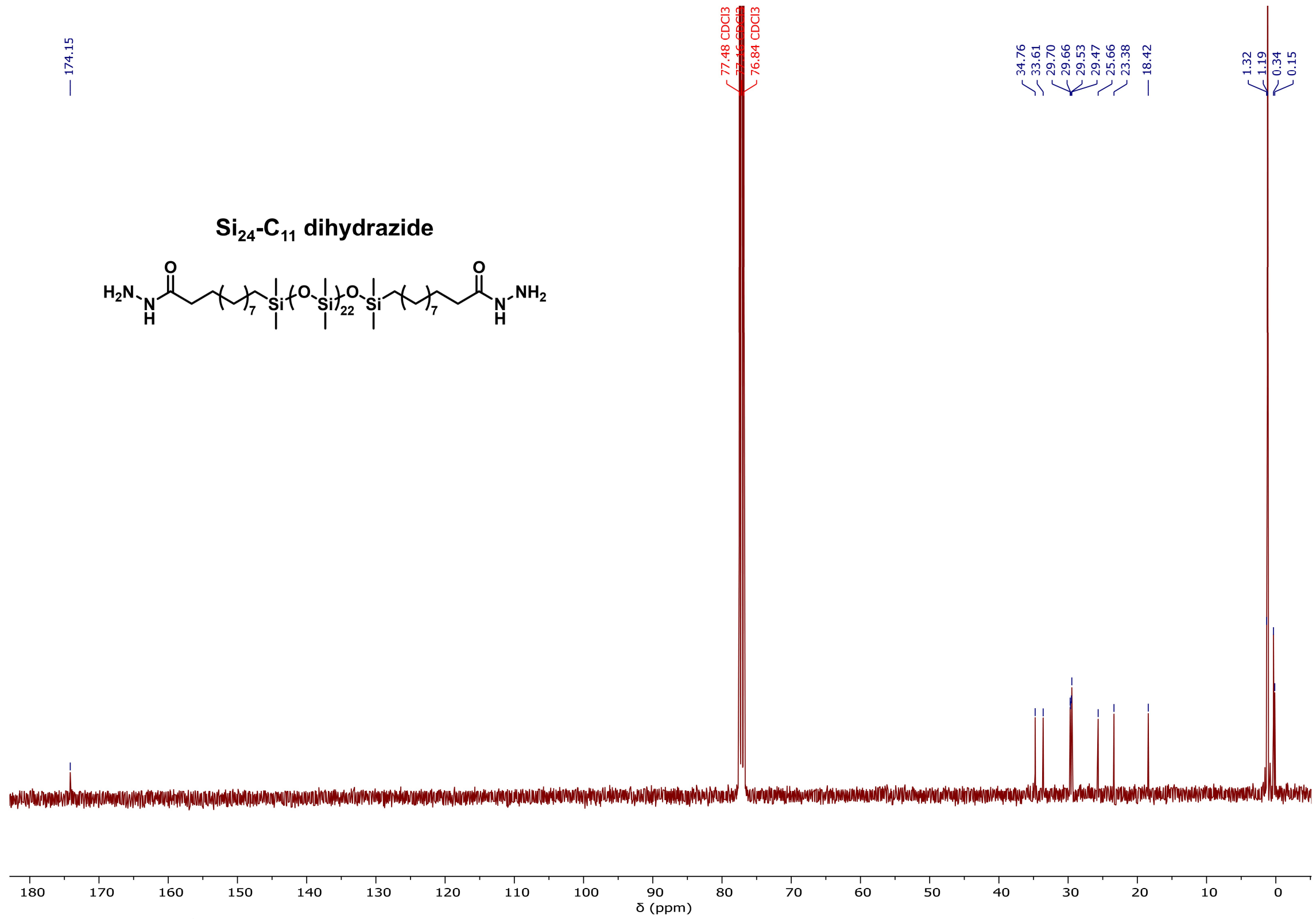

Figure S56: ^{13}C NMR spectrum of Si_{24}-C_{11} dihydrazide in $CDCl_3$.

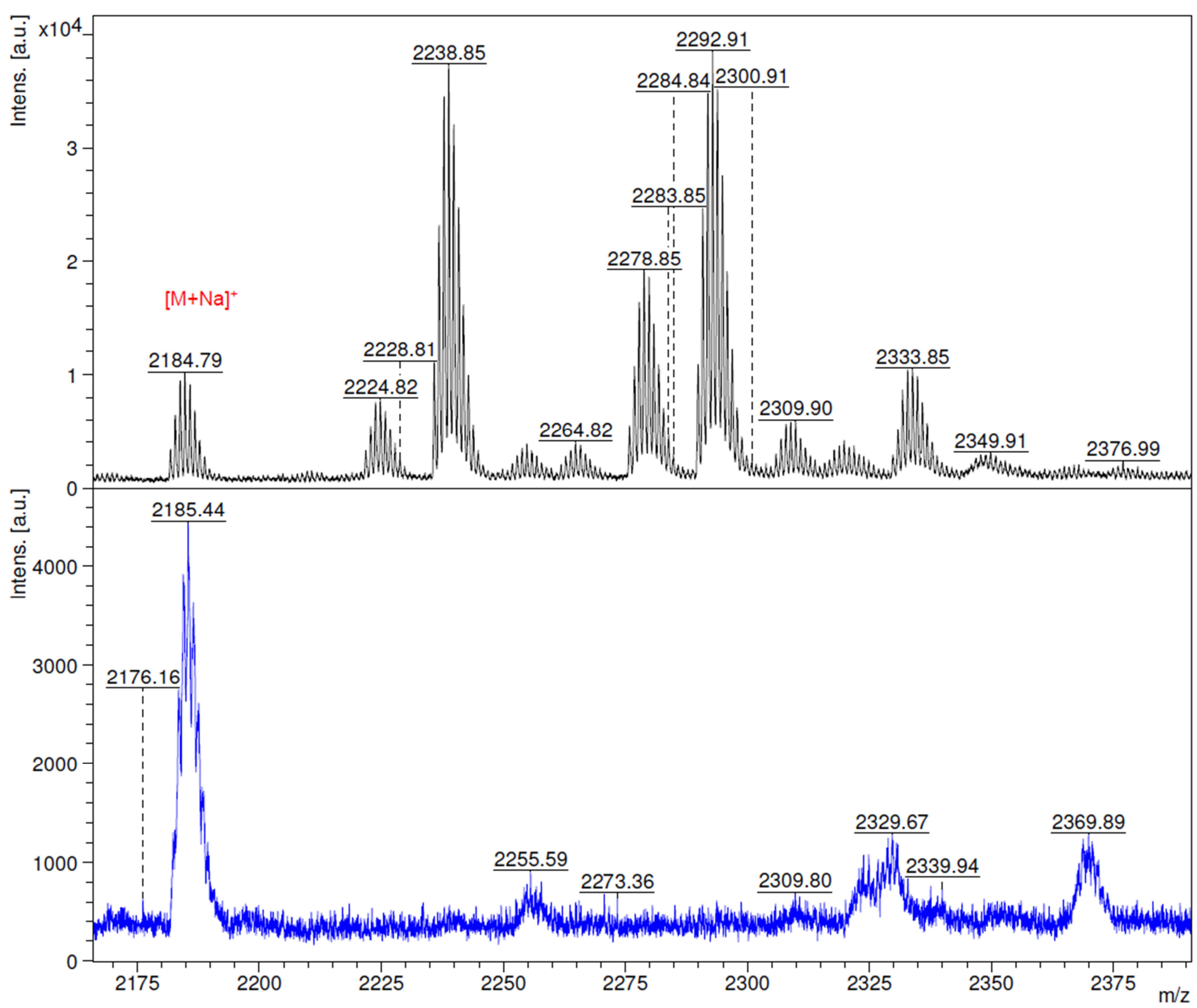

Figure S57: MALDI-ToF-MS spectra of Si_{24}-C_{11} dihydrazide (black: CHCA, blue: DCTB matrix).

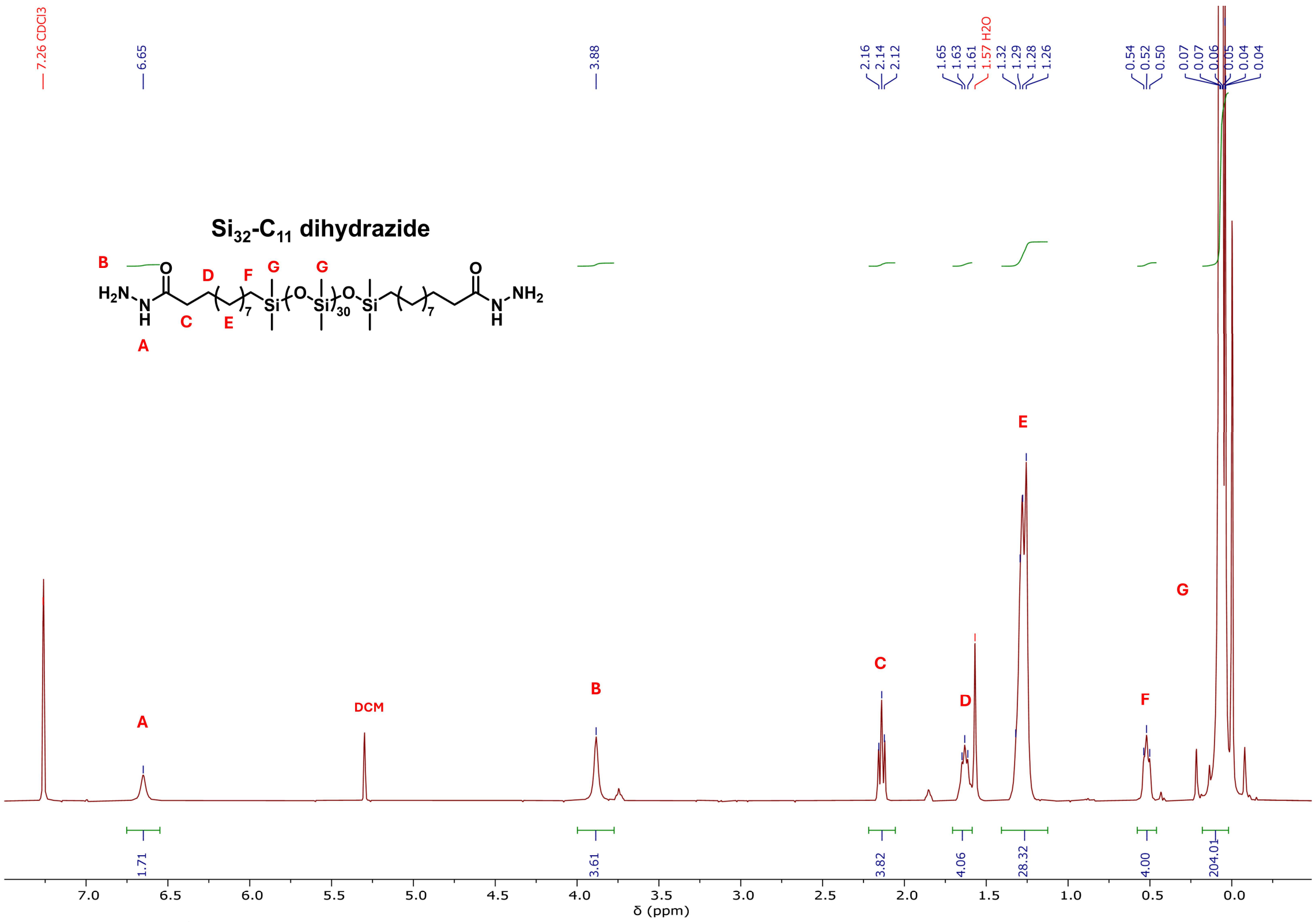

Figure S58: ^{1}H NMR spectrum of Si_{32}-C_{11} dihydrazide in $CDCl_3$.

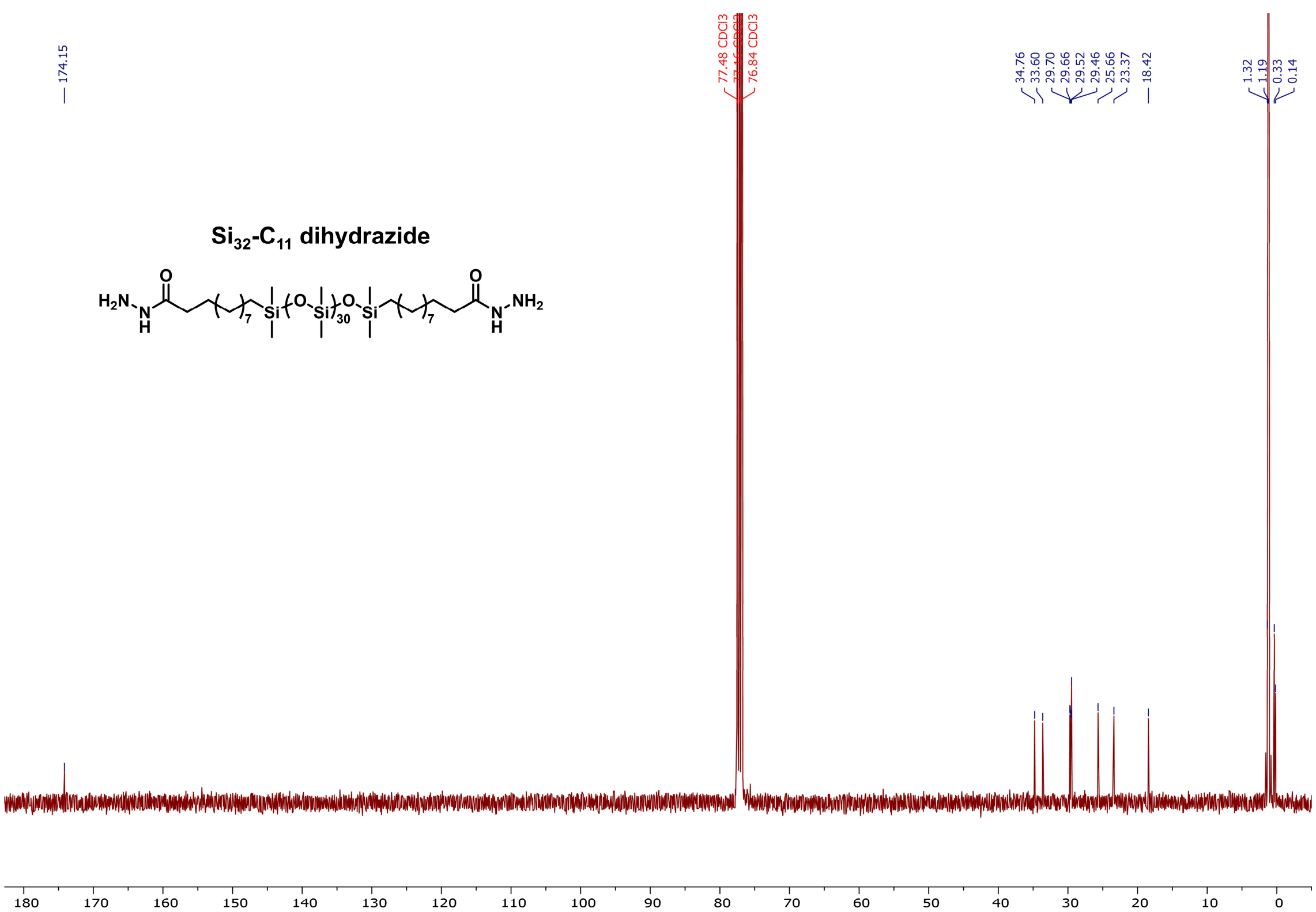

Figure S59: ^{13}C NMR spectrum of Si_{32}-C_{11} dihydrazide in $CDCl_3$.

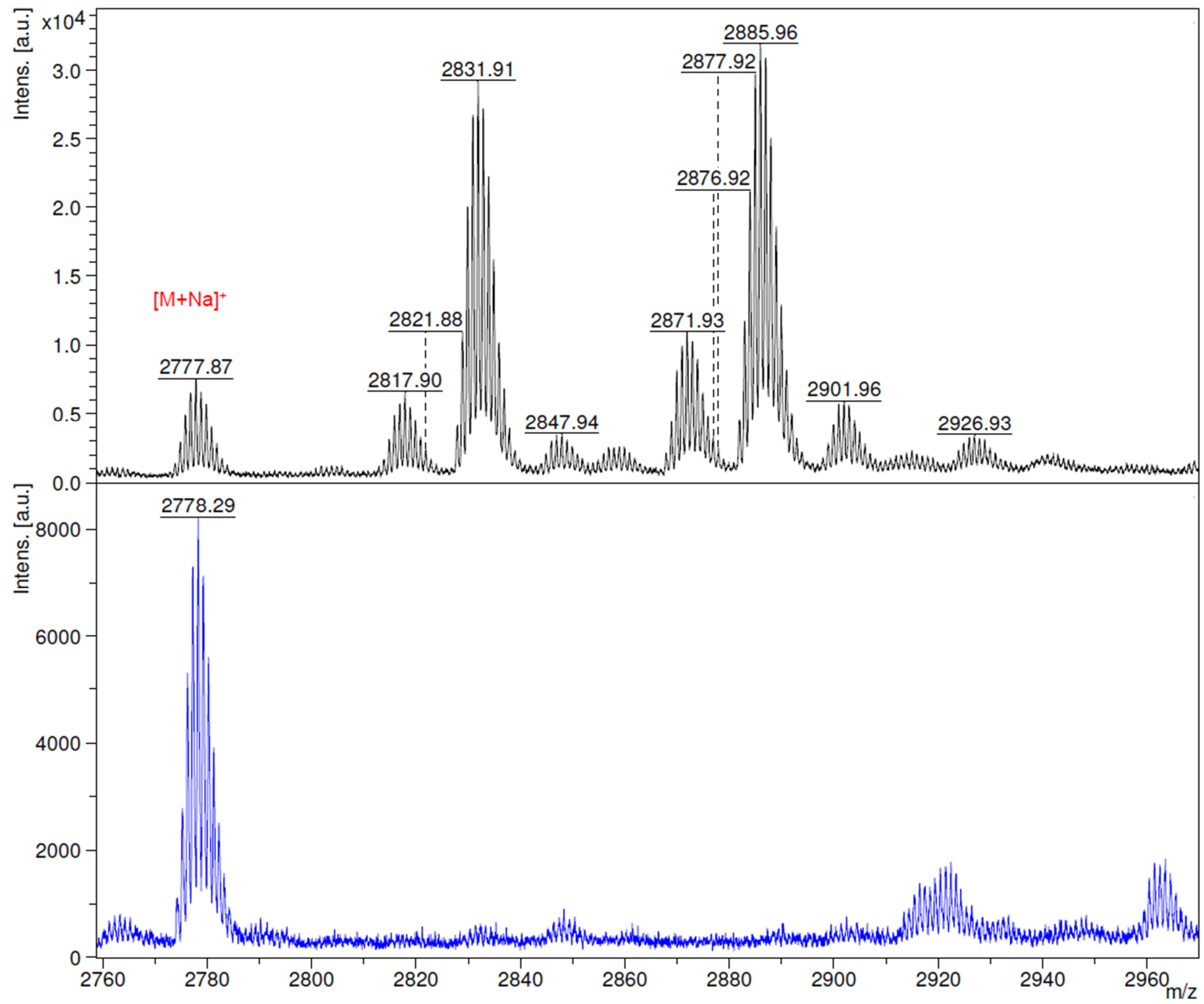

Figure S60: MALDI-ToF-MS spectra of Si_{32}-C_{11} (black: CHCA, blue: DCTB matrix).

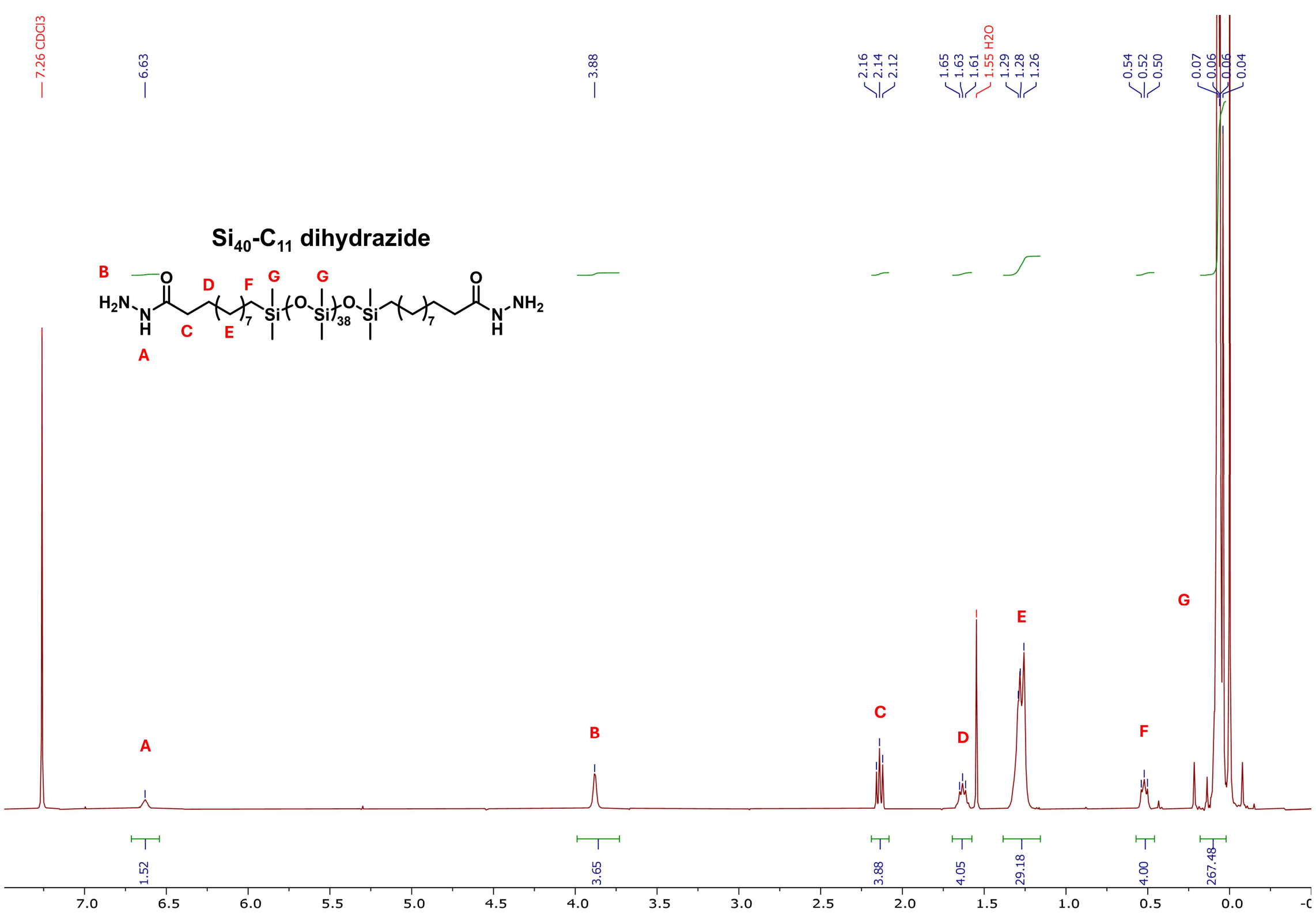

Figure S61: ^{1}H NMR spectrum of Si_{40}-C_{11} dihydrazide in $CDCl_3$.

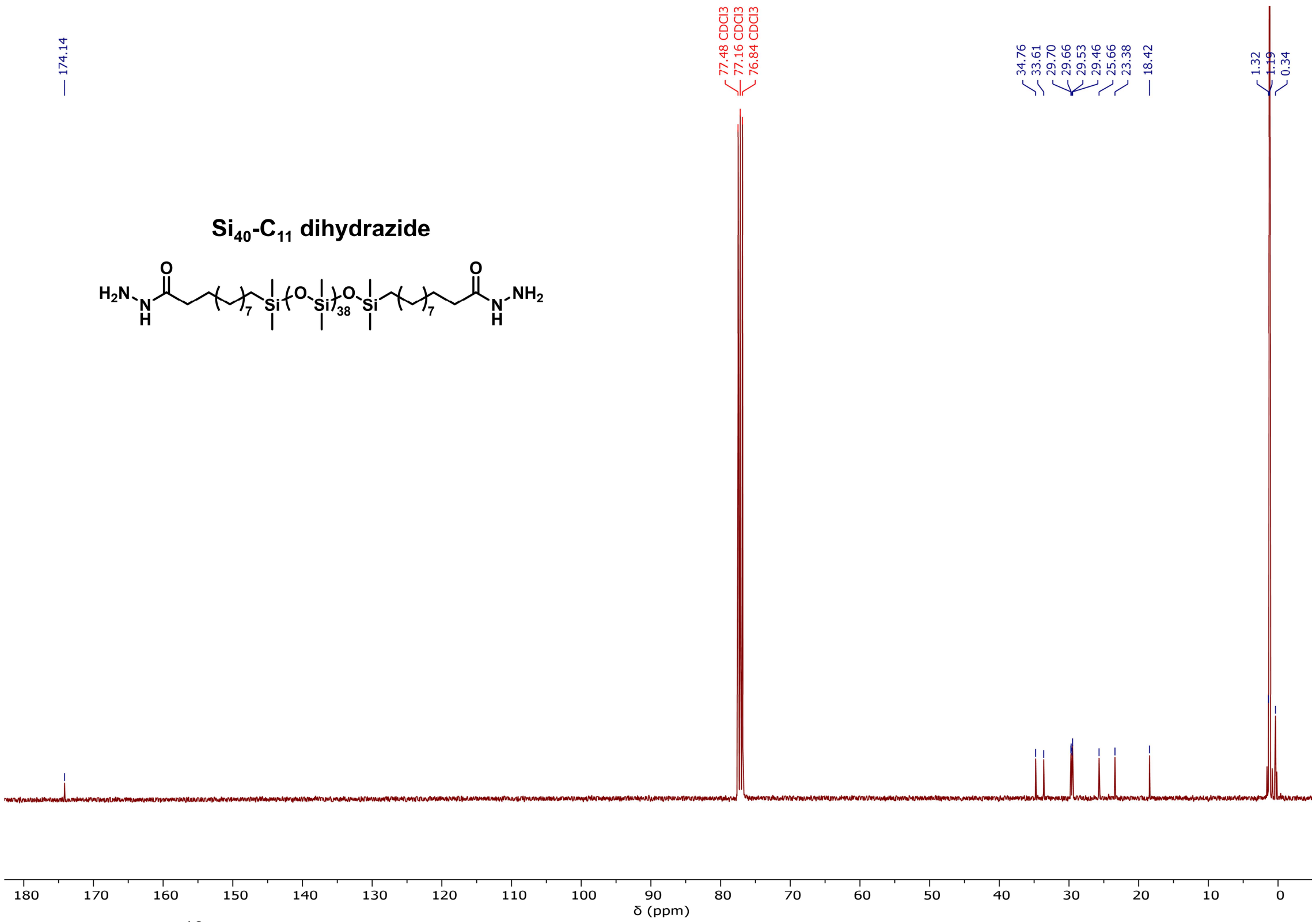

Figure S62: ^{13}C NMR spectrum of Si_{40}-C_{11} dihydrazide in $CDCl_3$.

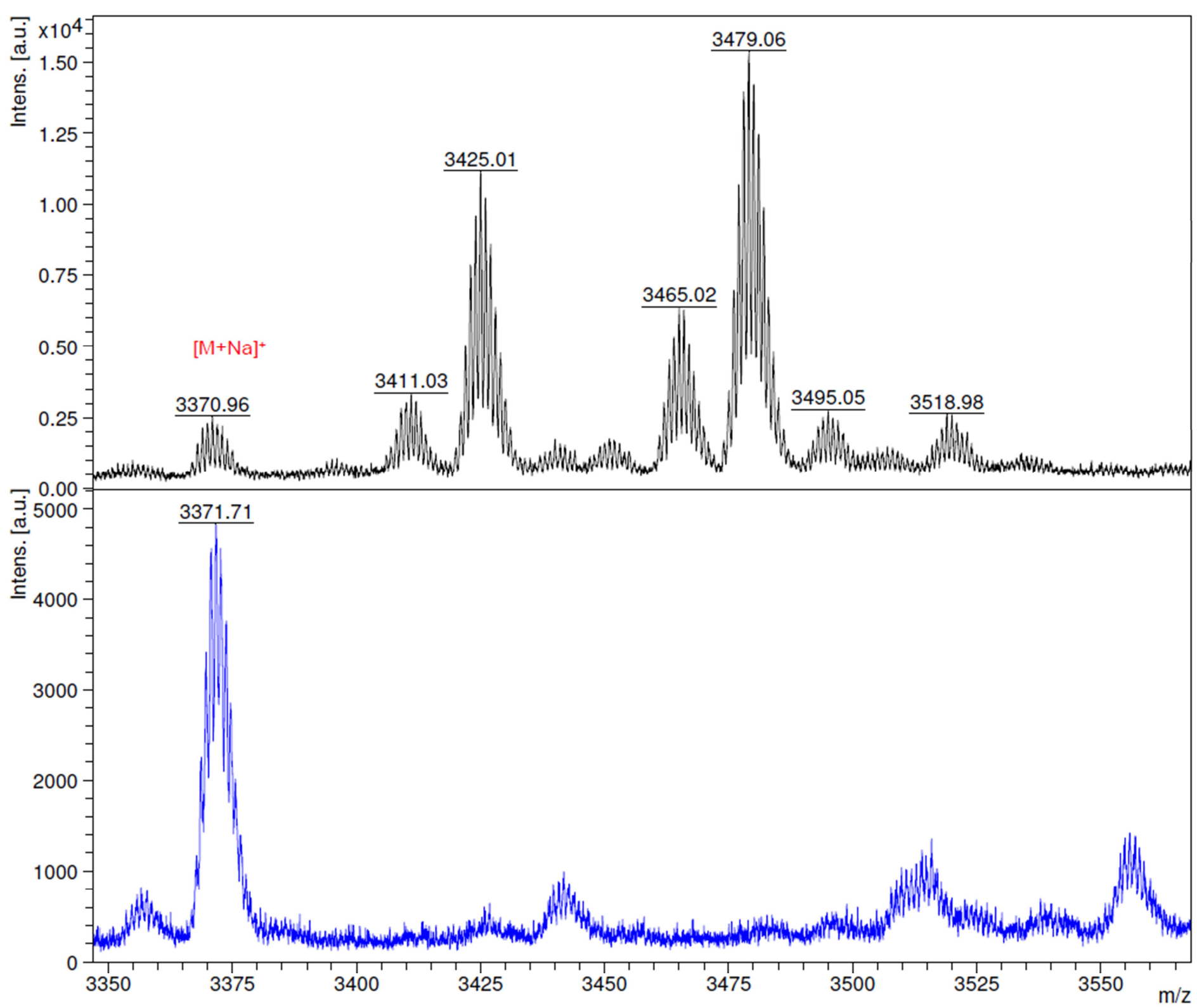

Figure S63: MALDI-ToF-MS spectra of Si_{40}-C_{11} dihydrazide (black: CHCA, blue: DCTB matrix).

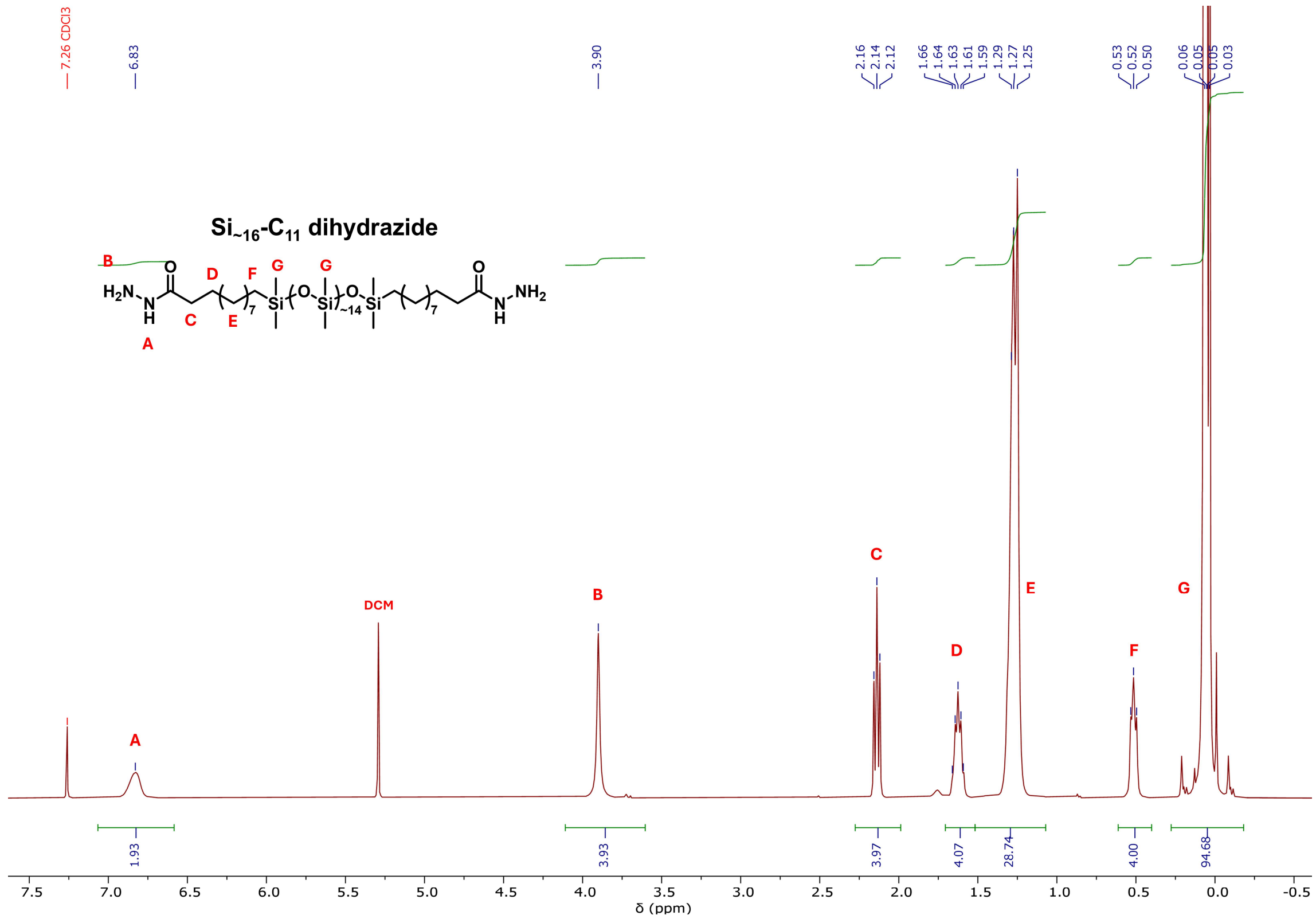

Figure S64: ^{1}H NMR spectrum of Si~16-C11 dihydrazide in $CDCl_3$.

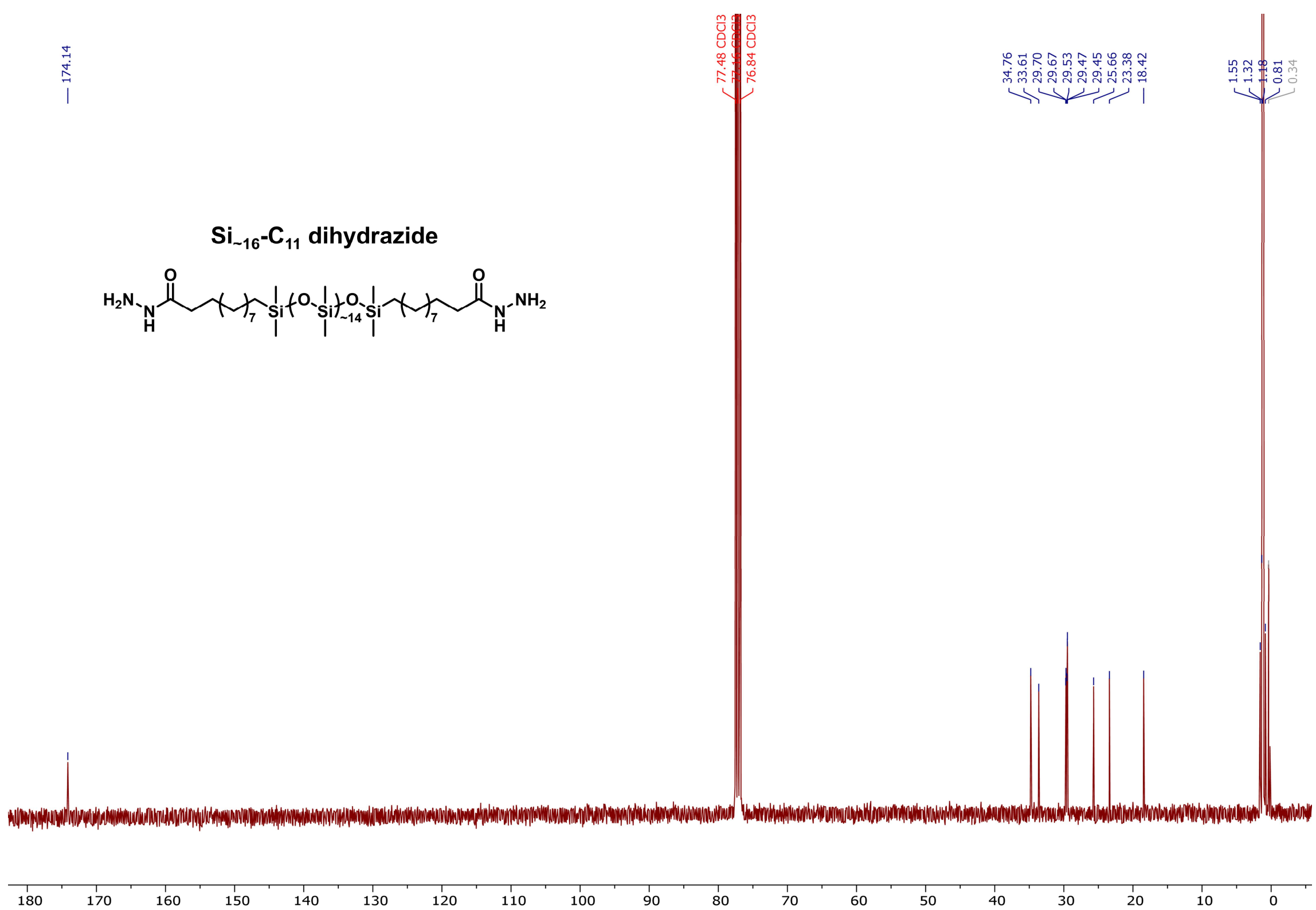

Figure S65: ^{13}C NMR spectrum of Si~16-C11 dihydrazide in $CDCl_3$.

References

[1] B. A. G. Lamers, B. F. M. de Waal, E. W. Meijer, *J. Polym. Sci.* **2021**, *59*, 1142–1150.

[2] R. G. Ricarte, S. Shanbhag, *Polym. Chem.* **2024**, *15*, 815.

[3] S. J. D. Maessen, N. R. Plantinga, R. Wink, B. W. L. van den Bersselaar, J. P. A. Heuts, A. C. C. Esteves, A. R. A. Palmans, *Prog. Org. Coat.* **2026**, *210*, 109684.
